# Structure and composition of early biofilms formed on dental implants are complex, diverse, subject-specific and dynamic

**DOI:** 10.1038/s41522-024-00624-3

**Published:** 2024-12-24

**Authors:** Sophie Dieckow, Szymon P. Szafrański, Jasmin Grischke, Taoran Qu, Katharina Doll-Nikutta, Matthias Steglich, Ines Yang, Susanne Häussler, Meike Stiesch

**Affiliations:** 1 Department of Prosthetic Dentistry and Biomedical Materials Science, Hannover Medical School, Hannover, Germany; 2 Lower Saxony Centre for Biomedical Engineering, Implant Research and Development (NIFE), Hannover, Germany; 3 Cluster of Excellence RESIST (EXC 2155), Hannover Medical School, Hannover, Germany; 4 Department of Molecular Bacteriology, Helmholtz Centre for Infection Research, Braunschweig, Germany; 5 Institute for Molecular Bacteriology, Twincore, Centre for Clinical and Experimental Infection Research, Hannover, Germany; 6 Department of Clinical Microbiology, Copenhagen University Hospital - Rigshospitalet, Copenhagen, Denmark

**Keywords:** Biofilms, Plaque, Next-generation sequencing, Microbial ecology, Microbiome

## Abstract

Biofilm-associated peri-implant infections pose a major problem in modern medicine. The understanding of biofilm development is hampered by biofilm complexity and the lack of robust clinical models. This study comprehensively characterized the dynamics of early biofilm formation in the transmucosal passage of implant abutments in 12 patients. Biofilm structures and compositions were complex, diverse, subject-specific and dynamic. A total of 371 different bacterial species were detected. 100 phylogenetically diverse unnamed species and 35 taxonomically diverse disease-associated species comprised an average 4.3% and 3.1% of the community, respectively, but reached up to 12.7% and 21.7% in some samples. Oral taxa formed numerous positive associations and clusters and were characterized by a high potential for metabolic interactions. The subspecies diversity was highly patient-specific and species-dependent, with 1427 ASVs identified in total. The unprecedented depth of early biofilm characterization in this study will support the development of individualized preventive and early diagnostic strategies.

## Introduction

The formation of biofilms on medical implants is a dynamic and continuous process^1^ that is usually characterized by a healthy balance between oral microorganisms and human tissue. However, biofilm development may cause difficult-to-treat microbial infections in the human oral cavity, such as peri-implant mucositis (PIM) and peri-implantitis (PI)^2–4^. PIM is a reversible inflammatory condition affecting the mucosa around a dental implant, typically characterized by visual signs of inflammation and bleeding on probing^5^. If left untreated, PIM can progress into PI, a more severe condition that affects both the soft tissue and the supporting bone, and can ultimately lead to implant failure.

Dental implants, which are usually made of titanium alloys, serve as a standard method of replacing missing teeth^6^. Within minutes of implantation, the implant surface begins to accumulate oral microorganisms^7^. This is initiated by the formation of a thin protein conditioning film known as the salivary pellicle^8^, followed by attachment of microbial cells^1^ or aggregates^9^. This adhesion process has been analyzed from both a physicochemical and a biochemical perspective, addressing non-specific physicochemical mechanisms and specific ligand-receptor interactions, respectively^10–12^. While adhesion depends on surface properties such as hydrophobicity, free energy, charge, and roughness, which are determined by both material composition and surface modifications^13–16^, it is also influenced by taxon-specific biophysical characteristics of the bacterial cells^17,18^.

Colonization follows a defined sequence of taxa, often associated with specific physical and metabolic interactions that can be modulated by environmental factors^19^. The earliest colonizers on dental implants are predominantly *Streptococcus* and *Actinomyces* species^20^, which typically attach within minutes^7^. During the first weeks, the biofilms are characterized by additional high abundances of the genera *Fusobacterium*, *Neisseria*, *Veillonella* and *Prevotella*^21–23^. Early or secondary bacterial colonizers within biofilms can foster the growth of fastidious anaerobes by lowering the redox potential and releasing growth factors^24^, thus, initiating microbial succession^7,20–22,25^. Over time, implant-associated biofilms become increasingly diverse and reach maturity within a few months^20,25,26^. Mature biofilms are characterized by a fully developed extracellular polymer matrix and a variety of microcolonies, voids, and channels^27,28^. As the matrix serves as a barrier by inhibiting diffusion and as a consequence of mutualistic interspecies interactions, bacteria in biofilms exhibit increased tolerance towards both antibacterial treatment and the host immune defense^29^. Therefore, treatment of established peri-implant infections remains challenging.

The bacterial communities within biofilms on dental implants may initially achieve a symbiotic equilibrium with the host and, thus, are compatible with peri-implant health. However, changes in the microenvironment can lead to taxonomic and functional shifts in the biofilm^30–33^, known as a microbial imbalance or dysbiosis^34^.

The role of the first microbial colonizers in the establishment of microbial dysbiosis is largely unknown, particularly due to a lack of clinically relevant and robust models. In vitro and animal studies offer valuable mechanistic insights but differ from the situation in the human mouth in critical aspects such as the microbial taxa involved^35–38^. In order to capture the complex microenvironment of the human mouth, clinical studies are crucial. This patient-derived data is inevitably influenced by the location of the sampling site as well as the sampling procedure^31,39,40^. Analysis of biofilms collected from implants using instruments such as curettes or paper points can capture the taxonomic biofilm composition at high resolution^22^ but offer little information on the three-dimensional biofilm structure. Retrievable splint systems, on the other hand, allow for investigations into spatial aspects of biofilm parameters using confocal or electron microscopy but typically reflect only supramucosal areas^21,41–48^. To fully reflect the peri-implant environment with supra- and submucosal areas, and to replicate key implant characteristics such as geometry, temporary abutments can be applied^39,49–52^. Previous studies combining temporary abutments with microscopic and molecular analyses of biofilms revealed detailed biofilm structures across space, time and various material types, but so far provided only limited taxonomic analyses^23,53^. Long-read sequencing and advanced computational algorithms that resolve full 16S rRNA gene sequences into amplicon sequence variants (ASVs) present a promising approach to obtain high-resolution data on microbial composition dynamics^54,55^.

In this study, we investigated the evolving structure and composition of biofilms on temporary dental implant abutments, which offer an atraumatic source of early implant-associated biofilms in the area of implant penetration through the mucosa, the site of the highest susceptibility to disease. We employed confocal laser scanning microscopy (CLSM) in combination with full-length 16S rRNA gene amplicon sequencing to reveal biofilm diversity at high resolution (down to ASVs) in the first three weeks after abutment insertion in 12 patients with at least two implants. Furthermore, in order to gain a comprehensive understanding of the dynamics of biofilm formation, we predicted the network of potential ecological relationships between the most abundant biofilm members, thus elucidating system-level interactions. The unprecedented depth of early in vivo biofilm characterization and the comprehensive understanding of interactions within early biofilm development provides the basis for future developments of early diagnostics as well as personalized prevention and treatment strategies for peri-implant infections.

## Results

**Fig. 1:**
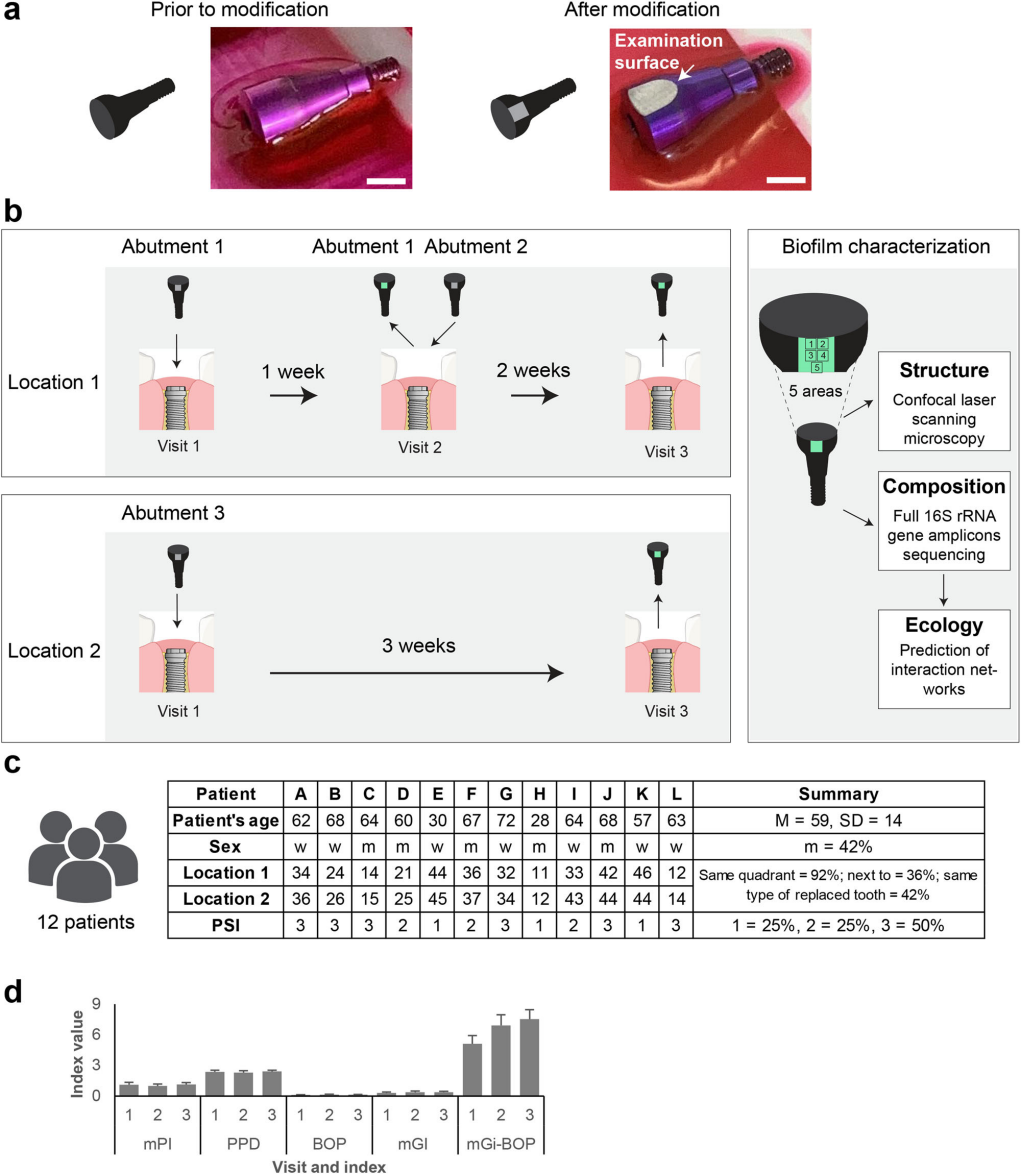
Experimental setting for atraumatic biofilm investigation and clinical parameters. **a** Modified temporary implant abutments with a flat examination surface were manufactured for each patient. Bar: 4 mm. **b** Abutments were inserted at two sites. Biofilm structures were analyzed using confocal microscopy (at five distinct areas), and the composition of the biofilm was characterized through 16S rRNA gene amplicon sequencing. **c** Demographic and clinical data of the participating patients. **d** Dynamics of clinical parameters: Modified Plaque Index (mPI), Probing Pocket Depth (PPD), Bleeding On Probing (BOP), modified Gingival Index (mGI) and Mucositis severity (mGi-BOP). Data refers specifically to implant abutment sites.

In this study, we investigated the development of early biofilms on temporary implant abutments. We included a total of 12 patients, each with two implant sites. Personalized implant abutments (Fig. 1a) were connected to the implants and biofilm analysis was performed after 1, 2 and 3 weeks of biofilm formation (Fig. 1b). While the implant abutments 1 and 2 were placed at the same location, abutment 3 was positioned differently. The biofilm structure on each abutment was analyzed at five distinct areas using confocal microscopy (CLSM) and the microbial composition of the entire biofilm community by sequencing full-length 16S rRNA gene amplicons. The mean age of the patients was 59 years, and 7 were women (Fig. 1c). Half of the patients reported a medical history of previous periodontal treatment. At the timepoint of inclusion into the study, the patients showed no clinical signs of inflammation (Fig. 1d), and all patients were classified as cases of clinical periodontal health according to the classification scheme for periodontal and peri-implant diseases and conditions^56^.

### Biofilm volumes increased over time and microbial colonization was higher in supramucosal implant areas

**Fig. 2:**
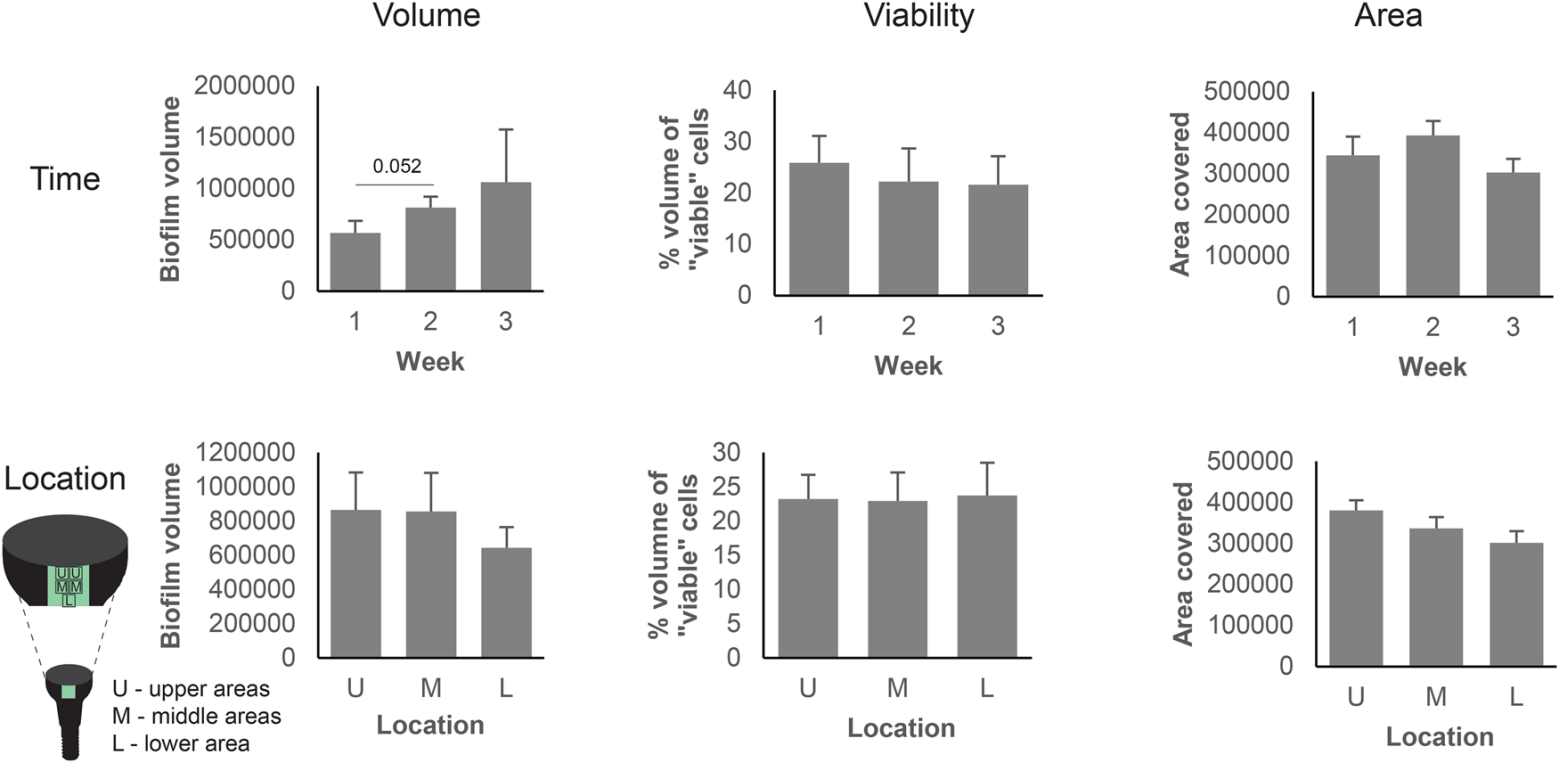
Biofilm parameters across time and space. Biofilm volume, viability and surface area covered were monitored after 1, 2 and 3 weeks (upper graphs) and three locations (lower graphs). Mean and SEM were plotted.

We analyzed the evolution of early biofilm structures as characterized by the three key biofilm parameters: volume, viability and covered surface area, over time by fluorescence staining and CLSM (Fig. 2, Supplementary Fig. 1). A total of 180 biofilm images (12 patients × 3 time points × 5 distinct areas) were captured. High interpersonal diversity was observed in the parameter dynamics (Supplementary Fig. 1c–e). The mean biofilm volume increased by 44% between the first and second week (right-tailed paired *t*-test; *p* = 0.052), followed by a further 30% increase between the second and third week (Fig. 2). In most cases, biofilm volume ranged from 0.1 × 10^6^ to 1.5 × 10^6^ µm^3^ per image, with one exception showing massive biofilm growth after three weeks (Supplementary Fig. 1c). Mean biofilm viability initially decreased before stabilizing at ~20% viable cell volume (Fig. 2). The mean surface area covered by the biofilm exhibited a slight increase between the first and second week before declining. In most cases biofilm volume grew most prominently in the supramucosal implant areas (upper versus lower areas), while the mean viability was generally uniform across the areas studied.

### Biofilm structures were patient-specific and evolved over time

**Fig. 3:**
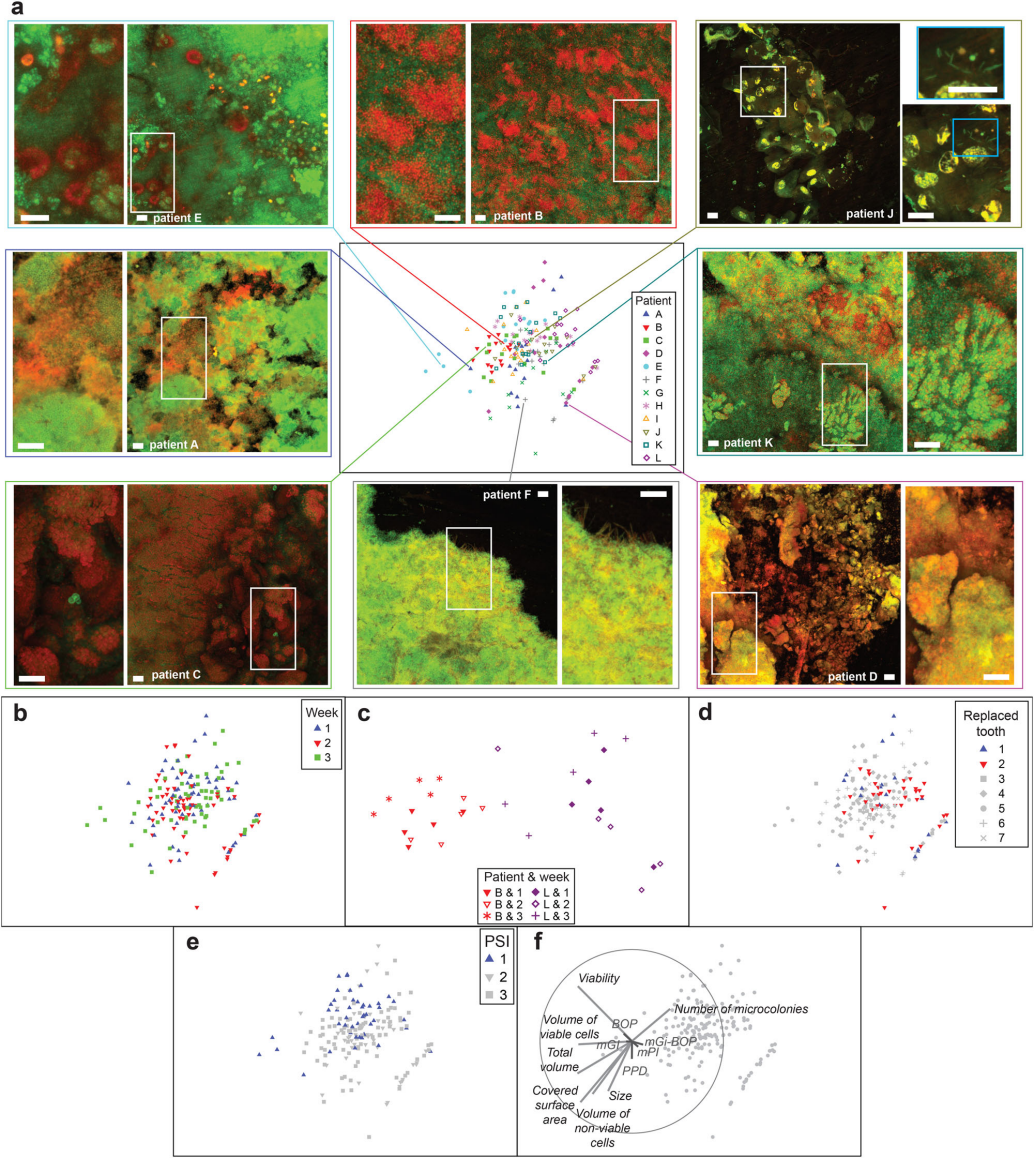
Biofilm structure profiles generated by confocal microscopy. Confocal laser scanning microscopy (CLSM) was performed for biofilms stained with a life/dead stain. **a** Non-metric Multi-Dimensional Scaling (nm-MDS) of 180 biofilm structure profiles captured from twelve patients, three time points, and five biofilm areas. Seven parameters –biofilm volume, red (dead) cell volume, green (live) cell volume, percent of live cell volume, number and size of microcolonies, and surface area covered by biofilm – were standardized by maximum and fourth root transformation prior to pairwise calculations of Euclidean distances. Symbols of different shape and color indicate different patients. Eight examples of confocal images are presented. Bar indicates 40 µm. **b** Symbols indicate age of the biofilms. **c** Enlarged nm-MDS of biofilm structure profiles of two selected patients. 2D-stress was 0.06. **d** Symbols indicate implant location. **e** Symbols indicate the Periodontal Screening Index (PSI). **f** Superimposed is a vector plot for biofilm (in grey) and clinical (in red) parameters, with the vector direction for each class reflecting the Pearson correlations of their values with the ordination axes, and length giving the multiple correlation coefficient from this linear regression on the ordinate points.

Evaluation of biofilm micrographs revealed cell aggregates, clusters and microcolonies (Fig. 3a and Supplementary Fig. 2). Common findings included voids and channels, which enable the exchange of nutrients and waste products within the biofilms. However, the size, shape, and density of the cell assemblies varied considerably. Epithelial cells or their remnants were often observed, in some cases with no or little microbial cells. In addition, unique distinct features were detected (e.g., scaffolds formed by a network of bundles consisting of long filamentous cells or chimney structures).

The diversity of distinct biofilm structure parameters across the total of 180 biofilm images was further assessed by multivariate analysis (Fig. 3a–f). We found a patient-specific impact on the structural properties of the biofilms (Fig. 3a, PERMANOVA, pseudo-*F* = 8.4, *p* = 0.0001), as well as an impact of the time of biofilm formation (Fig. 3b, PERMANOVA, pseudo-*F* = 3.8, *p* = 0.0001). This is exemplified in Fig. 3c, where a smaller subset of data is plotted (i.e., representing only 2 of 12 patients). The biofilm profiles of these two patients clustered together and were distinct, while in both patients the biofilms that were sampled at the same time were more similar. Moreover, anatomical location and oral health status had discernible effects on biofilm structures (Fig. 3d, e). Biofilm profiles indicating reduced colonization were frequently observed in implants replacing front teeth numbers 1 and 2 (Fig. 3d) as well as in implants from patients with a PSI of 1 (Fig. 3e, PERMANOVA, pseudo-*F* = 5.7, *p* = 0.0001, *t*_PSI=1 vs PSI=2_ = 3.0, *p* = 0.0001, *t*_PSI=1 vs PSI=3_ = 2.9, *p* = 0.0002, *t*_PSI=2 vs PSI=3_ = 1.1, n.s.). Local clinical parameters, such as BOP, mGI, PPD, mPI and mGi-BOP did not show significant correlations with biofilm profiles (Fig. 3f). Biofilms covering larger areas usually had a larger volume dominated by non-viable (i.e., permeable) cells and a lower number of microcolonies (Fig. 3f). In summary, early biofilm structures varied by patient, time and space.

### Streptococcus, *Actinomyces*, *Schaalia* and *Veillonella* genera were found in all biofilm samples

**Fig. 4:**
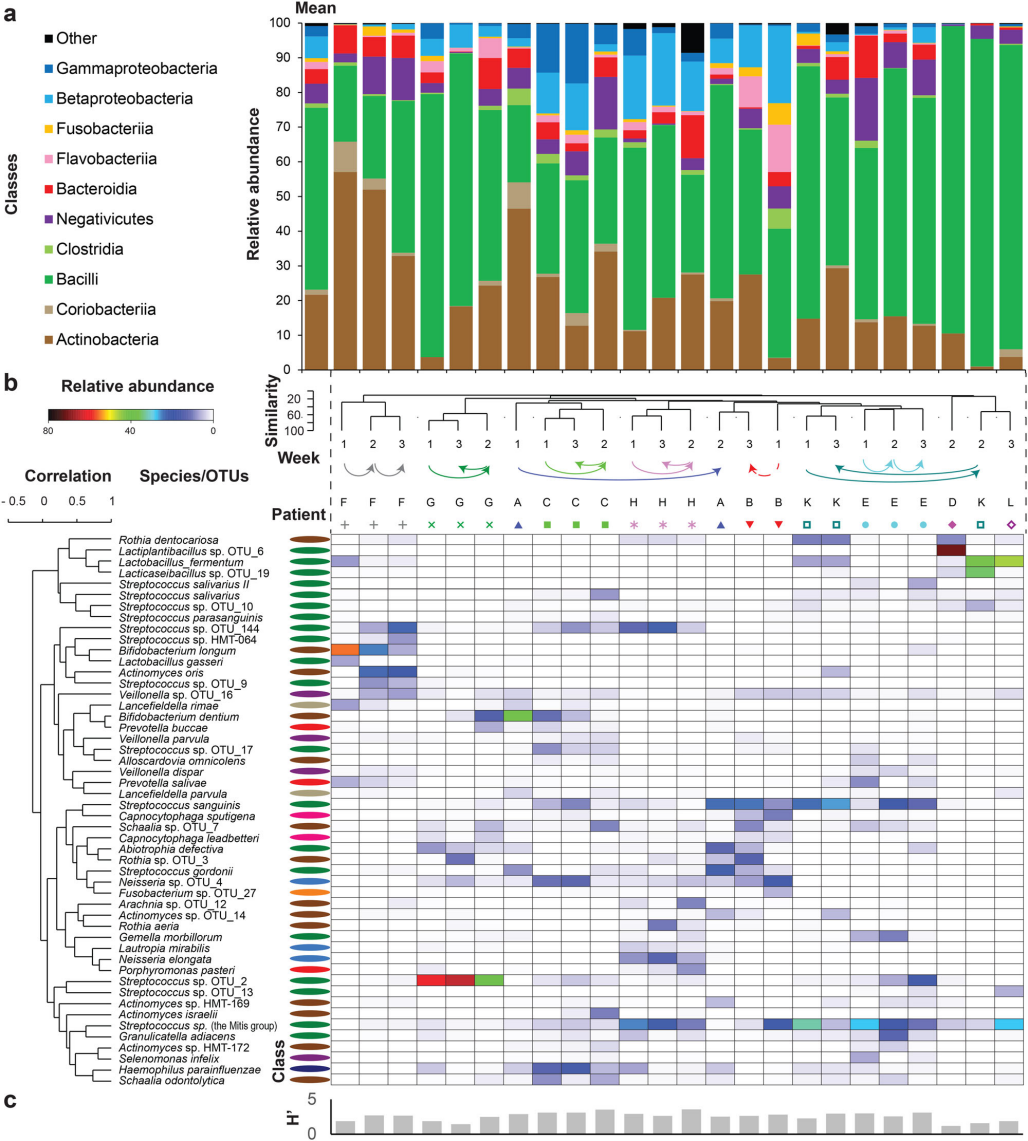
Biofilm composition is individual-specific. Taxonomic diversity of early biofilms as assessed with full 16S rRNA gene amplicons sequencing. **a** Relative abundance of reads grouped at the class level and plotted for each patient-time point combination as well as for average profile (first from left). Top ten abundant classes are shown, while the reads matching other classes were summed up and plotted together as “Other”. **b** Heatmap shows the relative abundance of selected species. Samples and species were clustered. For each sample, the time point and patient are depicted above the heatmap. Values for five diversity indices for each sample were plotted below heatmap. For each species the classification at the class level is indicated. **c** Values for Shannon diversity index were plotted below the heatmap.

In addition to CLSM, we applied full-length 16S rRNA gene amplicon sequencing to unveil biofilm diversity at high resolution. From 10 patients, at least one successfully sequenced biofilm sample could be analyzed. At the class level, we observed a strong patient-specific effect (PERMANOVA, pseudo-*F* = 3.2, *p* = 0.0008). Members of the Bacilli class, predominantly streptococci, overwhelmingly dominated early biofilms, reaching a minimum relative abundance of 22% in each sample (Fig. 4a, Supplementary Fig. 3a, b). Actinobacteria emerged as the second most abundant class (comprising *Actinomyces* and the closely related *Schaalia*), although their prevalence varied significantly across samples. Eleven other classes reached up to 5% relative abundance in individual biofilms. Among them, the Negativicutes (primarily *Veillonella*) was the only class found in all samples, in addition to the aforementioned genera *Streptococcus*, *Actinomyces* and *Schaalia* (Supplementary Fig. 3c). The prevalence of classes including potentially pathogenic microorganisms was generally low, except for a single biofilm sample, where Betaproteobacteria, Fusobacteriia, Flavobacteriia, Bacteroidia and Clostridia collectively constituted 60% of relative abundance (Fig. 4a, sample B1). Five genera, including *Actinomyces* and *Neisseria*, displayed a tendency to proliferate over time, while six genera demonstrated the opposite trend (Supplementary Fig. 3d). The abundances of *Granulicatella*, *Gemella* and *Schaalia* were positively correlated with biofilm viability (Supplementary Fig. 3e, f). Few genera appeared to play a major role in the composition of the observed biofilms.

### Bacterial species biofilm composition was patient-specific and evolved over time

In total 371 different species-level phylotypes were identified, with a mean of 92 species per implant (SD = 3; range 28–148). Based on the abundance of the top 50 species, biofilms clustered by patient rather than by time or the overall health status of the patient’s mouth (Fig. 4b). Abundant taxa displayed consistent colonization over time, although their relative abundances fluctuated. *Streptococcus* spp. from the Mitis group were the most abundant operational taxonomic unit (OTU) and together with *Veillonella* sp. OTU_16 (best matching *Veillonella parvula* and *Veillonella dispar*) was detected in all individual biofilms. Other notably abundant species included *Streptococcus* sp. OTU_2 (best matching *Streptococcus sanguinis*), *S. sanguinis*, *Lacticaseibacillus* sp. OTU_19 (best matching *Lacticaseibacillus paracasei*), *Actinomyces oris* and *Streptococcus gordonii*. Strict anaerobes, such as *Veillonella* sp. OTU_16, *Prevotella salivae* or *Lancefieldella rimae* (formerly *Atopobium rimae*) were present in smaller numbers. Microbial diversity (as reflected by *H’*) generally remained high and stable across patients and time points, except for three biofilms dominated by lactobacilli and a single biofilm dominated by streptococci (Fig. 4c).

### Unnamed species were important components of early biofilms

**Fig. 5:**
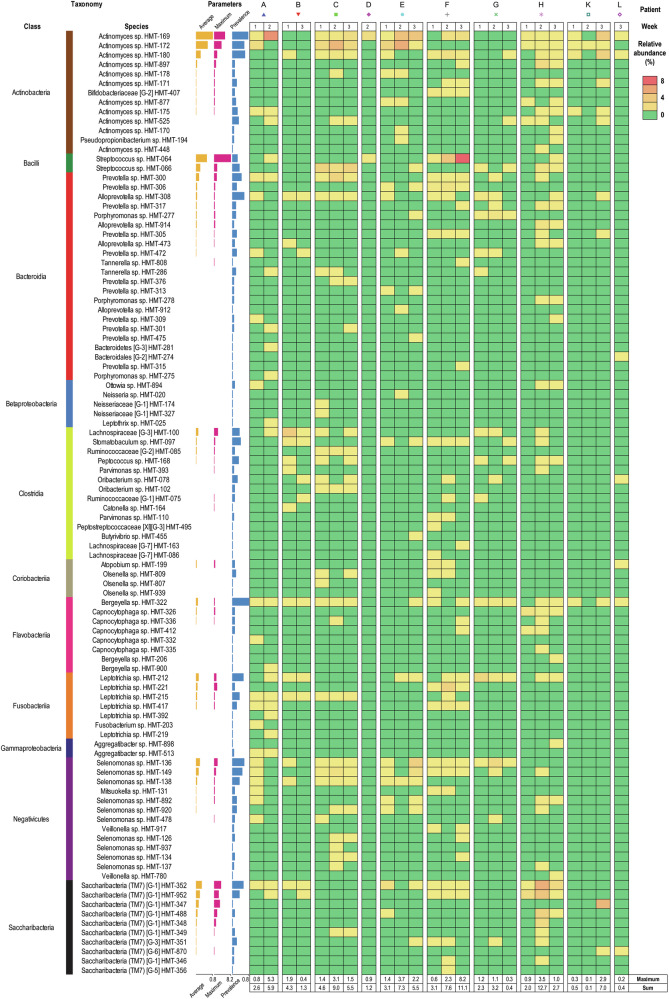
Not-yet-named hard-to-culture species identified in early implant-associated biofilms. Not-yet-named hard-to-culture species identified in early implant-associated biofilm samples were sorted by mean relative abundance and grouped by taxonomy at the class level. Following information is given (from left): taxonomy at the class level, species name, mean relative abundance (yellow bars), maximum abundance (pink bars), and prevalence (blue bars), relative abundance plotted for every sample (heat map). Samples were sorted by time and by patient. Red indicates the highest abundance, yellow marks intermediate values and green zeros. Maximum values and sums are reported for each sample below heat map.

Within the human oral cavity, 774 species have already been identified, of which 42% are still unnamed or have uncultivated phylotypes (Expanded Human Oral Microbiome Database V3.1, accessed on 23.05.2024 at www.homd.org)^57^. Such unnamed species were frequently identified in the implant-associated biofilms of this study, corresponding to a mean of 4.3% of the community, with a maximum of 12.7% (Fig. 5). Many of these unnamed species displayed consistent colonization across multiple individuals and were members of taxonomically diverse groups. Clostridia and Bacteroidia encompassed the highest count of genera (11) and species (22), respectively. Interestingly, Bacteroidia include the commensal *Porphyromonas* and *Tannerella* species, representing genera best known for some of the most important microorganisms associated with periodontal and peri-implant diseases. Abundant species included *Actinomyces* spp. HMT-169, HMT-172 and HMT-180, *Leptotrichia* sp. HMT-212, Lachnospiraceae [G-3] sp. HMT-100, *Prevotella* sp. HMT-300, *Selenomonas* spp. HMT-136 and HMT-149, *Streptococcus* sp. HMT-064 and HMT-066, and Saccharibacteria (also known as TM7, encompassing provisional taxa) spp. HMT-352, HMT-352, HMT-347 and HMT-952, the latter with a provisional name [*Nanosynbacter lyticus*]. Although *Riemerella* (formerly *Bergeyella*) sp. HMT-322 was the most prevalent unnamed species in our dataset, its abundance was consistently very low. Unnamed Clostridia, Bacteroidia and members of few other classes were diverse but usually minor components of early biofilms.

### Potential pathogens were commonly detected albeit in low abundance

**Fig. 6:**
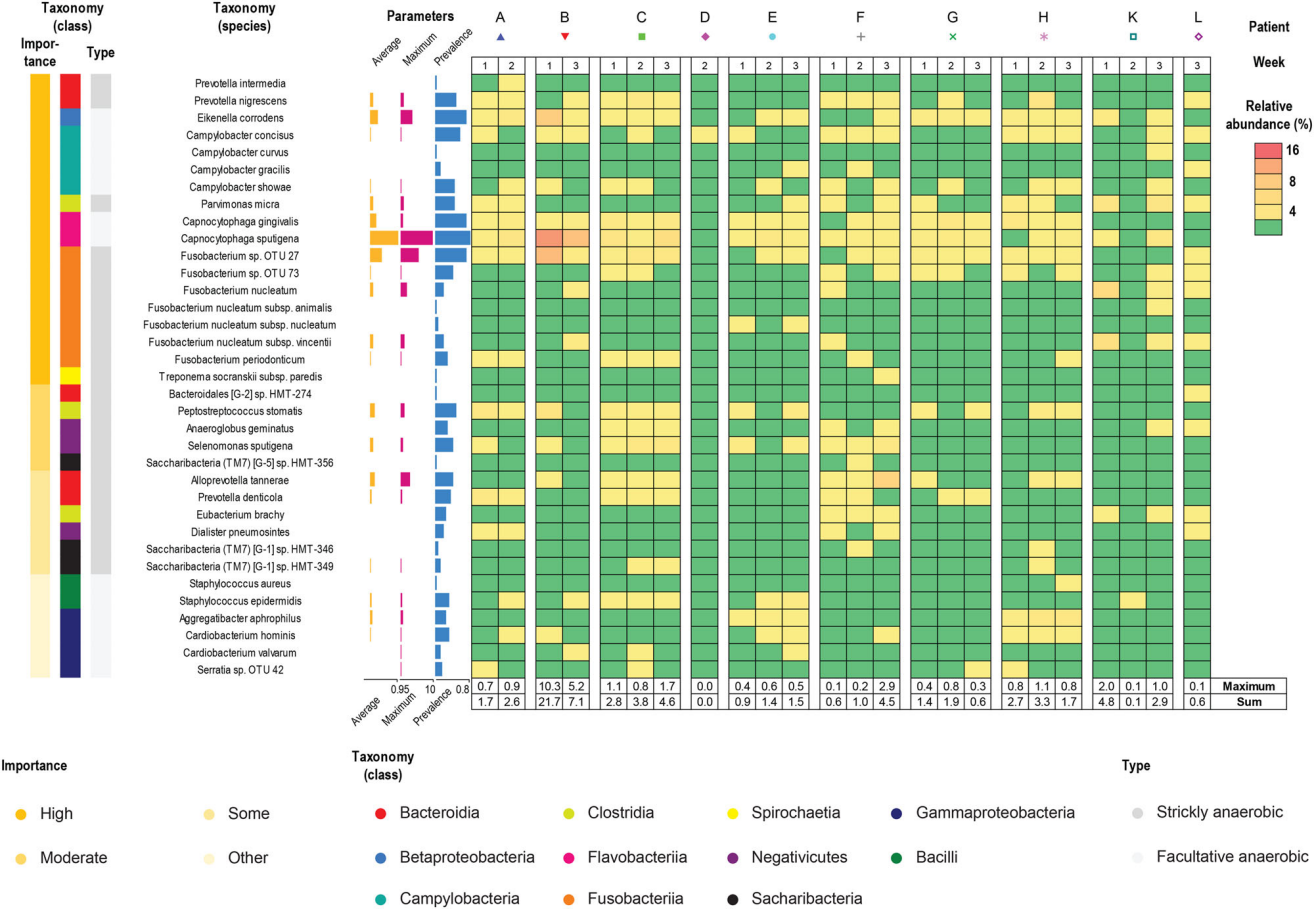
Potentially pathogenic species identified in early implant-associated biofilms. Potential pathogens identified in early implant-associated biofilm samples were sorted by taxonomy at the class level and importance. The following information is given (from left): importance, taxonomy at the class level, type of growth, species name, average relative abundance (yellow bars), maximum abundance (pink bars), and prevalence (blue bars), relative abundance plotted for every sample (heat map). Samples were sorted by time and by patient. Red indicates the highest abundance, yellow marks intermediate values and green zeros. Maximum values and sums are reported for each sample below heat map.

Subsequently, we examined the relative abundance of microorganisms associated with periodontal or peri-implant diseases in the early biofilms (Fig. 6). A total of 35 diverse potentially pathogenic species, representing nine classes, were commonly observed, collectively accounting for a mean of 3.1% of the community, with a maximum of 21.7%. Only a few species displayed consistent colonization across multiple individuals. Both facultative and strictly anaerobic microorganisms with pathogenic potential were present in early biofilms, and often co-existed. *Capnocytophaga sputigena*, *Fusobacterium* sp. OTU 27 (best matching *Fusobacterium periodonticum*) and *Eikenella corrodens* emerged as the most abundant potential pathogens. These species were detected in one patient across two different implants. However, no pathological changes were noted at these sites during the experimental period. Notably, key periodontal pathogens such as *Porphyromonas gingivalis*, *Tannerella forsythia*, *Treponema denticola* and *Aggregatibacter actinomycetemcomitans* were absent. Allochthonous potentially pathogenic microoganisms like *Staphylococcus* spp. and *Serratia* sp. were detected but were present at very low levels. Potential pathogens appeared to be diverse but play only a minor role in the composition of the observed biofilms.

### Oral taxa formed stable positive associations and showed high potential for engagement in interspecies interactions

**Fig. 7:**
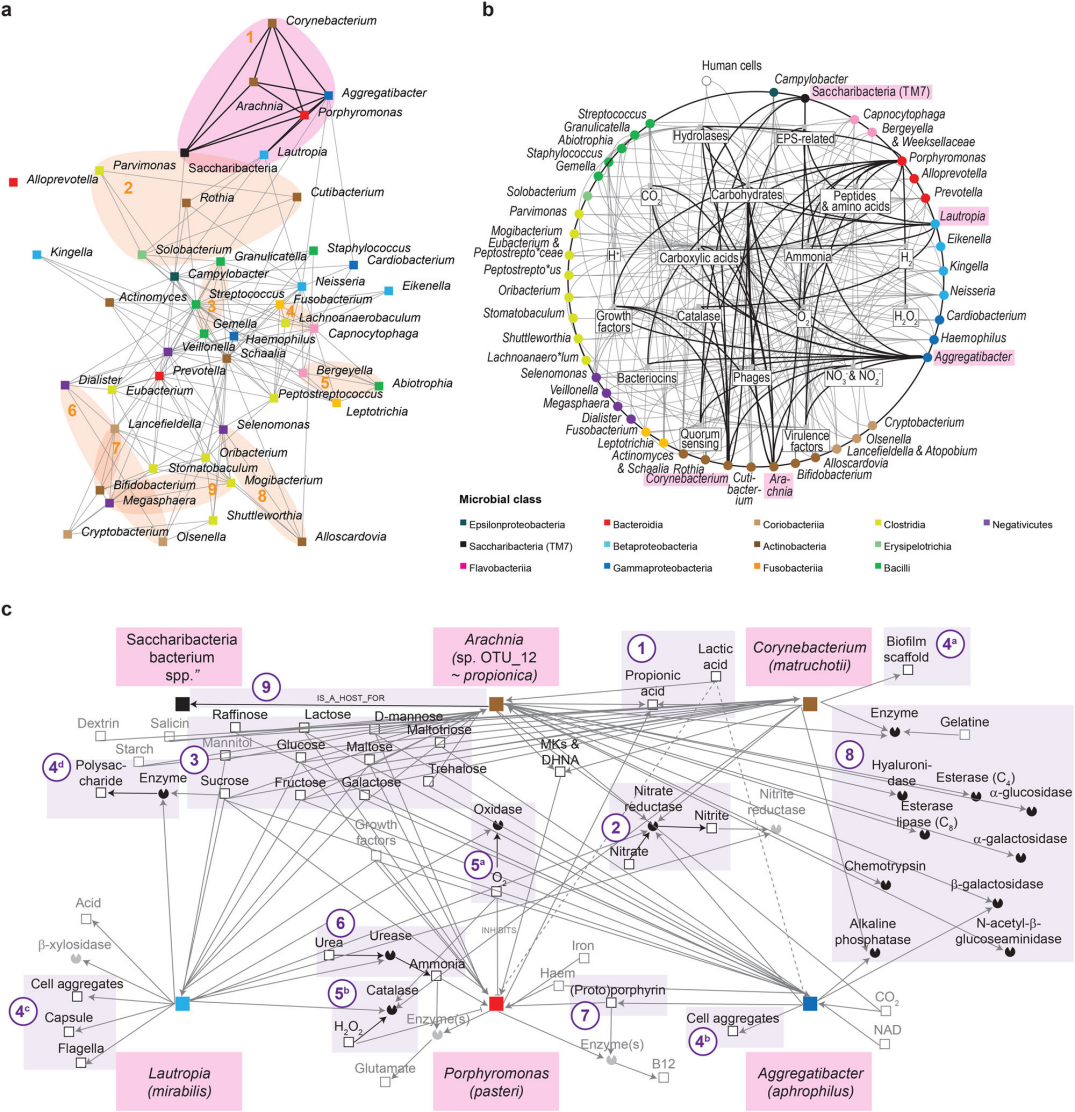
Relationships between genera in early biofilms. **a** Associations between top 50 genera. Ordinate visualizes the results of R-mode analysis using pairwise Whittaker’s associations. Five genera related to *Lactobacillus sensu lato* were identified as outliers and were omitted from ordination. Links represent associations >0.4. SIMPROF clusters were highlighted in pink (cluster with the highest number of connections) or in orange (all the other clusters). For more details see Supplementary Fig. 4 and M&M section. **b** Interactions in early biofilms. Intergeneric interactions were inferred using custom-made database summarizing curated phenotypic information for top 50 genera. Nodes representing genera were placed on a circle and sorted based on phylogeny (see Supplementary Fig. 5). Members of cluster with the highest number of connections are highlighted in pink and their connections are shown as thick lines. Nodes inside circles represent metabolites, enzymes and other biofilm components aggregated at 18 ecological classes. **c** Ecological interactions between *Aggregatibacter*, *Arachnia*, *Corynebacterium*, *Lautropia*, *Porphyromonas* and *Saccharibacteria* bacterium species which clustered together. Ecological information was retrieved for the most abundant species representing the aforementioned genera. Most important subnetworks were highlighted. (1) Short chain fatty acids. (2) Nitrate reduction. (3) Carbohydrate catabolism. (4) Biofilm re-modeling. (5) Oxidative stress. (6) Alkalinization. (7) Porphyrin metabolism. (8) Hydrolases. (9) Parasitism involving epibiont. Characteristics of relationships (e.g., labels and attributes reflecting prevalence, intensity, etc.) were omitted for clarity. See Results and Discussion for more details.

Networks between genera of the identified bacteria were analyzed to predict potential ecological relationships between the most abundant biofilm members in early biofilms. Microorganisms that grouped at the genus level exhibited statistical clustering (type 2 SIMPROF test, *p* < 0.001) within early biofilms (Fig. 7a, Supplementary Fig. 4c, d). Taxa that demonstrated high association were highlighted on the ordination (Fig. 7a). Ten clusters were identified along with numerous connections. For clarity, a single outlier cluster including lactobacilli was omitted from the ordination, in comparison to the clustering shown in Supplementary Fig. 4d. The largest cluster contained *Aggregatibacter*, *Arachnia*, *Corynebacterium*, *Lautropia*, *Porphyromonas* and Saccharibacteria, which were strongly interconnected. Among the smallest clusters, the strongest associations was recorded between *Bifidobacterium* and *Lancefieldella*. Positive associations were also observed on other taxonomic levels (Supplementary Fig. 4). From the genera clustering in the early biofilms, ecological networks were constructed to decipher positive associations between genera and achieve a comprehensive system-level understanding of early biofilms (Fig. 7b). Numerous connections were identified (e.g., for the members of the largest cluster), some of which aligned closely with the associations observed in vivo (Fig. 7a–c). This consortium was predicted to encompass mostly carbohydrate-oriented, aero-tolerant propionate producers, which are able to either ferment lactate or use nitrate for anaerobic respiration or both (Fig. 7c, Supplementary File 1). Multiple members were predicted to impact the biofilm structure by forming scaffolds or producing polymeric substances. Connections between members included potential cross-protection from oxygen or hydrogen peroxide, production of ammonium that could enter the anabolic pathways, exploitation of the porphyrin pool, sharing hydrolases and metabolic flow between a parasitic epibiont and its host (Fig. 7c).

Subsequently, we examined individual networks at the species level (Supplementary Fig. 5, Supplementary File 1). The number and composition of selected interacting abundant species, enzymes and metabolites varied considerably across individuals. Simple and complex networks were predicted. Common interactions included food chains (involving lactate), exploitation of growth factors (e.g., vitamins, amino acids and inorganic compounds), enzyme sharing (fucosidases, sialidases, peptidases and catalases) and cross-protection (oxygen and hydrogen peroxide depletion).

### Subspecies diversity of early biofilms was highly patient specific and varied considerably between different abundant species

**Fig. 8:**
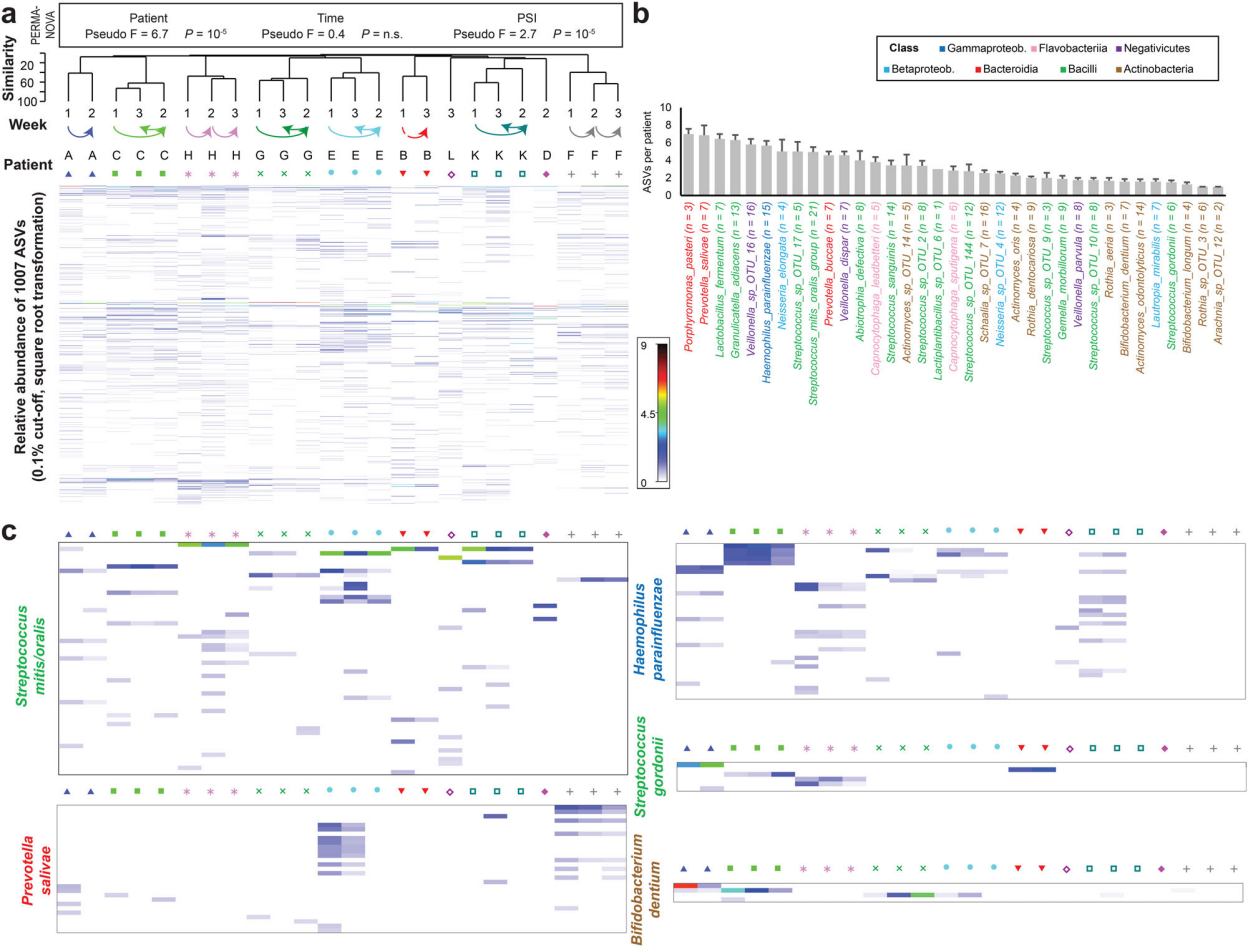
Taxonomic diversity of early biofilms assessed using Amplicon Sequence Variants (ASVs). **a** The heatmap illustrates the relative abundance of the most prevalent ASVs across all samples. Both samples and ASVs were hierarchically clustered. The time points and corresponding patient identifiers are indicated above the heatmap. **b** The bar graph displays the number of ASVs per patient for the 35 most abundant species, sorted by decreasing mean ASV count and colored by class. For each patient, only species with at least 50 reads were included, and the number of patients meeting this threshold is indicated in parentheses following the species name. **c** Heatmaps are shown for representative species with high ASV diversity (e.g., *Streptococcus mitis/oralis*, *Prevotella salivae*, *Haemophilus parainfluenzae*) and low ASV diversity (e.g., *Bifidobacterium dentium*, *Streptococcus gordonii*), following the same format as (**a**).

To capture subspecies diversity, we applied Amplicon Sequence Variant (ASV) analysis (Fig. 8). A total of 1427 ASVs were identified, with 1007 reaching a relative abundance of at least 0.1% in one or more samples. The biofilm profiles clustered distinctly by patient (Fig. 8a), showing a clear effect of PSI, but no significant effect of time. The highest number of ASVs was observed for *Streptococcus mitis/oralis* (61 ASVs), followed by *Veillonella* sp. OTU_16 (40 ASVs) and *Haemophilus parainfluenzae* (43 ASVs) (Supplementary Fig. 6a). Species from the classes Bacteroidia (*Porphyromonas pasteri* and *Prevotella salivae*) and Gammaproteobacteria (*H. parainfluenzae*) showed high ASV numbers per patient, while species from most other classes were highly variable, with Actinobacteria (e.g., *Bifidobacterium* and *Rothia* species) typically displaying lower ASV counts. A sequencing depth cut-off of 50 reads per species was applied to avoid underestimation of ASV counts in less abundant taxa (Supplementary Fig. 6b). Representative species profiles are shown in Fig. 8c. Using re-sampled data (Supplementary Fig. 6c), we demonstrated that ASV diversity varied significantly across patients (Supplementary Fig. 6d), and ASV evenness, as indicated by the Simpson index, increased over time (Supplementary Fig. 6e).

## Discussion

In this study, using an in vivo model that allowed monitoring of early biofilm formation on dental implants in the clinically relevant area of transmucosal passage, we demonstrate for the first time at high resolution that both the structure and composition of early biofilms forming on dental implants are complex, patient-specific and dynamic.

The biofilms for this study were collected from temporary implant abutments that can be completely removed, allowing atraumatic access to the intact biofilm on the implant material. In contrast to other in vivo biofilm models using plastic splints for supramucosal biofilm collection^41,47,48^, our model has a number of advantages, including the localization of the biofilm in a clinically relevant microenvironment, which allows the investigation of both sub- and supramucosal biofilms. In comparison to the studies collecting supramucosal biofilms on splints^48^, we observed a higher abundance of anaerobes from genera *Veillonella* and *Prevotella* that can better thrive in submucosal areas.

In comparison to previous studies employing implant abutments^23,49^, we included a larger cohort of patients and included both quantitative measurements and time course observations. Moreover, by using the same sample for state-of-the-art CLSM-based analysis of the three-dimensional biofilm structure directly on the implant surface as well as for full-length 16S rRNA amplicon sequencing (followed by Amplicon Sequence Variant analysis), a more detailed characterization of early implant-associated biofilm formation was possible than with phase contrast microscopy and detection methods employing DNA probes or taxon-specific PCRs^58–61^. This method allowed us to link structural features to species composition. While analyzing CLSM data and microbiome composition in the same samples might influence the detected biofilm composition, the strong patient-specific signal captured in our study suggests consistent outcomes. Another advantage of our approach is the ability to generate three consecutive biofilm samples from the transmucosal implant areas of each patient, allowing for direct comparisons of biofilm characteristics over time in a realistic clinical setting while minimizing the confounding effects of patient-to-patient variability. The implant abutments were placed at two different implant sites to examine the two-week and the three-week biofilm at the same visit, which minimized the influence of time-dependent factors^62^, such as nutritional changes^63,64^. On the other hand, this procedure could have led to site-specific effects due to spatial gradients within the oral cavity^65^. However, as implants were localized at adjacent sites, albeit in some cases separated by a small edentulous gap, this effect was minimized in 83% of our patients. The model provided valuable insights into early biofilm dynamics in this study and in the future, can also be used for the evaluation of antimicrobial surfaces in vivo. In addition, the in vivo-grown biofilm provides the basis for mechanistic laboratory studies on clinically relevant patient biofilms.

In our study, biofilm structure was analyzed by fluorescence staining and CLSM and quantified for standard parameters^48,66^. As the implant abutments were modified for CLSM investigations, the flattened examination site exhibited slightly different surface properties than the unmodified abutment surface, which may potentially influence biofilm formation^67,68^. However, this effect would primarily impact bacterial adhesion during the initial days and is minimized once the biofilm begins to increase in thickness in the course of the first days. The observed biofilms in our study increased steadily during the first three weeks after abutment placement, were higher in the supramucosal areas, contained various biofilm structures and epithelial cells. Additional non-metric multi-dimensional scaling revealed that these structural features were mainly patient-specific. Similar heterogeneous microbial aggregates have already been observed in early^69–71^ and mature biofilms^72–74^ on teeth. Eukaryotic cells have been observed in dental plaque^74^ and in salivary aggregates that initiate biofilm development^9^, but their role in biofilm development on implants is still poorly understood. Microbes may take advantage of antioxidant defenses or death-induced release of nutrients by epithelial cells. The potential role of these mechanisms in the spread of implant-associated biofilms should be investigated in organotypic models^75–77^ in the future. In the context of microscopic biofilm analyses, the differentiation of non-biofilm structures such as human cells and the limited taxonomic resolution of live/dead staining may pose challenges. However, the methodology employed in this study successfully generated structural profiles that captured patient-specific features.

To investigate the species forming the respective biofilm, complementary high-resolution 16S profiles were generated for the same biofilm samples. Almost four hundred different species were detected in early biofilms on dental implants, thus illustrating the enormous complexity of these microbial communities. Despite the finding that biofilm structures differed among patients, streptococci dominated in the majority of patients, and the genus *Streptococcus* together with three other genera (*i.e., Actinomyces*, *Schaalia* and *Veillonella*) formed the core early microbiome. The observed dominance of *Streptococcus* spp. in health-associated early biofilms is in good agreement with the results of similar studies for implants and teeth^20,22,48,70,71,78^. The central role of the core genera in the sequential colonization of oral surfaces and the synergistic degradation of mucin is well described^1,79,80^. Bridging organisms or those that form aggregate/biofilm scaffolds were abundant in a few samples but otherwise absent (*Lactobacillus* spp. and *Corynebacterium* spp.) or were widespread but low in abundance (*Fusobacterium* spp.), contributing to a patient-specific biofilm architectures^73,74,81^. Interestingly, *Granulicatella*, *Gemella* and *Schaalia* spp. were associated with higher biofilm viability. As these organisms are rarely used in polymicrobial in vitro models^82^, their effects on biofilm development remain largely unknown and should be addressed in more detail in the future.

Besides the well-known core genera, we detected one hundred unnamed phylotypes (27% of all species-level taxa), a tenfold increase compared to a similar study for teeth^78^. This observed differences may result from the higher sensitivity and resolution of PacBio SMRT sequencing compared to semi-quantitative microarrys. Some of these taxa, e.g., Bacteroidales [G-2] sp. HMT-274, *Selenomonas* sp. HMT-126, and Saccharibacteria (TM7) [G-1] sp. HMT-349 and [G-5] sp. HMT-356 have been associated with oral disease^83^ and recent advances in culture strategies have provided a basis for future functional studies of these microorganisms^84–87^.

Potential pathogens were prevalent in our study but generally not abundant, which is consistent with previous findings on early implant-associated biofilms^25,49,59,88^. However, previous studies have often underestimated the taxonomic diversity due to methodological limitations. The inhibition of potentially pathogenic populations in the biofilms of this study may be due to the presence of genera which could be beneficial in the peri-implant space (i.e., *Bifidobacteria*, *Lactobacillus sensu lato*, *Streptococcus*), and which are thought to be involved in colonization resistance^89–91^. Even if low in abundance, *Capnocytophaga sputigena*, *Fusobacterium* sp. and *Eikenella corrodens* were the most common early potential pathogens in the early implant-associated biofilms and were positively associated with each other. These three species have been found in various oral and odontogenic infections^92,93^, are capable of producing different virulence factors^93–95^ and have been linked to uncontrolled type-2 diabetes^96^. Populations of all three species were observed in a few patients and strongly expanded in one of them, suggesting their synergistic growth. Interestingly, as this potentially pathogenic consortium was already observed before the onset of clinical symptoms, it may have the potential for the early diagnosis and subsequent therapy of dysbiosis around dental implants, by circumventing the treatment resistance of mature biofilms.

Network analysis and ecological relationship prediction showed positive correlations and stable clustering of microorganisms in early biofilms. This possibly indicates synergistic metabolic interactions between microorganisms or their preference for the same ecological niches. For example, associations observed in the largest cluster between *Aggregatibacter*, *Corynebacterium*, *Lautropia*, and *Porphyromonas* showed that members of these genera have a high potential for metabolic interactions, suggesting that they are highly adapted to, and to a certain degree dependent on, utilizing such interactions within the biofilms in the human oral cavity. This is in line with their reported simultaneous detection on teeth, even though their detection has been dependent on the clinical status^73,97–100^. In contrast, the association between *Arachnia* and Saccharibacteria within this cluster can be explained by parasite-host interactions^87^. Until now, complexes/clusters of oral microorganisms have been described for biofilms on tooth surfaces^97–101^ but not on implants.

Taxonomic architectures are hard to interpret due to uncertainty about biochemical details, metabolic functions, structural components and environmental conditions^102–104^. To address this challenge, we propose a graph database containing detailed and curated experimental information about oral microbial physiology and ecology. The obtained densely connected networks highlight collective interactions between oral taxa. Stable community-level functions were observed despite large taxonomic diversity. Observed division of labor in biofilms can be explained by the Black Queen Hypothesis describing exploitation of chemicals or enzymes^105^. Amplicon Sequence Variant (ASV) analysis utilizing PacBio circular consensus sequencing achieves single-nucleotide resolution of microbial diversity with an exceptionally low error rate^55^. In our study, this method allowed us to characterize the subspecies diversity of early biofilms, which exhibited a slightly lower Shannon index compared to mature oral biofilms^106^. Our database can be expanded in the future by incorporation of in silico reconstructions of metabolic networks^107–110^, high-order interactions^111^, subspecies taxa and patterns that transcend study systems^112^.

In conclusion, the structure and composition of early biofilms forming on dental implants are complex, patient-specific and dynamic, supporting the view that characteristics of biofilms depended on the local environment as shaped by environmental factors and an individual’s genetics^113^. Although the oral microbiota formed diverse patient-specific ecological networks, clusters of taxa showing positive associations emerged and their prevalent metabolic interactions were predicted, suggesting that some of the typical relationships between oral biofilm members also ensure survival and colonization on the implant surface. Thus, our approach of an in vivo model with two locations of modified temporal implant abutments combined with the application of state-of-the-art microscopic and taxonomic analyses provided useful insights into the microbial interactome and hypotheses for a better understanding of early biofilms.

## Methods

### Subject population

The study included 12 patients with at least two exposed implants after successful osseointegration (Fig. 1). Power analysis was performed using G*Power^114^, and based on previous data on biofilm development on titanium surfaces in the oral cavity^48^. Ethical approval was granted by the local ethics committee of Hannover Medical School (Ethic Protocol number 9477). Exclusion criteria were the presence of diseases that strongly modulate the immune system, such as diabetes mellitus, misuse of alcohol, drug or nicotine-containing products, pregnancy or lactation, and antibiotic treatment within the previous three months. After placement of the implant abutments, participants were asked to continue with their usual dental care but to avoid the use of any oral rinse solution.

### Modification of temporary implant abutments to improve biofilm analysis

A flat implant surface is required for accurate confocal microscopy of biofilms. Three implant abutments with specific flat surfaces were fabricated for each patient^23,49^. The implant abutments were attached to a disc using dental wax (Gebdi Dental, Engen, Germany) for fixation. In order to produce a flat surface of defined roughness for the investigation of biofilm formation, one side of each abutment was machined using a BUEHLER Variable Speed Grinder-Polisher (Power Prp 4000, IL, USA) with a rotary grinding disc of 40 µm grain size for 30 s (Fig. 1a). The wax was then removed manually with acetone. Finally, the implant abutments were sterilized. Tactile roughness measurement (Marsurf M400, Mahr, Göttingen, Germany) of titanium surfaces determined the surface parameters *R*_a_ = 0.3 µm, *R*_z_ = 2.6 µm and *R*_max_ = 3.3 µm. Their contact angle measured by sessile drop method (OCA 40, DataPhysics Instrumtents GmbH, Filderstadt, Germany) was ~70°.

### Placement and removal of temporary implant abutments

In order to study biofilm formation over a period of one, two and three weeks, for each patient two implant sites were selected, referred to as “Location 1” and “Location 2”. After a healing period of fourteen days following implant exposure, modified implant abutments were mounted to these implants (Fig. 1b, visit 1). The abutment at Location 1 was replaced after one week (visit 2, one-week biofilm) and again after an additional two weeks (visit 3, two-week biofilm). The abutment at Location 2 remained in place for three weeks, and biofilm samples were also collected during visit 3 (three-week biofilm).

The biofilm formed on the examination surface of each abutment was analyzed by confocal laser scanning microscopy (CLSM) within four hours after removing the abutment. The biofilm material was subsequently stored at −20 °C until DNA isolation and sequencing of the full-length 16S rRNA gene amplicons.

### Clinical examination

All clinical examinations were performed by a trained dentist using a calibrated method (Fig. 1c, d). Clinical measurements were recorded on three occasions: placement of implant abutments (visit 1), replacement of implant abutments (visit 2 and visit 3). Measurements were taken at six sites of either the implant or the tooth, i.e., mesiobuccal, buccal, distobuccal, mesiolingual/-palatinal, lingual/palatinal and distolingual/-palatinal. Clinical measurements included: (i) Periodontal Screening Index (PSI), with “0” = no bleeding, no plaque/calculus, Probing Pocket Depth (PPD) < 3.5 mm, “1” = bleeding on probing, no plaque/calculus, PPD < 3.5 mm, “2” = calculus or defective margins, PPD < 3.5 mm, “3” = PPD 3.5 mm–5.5 mm, and “4” = PPD > 5.5 mm; (ii) modified Mucosal Index (mGI) similar to the modified Gingival Index^115^, with “0” = normal gingiva; “1” = slight inflammation, little change in color, small oedematous swelling, no bleeding on probing; “2” = moderate inflammation, redness, oedematous swelling, bleeding on probing; “3” = clear inflammation, clear redness, clear oedematous swelling, ulceration, tendency to spontaneous bleeding; (iii) modified Plaque Index (mPI)^26^, with “0” = no plaque; “1” = plaque only visible while probing; “2” = plaque visible to the eye; “3” = extensive plaque visible; (iv) Bleeding on Probing (BOP), defined as either “no” or “yes” (or converted to “0” and “1”); (v) Probing Pocket Depth (PPD), measured in millimeters using the PCPUNC15 (HuFriedy, Frankfurt am Main, Germany); and (vi) Mucositis Severity Score (mGi-BOP)^116^. mGi-BOP is a surrogate variable used to define the severity of peri-implant mucositis on a scale of zero to twenty-four, consisting of the sum of the 6 individual BOP and mGI measurements. Finally, digital intraoral radiographs of each implant were taken to access the marginal bone level.

### Biofilm examination with confocal laser scanning microscopy (CLSM)

After removal of the abutment, biofilms were stained with the LIVE/DEAD BacLight Bacterial Viability Kit (Invitrogen), rinsed, fixed in 2.5% glutaraldehyde solution in phosphate buffered saline, and examined using CLSM (Leica TCS SP8, Leica Microsystems, Mannheim, Germany) as previously described in the studies of Doll et al.^66^ and Desch et al.^48^. In summary, a 488 nm laser was used with an emission range of 500–545 nm for SYTO9 and a 552 nm laser was used with emission range 590–680 nm for propidium iodide (PI). Five representative image stacks were acquired for each examination surface (Supplementary Fig. 1a), with optical sections of 5 µm. Each stack represented a square area with a side length of 800 µm. Upper areas 1 and 2 were usually located supramucosally; middle areas 3 and 4 were intermediate, while lower areas 5 were usually located submucosally (Figs. 1b and 2). These five analyzed implant areas corresponded to approximately a quarter of the entire examination surface. The Imaris Cell Imaging software package (Imaris x64, 6.2.1, Bitplane AG, Zürich, Switzerland) was used to analyze the images. 3D reconstructions were generated. Biofilm volumes were calculated using the surface wizard setting. Green (SYTO9), red (PI) and yellow (co-localized SYTO9 and PI) fractions were determined (Supplementary Fig. 1b). Volumes were considered to include non-permeable viable cells if green but not red signals were detected. Parameters describing the number and mean size of microcolonies as well as the total area covered and the percentage of area covered were calculated using ImageJ software v1.48 (Wayne Rasband, National Institute of Health, MD, USA). Seven parameters, i.e., biofilm volume, non-permeable cell volume, permeable cell volume, percent of permeable cell volume, number of microcolonies, mean size of microcolonies, and area covered by biofilm were included in further analyses while two redundant parameters were excluded (Supplementary Fig. 1b).

### Microbiological assessment by high-throughput sequencing of full-length 16S rRNA gene amplicons

The composition of the biofilms was studied by 16S rRNA gene amplicons sequencing for twenty-four biofilms, eight for each time point. After CLSM, DNA was purified from biofilms using the Fast DNA Spin Kit for Soil (MP Biomedicals Germany GmBH, Eschwege, Germany). DNA quantification was then performed using the Invitrogen Quibit dsDNA and BR Assay Kit (Invitrogen, Waltham, MA, USA) and the Qubit 2.0 fluorometer (Thermo Fisher Scientific, Waltham, Massachusetts, USA). Afterwards, full-length 16S rRNA gene amplicons were generated. Each PCR mix had a volume of 50 µl and consisted of DNA template, adjusted to 5 ng whenever possible, KAPA PCR mix, forward and reverse primers (27F – AGRGTTYGATYMTGGCTCAG and 1492R – RGYTACCTTGTTACGACTT) and molecular grade water. PCR was performed on a Biometra Thermocycler TProfessional (Biometra, Göttingen, Germany). The target region was amplified in a single PCR step, with the number of cycles adjusted for each sample depending on the amount of DNA present. After initial denaturation for 3 min at 95 °C, 23 – 30 cycles were performed, each consisting of denaturation for 30 s at 95 °C, annealing for 30 s at 55 °C, and synthesis for 90 s at 72 °C. The cycles were followed by a final synthesis for 10 min at 72 °C. Agarose gel electrophoresis of the 5 µl PCR reaction was used for quality control. PCR products were purified on the same day using the MiniElute PCR Purification Kit 250 (Qiagen, Hilden, Germany) and the DNA yield was measured using Qubit dsDNA HS Assay-Kit (Thermo Fisher Scientific, Waltham, Massachusetts, USA). Purified PCR products were used for PacBio Sequel Sequencing. Biomass was insufficient to generate amplicons in individual samples from two patients. PacBio CCS reads were filtered to a minimum quality of Q30 within PacBio SMRT Link 10.1 and exported as fasta sequences. Further processing and taxonomic classification was based on the in-house pipeline^48^. Samples were demultiplexed and primers were trimmed using tagcleaner standalone 0.16^117^. Reads with more than one mismatch in a barcode sequence, more than five mismatches in a primer sequence, or a trimmed length outside the expected range of 1000 to 2100 bp were discarded. Reads were classified to species if unequivocal matching species classifications was obtained by BLAST searches (standalone BLAST+ v. 2.5.0) against SILVA + HOMD and LTP + HOMD databases. SILVA + HOMD consisted of the bacterial 16S sequences annotated with species names within SILVA SSURef NR99 version 132^118^ supplemented with data from the HOMD 16S sequence database version 15.1^119^. LTP + HOMD encompassed the living tree project database LTPs 132, which contains the 16S sequences of prokaryotic type strains, as well as the HOMD 16S sequences and provisional taxon IDs of newly identified species not yet associated with type strains and species names. Sequences that were not identified to species were clustered into 97% identity OTUs using the uparse algorithm implemented in the usearch version 10.0.240^120^. Representative sequences of the OTUs and unique sequences with species identification were additionally classified to genus level and above using the rdp classifier version 2.13^121^. The dataset was screened for mitochondrial or plant plastid sequences. Sequences that could not be classified at least to class level were only retained if the SILVA NR99 database contained at least 50 sequences with at least 95% identity over 399 or more nucleotides. For illustration purposes, sequence numbers were converted to approximate bacterial cell numbers based on the Ribosomal RNA database rrnDB-5.6^122^. Sequencing of 30 samples yielded a total of half a million reads representing the full-length 16S rRNA gene sequence. Reads corresponding to typical reagent impurities were identified using correlation analysis^123^ and removed. A single Operational Taxonomic Unit (OTU) classified as *Pseudomonas* sp. was the main contaminant, accounting for 97% of all contaminating reads. After identification and removal of contaminants we selected twenty-four samples for inclusion in the final set based on a sequencing depth cut-off of 1000 reads per sample. Mean sequencing depth was 8725 ± 4441 (s.d.). 212,006 reads were grouped into 371 species-level taxa, 86 genera, 53 families, 31 orders, and 17 classes.

Additional amplicon sequence variant (ASV) analysis was performed based on fastq sequences with a minimum quality of Q30 originating from the same raw sequencing data. Sequences were demultiplexed and primers were removed with the tagcleaner program as detailed above. Using the R package dada2 (v. 3.13)^54^, sequences were preprocessed with the filterAndTrim options “minQ = 3, minLen = 1000, maxLen = 2100, maxN = 0, rm.phix = FALSE, maxEE = 2” and dereplicated. Errors were learned with the settings “errorEstimationFunction = PacBioErrfun, randomize = TRUE, BAND_SIZE = 32, multithread=TRUE”, and ASVs were inferred using the pseudo-pooling option. Where possible, ASVs were classified at the species level using the BLAST-based method detailed above. Higher-level classifications were based on the rdp classifier with a minimum accepted bootstrap score of 80%. ASV sequences without species identification were additionally mapped to the previously calculated OTUs using the usearch setting “usearch_global –id 97”. The final ASV list was manually curated by removing ASVs if the rdp output classified them as chloroplast, eukaryotic or archael, if they were not classified at the class level and not mapped to an OTU if they mapped to an OTU automatically discarded by pipeline detailed above, or if their species or OTU was previously identified as a contaminant. To further exclude potential contaminants, ASVs not mapping to a species or an OTU were also discarded. Sequencing data is available at European Nucleotide Archive (PRJEB71108).

### Similarity of species and inferred interspecies interactions in early biofilms

Similarity of species ‘abundance’ between samples was compared using SIMPROF routines with or without clustering based on Whittaker’s index of association. Minor species (less than 1% in any sample) were omitted from the analysis because their effect on calculated similarities is small, but they increase the amount of ‘noise’ in the subsequent displays. Tests of type 2 and 3 were applied with 100,000 permutations. The histogram of π distance values relative to the observed π, indicates the statistical significance of the observed difference—that is, a deviation of the real profile from the group of simulated profiles. Stability of the groupings were evaluated across the broad range of levels. Higher levels can be considered unadjusted for multiple testing, while lower levels may be seen as potentially overprecise. Analyses were performed at the species-level taxa, genus and class levels. Group mean mode was used for clustering.

The potential of taxa to engage in interspecies interactions was inferred for the most abundant species (max relative abundance >2%) and genera (top 50 with the highest mean relative abundance) using a custom database (Supplementary File 1). This is part of the Database for Oral Microbial Interactions Networks (DOMINO), which we designed to integrate and link multiple types of data, including species, enzymes, metabolites, interactions and references. We have implemented the database using the Neo4j platform, with nodes hyperlinked to other databases such as eHOMD, HMDB, KEGG, and PubMed. Microbial interaction information has been sourced from the literature (e.g., Bergey’s Manual of Systematic Bacteriology^124^), followed by manual curation. Custom-made graphs were used to visualize networks.

### Statistical analyses

Most analyses were performed using either PRIMER, version 7, or PERMANOVA+ (an add-on package that extends the methods of PRIMER)^125,126^, or IBM SPSS version 27. Scatter plots for variable pairs were generated using the Draftsman Plot PRIMER routine on untransformed and transformed data for diagnostic purposes prior to further analysis. Non-metric multidimensional scaling (nm-MDS) was performed using the PCO PERMANOVA+ routine based on the Euclidean distance matrix, for variables describing biofilm structure, or the Bray–Curtis similarity matrix for variables describing biofilm composition. For the biofilm structure, the input data was first standardized to the maximum of the corresponding variables and then transformed to the square root. Ordination was also performed for mean profiles representing patient-time combinations. For the biofilm composition, input data was first standardized to the sum of reads in each sample and then various aggregation and transformation operations were applied. Reads were aggregated to genus and class. Taxon abundances were transformed by fourth root. A vector overlay was used to visualize correlations between the biofilm structure or clinical information and the ordination axes. Each vector begins at the center of a circle (0, 0) and ends at the coordinates (*x*, *y*) consisting of the Pearson correlation coefficient between that variable and each of the ordination axes 1 and 2, respectively. The length and direction of the vector indicate the strength and direction of the relationship between the variable and the ordination axes, respectively. Group-averaged agglomerative hierarchical clustering was performed on the basis of the Bray–Curtis matrix quantifying the pairwise similarities in biofilm composition. Permutational MANOVA (PERMANOVA) was used to test the simultaneous response of Euclidian distance of biofilm phenotypes to one or more factors describing implants (e.g., patient, time, location, clinical measures) in an analysis of variance (ANOVA) experimental design, using permutation methods. In some cases, *a posteriori* pair-wise comparisons among levels of factors were performed. The Shannon diversity index H’ was calculated using the DIVERSE routine on rarefied abundances for species-level taxa.

## Supplementary information

Supplemental material

## Data Availability

The raw sequencing data supporting the findings of this study are be deposited in European Nucleotide Archive (PRJEB71108).
