# Supplementary material for: Structure and composition of early biofilms formed on dental implants are complex, diverse, subject-specific and dynamic
Source: NPJ Biofilms Microbiomes. 2024 Dec 24;10:155. doi: 10.1038/s41522-024-00624-3 (PMC11668855; doi:10.1038/s41522-024-00624-3)
Supplement: Supplementary file 1 — Supplemental material [file 41522_2024_624_MOESM1_ESM.pdf]

Supplementary Information for "Structure and composition of early biofilms formed on dental implants are complex, diverse, subject-specific and dynamic"

Short title: Biofilms on implants: supplementary information

Sophie Dieckow[1,*], Szymon P. Szafrański[1,2,3,*], Jasmin Grischke[1], Taoran Qu[1,2], Katharina Doll-Nikutta[1,2], Matthias Steglich[1,2], Ines Yang[1,2], Susanne Häussler[3,4,5,6], and Meike Stiesch[1,2,3,$]

[1]Department of Prosthetic Dentistry and Biomedical Materials Science, Hannover Medical School, Hannover, Germany

[2]Lower Saxony Centre for Biomedical Engineering, Implant Research and Development (NIFE), Hannover, Germany

[3]Cluster of Excellence RESIST (EXC 2155), Hannover Medical School, Hannover, Germany

[4]Department of Molecular Bacteriology, Helmholtz Centre for Infection Research, Braunschweig, Germany

[5]Institute for Molecular Bacteriology, Twincore, Centre for Clinical and Experimental Infection Research, Hannover, Germany.

[6]Department of Clinical Microbiology, Copenhagen University Hospital - Rigshospitalet, Copenhagen, Denmark

[*]Sophie Dieckow and Szymon P. Szafrański contributed equally

[$]correspondence to: Prof. Dr. Meike Stiesch, Department of Prosthetic Dentistry and Biomedical Materials Science, Hannover Medical School Carl-Neuberg-Str.1 30625 Hannover, Germany; stiesch.meike@mh-hannover.de

Table of contents:

Supplementary Fig. 1 Confocal images of biofilms formed on implant abutments and biofilm parameters. 4

Supplementary Fig. 2 Features of biofilm structures 5

Supplementary Fig. 3 Composition of biofilms at genus level, their dynamics and their relationships with biofilm parameters 6

Supplementary Fig. 4 Relationship between bacterial relative abundances at three taxonomic levels. 7

Supplementary Fig. 5 Interspecies interaction networks predicted in biofilms. 9

Supplementary Fig. 6 ASV diversity. 11

Supplementary File 1. List of microbe-metabolite/enzyme interactions predicted in early biofilms 12

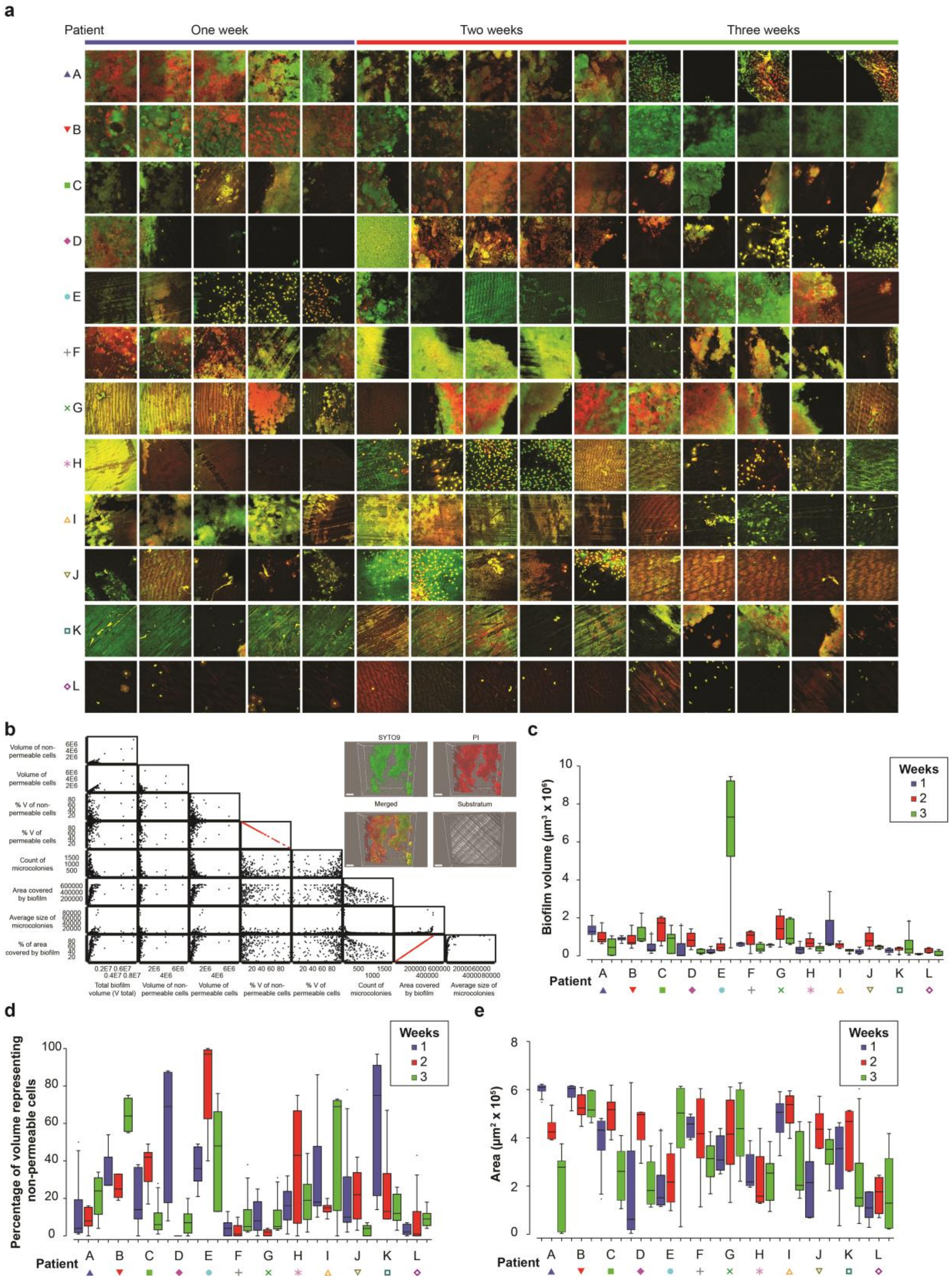
a
Patient
One week
Two weeks
Three weeks
A
B
C
D
E
F
G
H
I
J
K
L
b
c
Weeks
Biofilm volume (µm³ x 10⁶)
Patient
d
Percentage of volume representing non-permeable cells
e
Area (µm² x 10⁵)

33

Supplementary Fig. 1 Confocal images of biofilms formed on implant abutments and biofilm parameters.

a CLSM micrographs. Images, which represent maximum intensity projections, are sorted by patients (different rows), age of biofilms in weeks (three sets of columns) and area numbers. Images number 1 to 5, representing all replicates mapped Fig. 1b, are shown from left to right for each week. Each image has a size of 800 µm by 800 µm. In some cases human cells and no or little microbial biofilms were observed. **b** Relationship between biofilm parameters. Scatter plots for pairs of measurements. Highly correlated measurements are indicated in red. Representative micrographs of biofilm shown. The percentage of volume representing non-permeable "live" cells was calculated as the volume of all cells minus the volume of permeable "dead" cells. The bar represents 100 µm. **c – d** Biofilm parameters were calculated as an average value for abutments (five CLSM images each) Box plots (n = 5) show all twelve patients indicated by symbols and the three time points indicated by box colors. **c** Biofilm volume**. d** Percent of volume representing non-permeable "live" cells. **e** Area covered by biofilm.

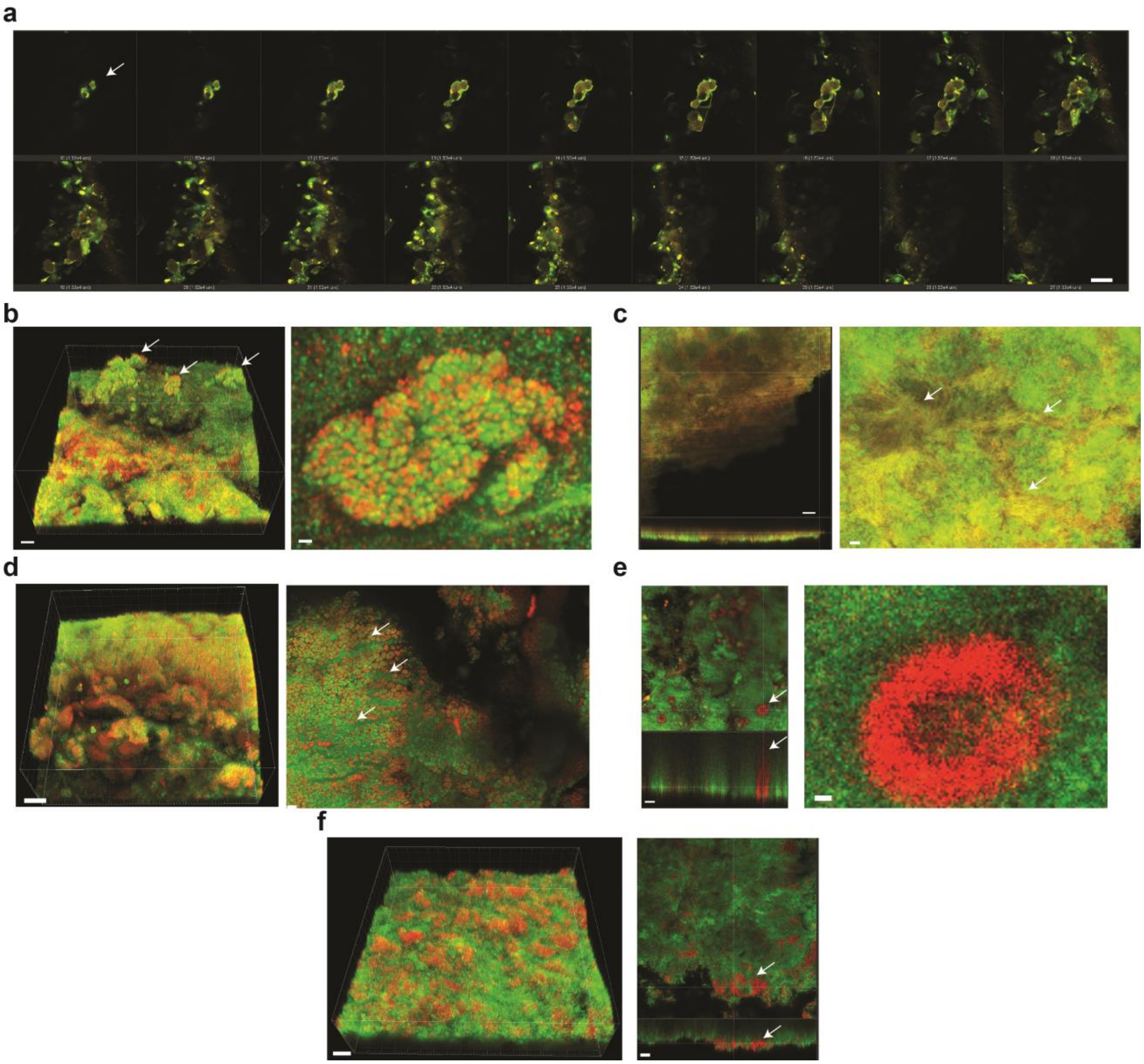

Supplementary Fig. 2 Features of biofilm structures

a Biofilm from patient J formed by epithelial cells. Biofilm slices are shown. Elevated central surface with steep slope is indicated with arrow. **b** Biofilm from patient K characterized by aggregates forming vertical projections. Projections are indicated with arrow in 3D biofilm re-construction (left). Example aggregate was enlarged (right). **c** Biofilm from patient F characterized by extensive scaffolding. Scaffolding cells were located at the bottom (left). Cell bundles forming scaffolds were enlarged (right). **d** Biofilm from patient C characterized by aggregates formed by permeable cells. 3D biofilm re-construction (left). Channels between aggregates filled by distinct population of non-permeable cells were enlarged (right). **e** Biofilm from patient E characterized by the presence of chimneys. Chimney-like structures protruded through the entire depth of biofilm (left). Section through chimney-like structure was enlarged (right). **f** Biofilm from patient B characterized by mosaic of cell clusters. 3D biofilm re-construction (left). Upper location of aggregates formed by permeable cells is indicated with arrow (right).

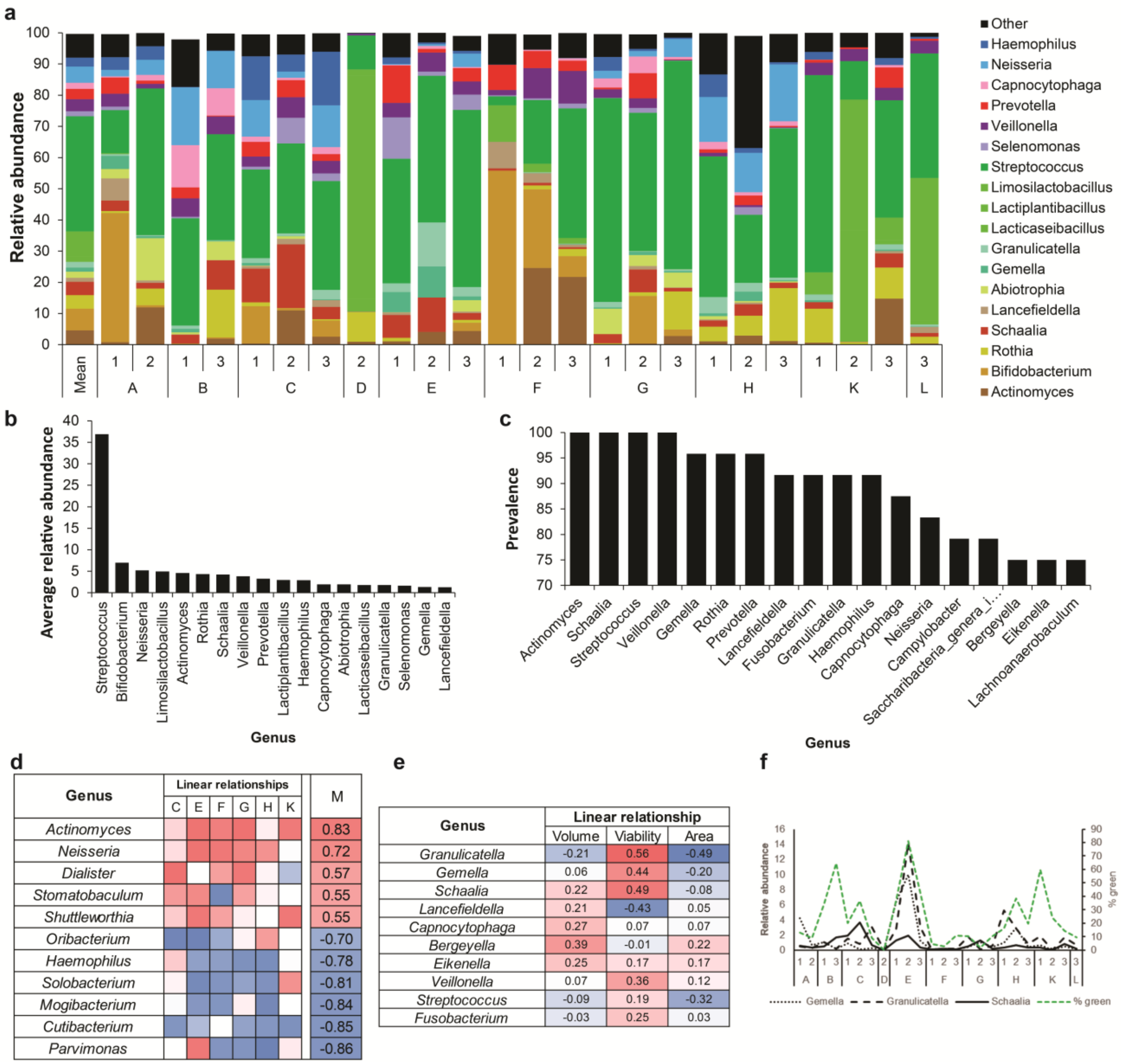

d

Genus	Linear relationships (C, E, F, G, H, K)	M
Actinomyces		0.83
Neisseria		0.72
Dialister		0.57
Stomatobaculum		0.55
Shuttleworthia		0.55
Oribacterium		-0.70
Haemophilus		-0.78
Solobacterium		-0.81
Mogibacterium		-0.84
Cutibacterium		-0.85
Parvimonas		-0.86

e

Genus	Linear relationship		
	Volume	Viability	Area
Granulicatella	-0.21	0.56	-0.49
Gemella	0.06	0.44	-0.20
Schaalia	0.22	0.49	-0.08
Lancefieldella	0.21	-0.43	0.05
Capnocytophaga	0.27	0.07	0.07
Bergeyella	0.39	-0.01	0.22
Eikenella	0.25	0.17	0.17
Veillonella	0.07	0.36	0.12
Streptococcus	-0.09	0.19	-0.32
Fusobacterium	-0.03	0.25	0.03

Supplementary Fig. 3 Composition of biofilms at genus level, their dynamics and their relationships with biofilm parameters

a Relative abundance of reads grouped at the genus level and plotted for each patient-time point combination as well as for average profile. The most abundant genera are shown, while the reads matching other genera were summed up and plotted together as “Other”. **b** Most abundant genera. **c** Most prevalent genera. **d** Pearson’s correlation values between time and abundances of selected genera. Heat map shows correlation values for the abundances of the genera in each patient. Genera are sorted by decreasing median values across patients from top to bottom. Median values are plotted next to the heatmap. **e** Correlations between parameters describing biofilm structures and relative abundances for selected genera. Only genera showing absolute values of correlation higher than 0.3 with at least one parameter are shown. **f** Coherent variable curves. Line plots of relative abundances (for *Gemella*, *Granulicatella*, and *Schaalia*) and for % of volumes filled by non-permeable cells in biofilm reflecting viability.

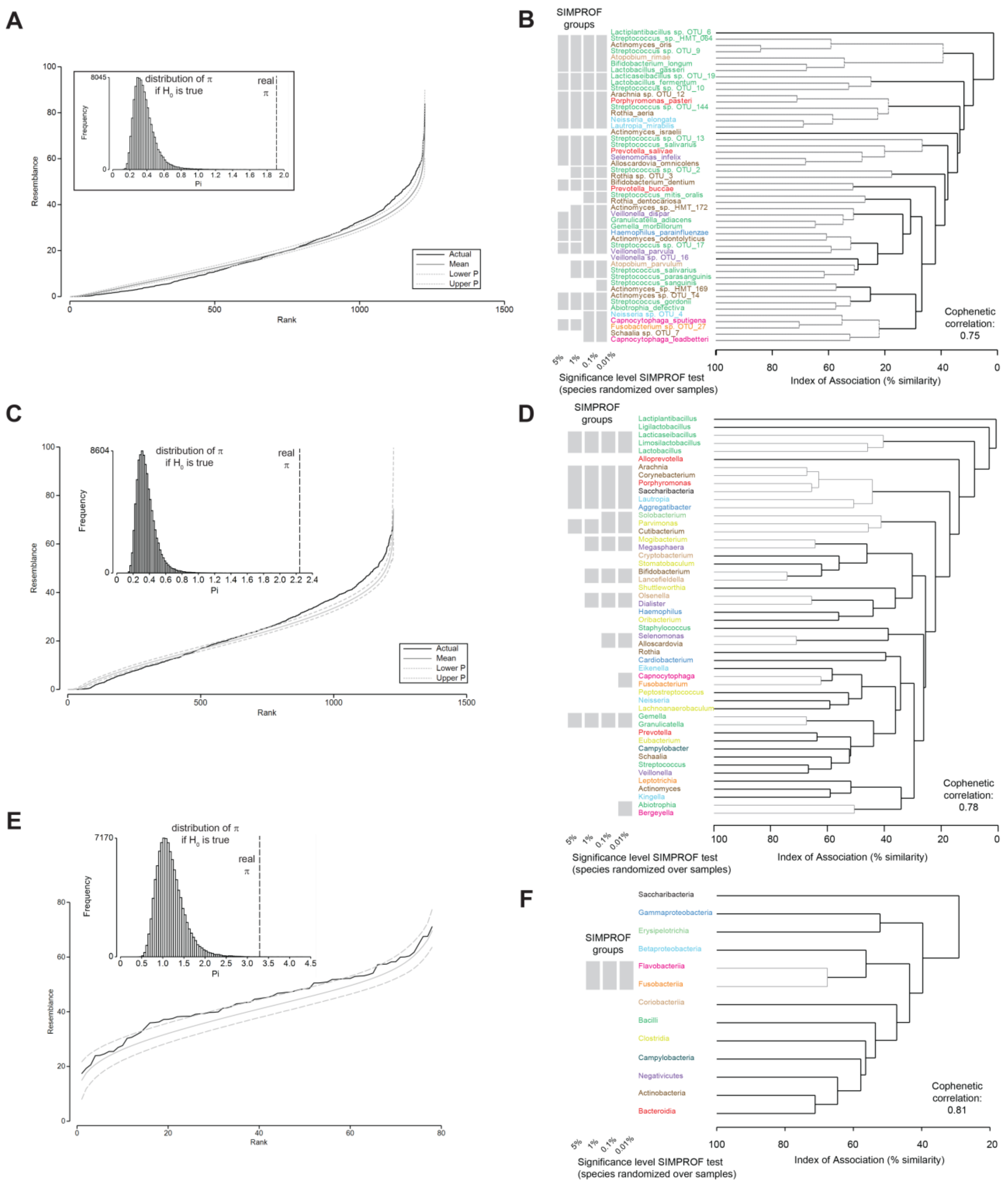

Supplementary Fig. 4 Relationship between bacterial relative abundances at three taxonomic levels.

R-mode analysis using pairwise Whittaker's associations was performed for taxa at three different taxonomic levels. **A** Similarity profile for species. **B** Dendrogram for species. **C** Similarity profile for genera. **D** Dendrogram for genera. **E** Similarity profile for classes. **F** Dendrogram for classes. Type 2 SIMPROF tests were performed to validate the hypothesis that there is no associations of any sort among taxa. Values were permuted 100,000 times for each taxon

across all samples. Type 3 SIMPROF tests were performed for grouping of taxa detected with type 2 test to validate the hypothesis that the associations within these subsets are not distinguishable, i.e. that the taxa are coherent in their patterns of abundance across the full sample set. This time values orthogonal permutation scheme was applied (n = 100,000), namely taxa we permuted across the subset of taxa. For each taxonomic levels similarity profile with inset visualizing distribution of the distance π (left), and dendrogram (agglomerative, group average linked) from an index of association matrix among taxa (right) are shown. Similarity profiles were obtained by computing the indices of association, ordering them (y axis) and plotting against their ranks (x axis) to generate continuous black line. Also shown, for each value of x, is the mean index (continuous grey line) from 100,000 permutations of the data matrix (under the null hypothesis), and the range (dashed grey lines) in which 99% of the permuted index values lie. In inset the distribution of the distance π of (a further) 100,000 permuted profiles from the mean profile, in comparison with π for the real profile (seen not to come from the null, establishing the existence of taxon associations). In dendograms grey lines and grey boxes denote the coherent groups of taxa containing more than one taxon. Broad levels of SIMPROF tests (5%, 1%, 0.1%, and 0.01%) were compared to discover stable relationships.

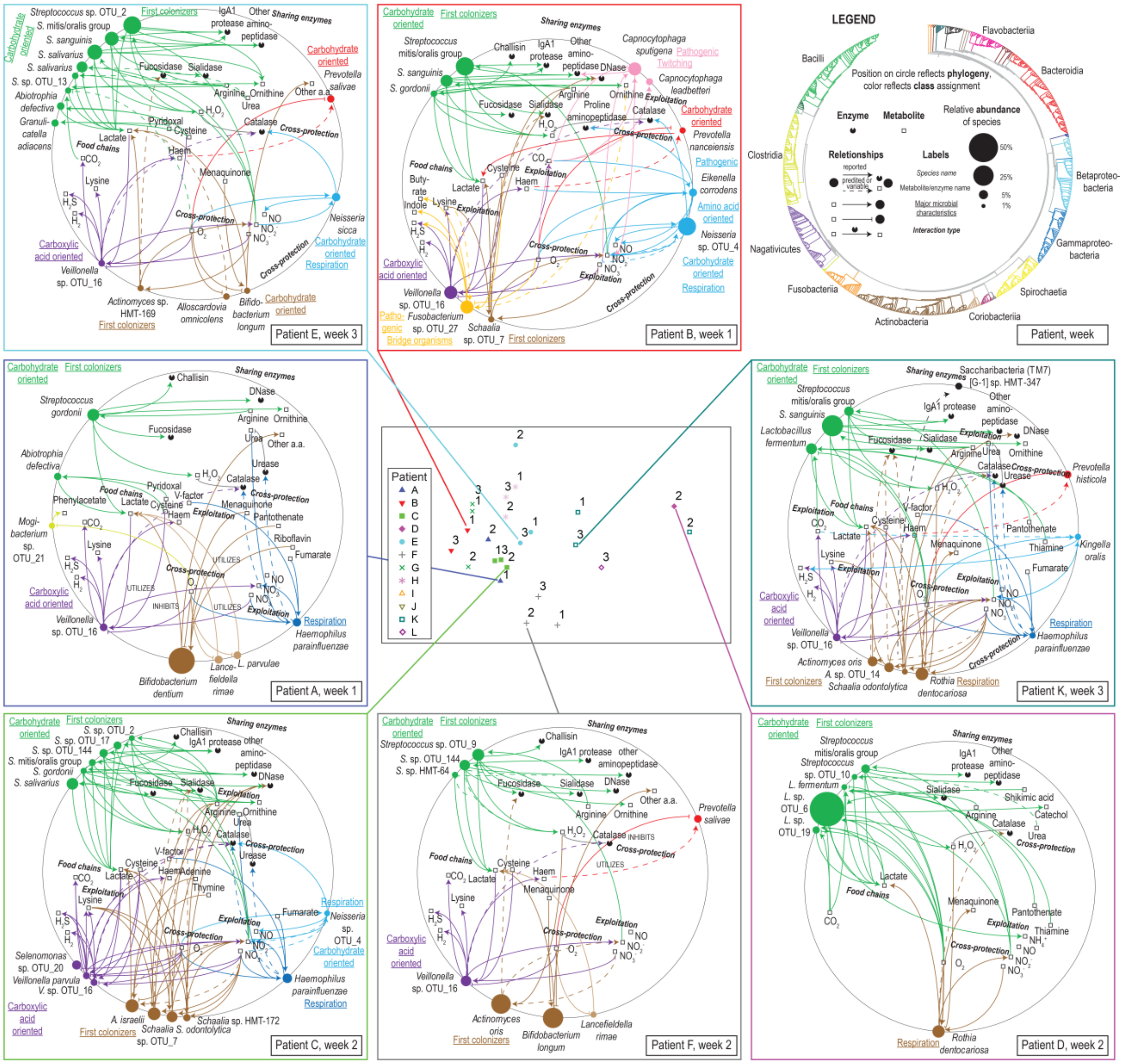

Supplementary Fig. 5 Interspecies interaction networks predicted in biofilms.

Ecological interactions were inferred using a custom-made database. Briefly, for all taxa that reached at least 2% of relative abundance, information on interspecies interactions were retrieved from the literature, transformed into a graph database and visualized using custom-made circular graphs. Ecological profiles are placed in similar orientation as in Fig. 3, except that the missing ecological profile for patient J was replaced with a comprehensive figure legend. The ordination in the middle was constructed using the relative abundance of reads grouped to species.

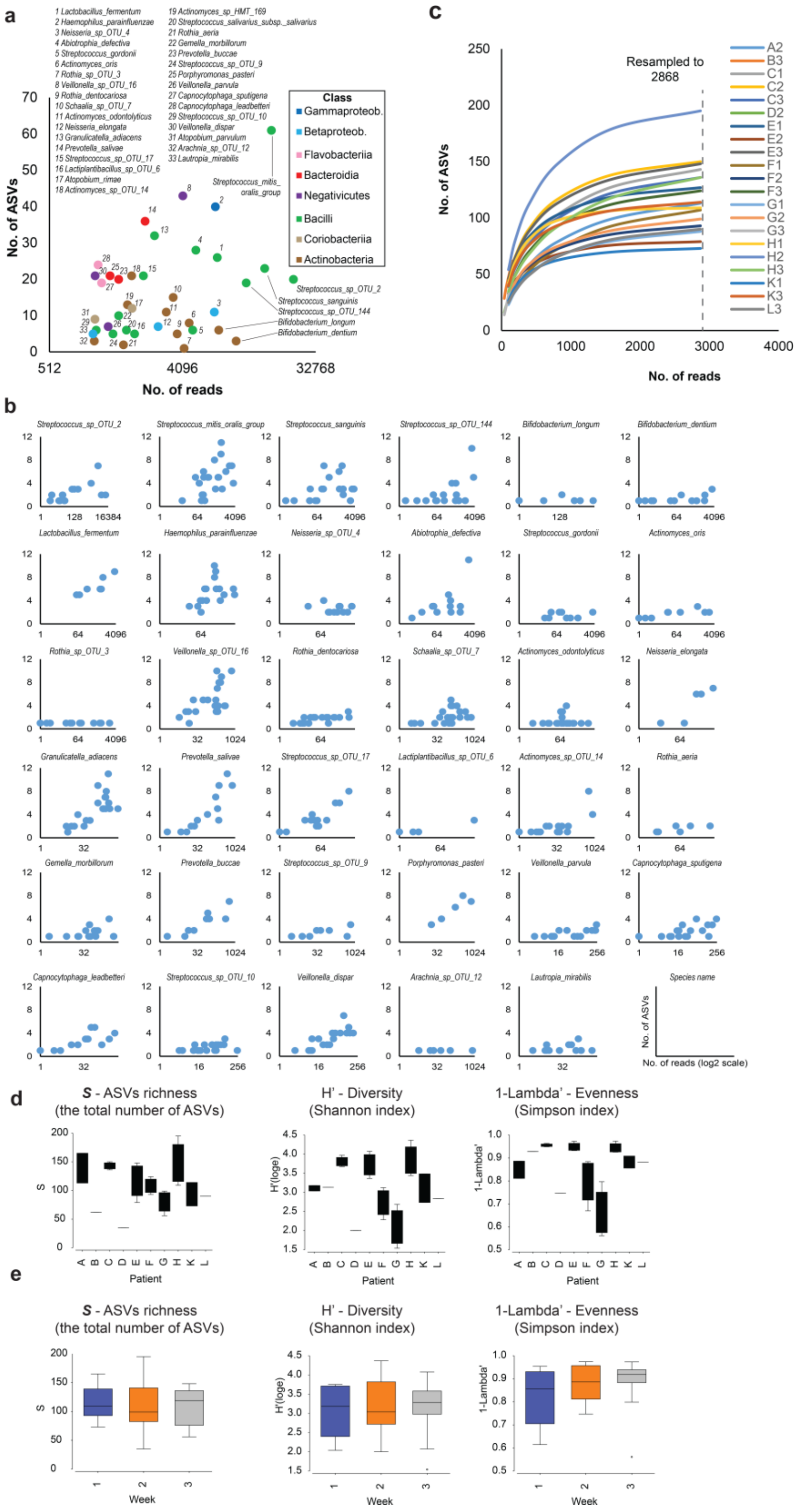
a
1 Lactobacillus_fermentum
2 Haemophilus_parainfluenzae
3 Neisseria_sp_OTU_4
4 Abiotrophia_defectiva
5 Streptococcus_gordonii
6 Actinomyces_oris
7 Rothia_sp_OTU_3
8 Veillonella_sp_OTU_16
9 Rothia_dentocariosa
10 Schaalia_sp_OTU_7
11 Actinomyces_odontolyticus
12 Neisseria_elongata
13 Granulicatella_adiacens
14 Prevotella_salivae
15 Streptococcus_sp_OTU_17
16 Lactiplantibacillus_sp_OTU_6
17 Atopobium_rimae
18 Actinomyces_sp_OTU_14
19 Actinomyces_sp_HMT_169
20 Streptococcus_salivarius_subsp._salivarius
21 Rothia_aeria
22 Gemella_morbillorum
23 Prevotella_buccae
24 Streptococcus_sp_OTU_9
25 Porphyromonas_pasteri
26 Veillonella_parvula
27 Capnocytophaga_sputigena
28 Capnocytophaga_leadbetteri
29 Streptococcus_sp_OTU_10
30 Veillonella_dispar
31 Atopobium_parvulum
32 Arachnia_sp_OTU_12
33 Lautropia_mirabilis
Class
Gammaproteob.
Betaproteob.
Flavobacteriia
Bacteroidia
Negativicutes
Bacilli
Coriobacteriia
Actinobacteria
Streptococcus_mitis_oralis_group
Streptococcus_sp_OTU_2
Streptococcus_sanguinis
Streptococcus_sp_OTU_144
Bifidobacterium_longum
Bifidobacterium_dentium
No. of ASVs
No. of reads
c
Resampled to 2868
A2
B3
C1
C2
C3
D2
E1
E2
E3
F1
F2
F3
G1
G2
G3
H1
H2
H3
K1
K3
L3
No. of ASVs
No. of reads
b
Streptococcus_sp_OTU_2
Streptococcus_mitis_oralis_group
Streptococcus_sanguinis
Streptococcus_sp_OTU_144
Bifidobacterium_longum
Bifidobacterium_dentium
Lactobacillus_fermentum
Haemophilus_parainfluenzae
Neisseria_sp_OTU_4
Abiotrophia_defectiva
Streptococcus_gordonii
Actinomyces_oris
Rothia_sp_OTU_3
Veillonella_sp_OTU_16
Rothia_dentocariosa
Schaalia_sp_OTU_7
Actinomyces_odontolyticus
Neisseria_elongata
Granulicatella_adiacens
Prevotella_salivae
Streptococcus_sp_OTU_17
Lactiplantibacillus_sp_OTU_6
Actinomyces_sp_OTU_14
Rothia_aeria
Gemella_morbillorum
Prevotella_buccae
Streptococcus_sp_OTU_9
Porphyromonas_pasteri
Veillonella_parvula
Capnocytophaga_sputigena
Capnocytophaga_leadbetteri
Streptococcus_sp_OTU_10
Veillonella_dispar
Arachnia_sp_OTU_12
Lautropia_mirabilis
Species name
No. of ASVs
No. of reads (log2 scale)
d
S - ASVs richness (the total number of ASVs)
H' - Diversity (Shannon index)
1-Lambda' - Evenness (Simpson index)
S
H'(loge)
1-Lambda'
Patient
e
S - ASVs richness (the total number of ASVs)
H' - Diversity (Shannon index)
1-Lambda' - Evenness (Simpson index)
S
H'(loge)
1-Lambda'
Week

Supplementary Fig. 6 ASV diversity.

a ASVs detected for the most abundant species in the studied population, plotted as a function of sequencing depth for each species. Species are colored by class. **b** ASVs detected per patient for the most abundant species, also shown as a function of sequencing depth. **c** Re-sampled data showing ASV distributions. **d** ASV diversity across different patients. **e** ASV diversity across various time points.

Supplementary File 1. List of microbe-metabolite/enzyme interactions predicted in early biofilms

The rows represent relationships between taxa and other metabolites, enzymes or biofilm structures. For each relationship, the object type, supporting evidence, comments, references, links to other databases and corresponding visualization (either Fig. 7c or Supplementary Figure 5) are provided.

TAXON	HMT	eHOMD	RELATIONSHIP	OBJECT	OBJECT_TYPE	HMDB_ID	HMDB	EC	KEGG	EVIDENCE	CURATORS_COMMENTS	REFERENCES	PubMed1	PubMed2	Figure
Abiotrophia defectiva	389	https://www.homd.org/taxa/tax_description?otid=389	PRODUCES	lactic acid	metabolite	HMDB0000190	https://hmdb.ca/metabolites/HMDB0000190	n/a	n/a	experimental	Data retrived from review and not original research article.	Bergey's Manual of Systematic Bacteriology, Vol. 3-5, Edn. 2nd.	n/a	n/a	S4
Abiotrophia defectiva	389	https://www.homd.org/taxa/tax_description?otid=389	USES	L-cysteine	metabolite	HMDB0000574	https://hmdb.ca/metabolites/HMDB0000574	n/a	n/a	experimental	Data retrived from review and not original research article.	Bergey's Manual of Systematic Bacteriology, Vol. 3-5, Edn. 2nd.	n/a	n/a	S4
Abiotrophia defectiva	389	https://www.homd.org/taxa/tax_description?otid=389	USES	pyridoxal	metabolite	HMDB0001545	https://hmdb.ca/metabolites/HMDB0001545	n/a	n/a	experimental	Data retrived from review and not original research article.	Bergey's Manual of Systematic Bacteriology, Vol. 3-5, Edn. 2nd.	n/a	n/a	S4
Actinomaces sp. OTU_14	n/a	n/a	PRODUCES	fucosidase	enzyme	n/a	n/a	n/a	n/a	prediction	Prediction based on the characteristics of related A. naselundii. Data retrived from review and not original research article. Some.	Bergey's Manual of Systematic Bacteriology, Vol. 3-5, Edn. 2nd.	n/a	n/a	S4
Actinomaces sp. OTU_14	n/a	n/a	PRODUCES	lactic acid	metabolite	HMDB0000190	https://hmdb.ca/metabolites/HMDB0000190	n/a	n/a	prediction	Prediction based on the characteristics of related A. naselundii. Data retrived from review and not original research article.	Bergey's Manual of Systematic Bacteriology, Vol. 3-5, Edn. 2nd.	n/a	n/a	S4
Actinomaces sp. OTU_14	n/a	n/a	PRODUCES	menaquinone	metabolite	n/a	n/a	n/a	n/a	prediction	Prediction based on the characteristics of Actinomyces genus.Tetrahydrogenated menaquinones with ten isoprene units [MK-10(H4)] are the major respiratory quinones.	Fernandez1987 10.1111/j.1574-6968.1987.tb02191.x	n/a	n/a	
Actinomaces sp. OTU_14	n/a	n/a	PRODUCES	nitrite	metabolite	HMDB0002786	https://hmdb.ca/metabolites/HMDB0002786	n/a	n/a	prediction	Prediction based on the characteristics of related A. naselundii. Data retrived from review and not original research article.	Bergey's Manual of Systematic Bacteriology, Vol. 3-5, Edn. 2nd.	n/a	n/a	S4
Actinomaces sp. OTU_14	n/a	n/a	USES	nitrate	metabolite	HMDB0002878	https://hmdb.ca/metabolites/HMDB0002878	n/a	n/a	prediction	Prediction based on the characteristics of related A. naselundii. Data retrived from review and not original research article.	Bergey's Manual of Systematic Bacteriology, Vol. 3-5, Edn. 2nd.	n/a	n/a	S4
Actinomyces israelii	645	https://www.homd.org/taxa/tax_description?otid=645	IS_INHIBITED_BY	oxygen	metabolite	HMDB0001377	https://hmdb.ca/metabolites/HMDB0001377	n/a	n/a	experimental	Data retrived from review and not original research article.	Bergey's Manual of Systematic Bacteriology, Vol. 3-5, Edn. 2nd.	n/a	n/a	S4
Actinomyces israelii	645	https://www.homd.org/taxa/tax_description?otid=645	PRODUCES	lactic acid	metabolite	HMDB0000190	https://hmdb.ca/metabolites/HMDB0000190	n/a	n/a	experimental	Data retrived from review and not original research article.	Bergey's Manual of Systematic Bacteriology, Vol. 3-5, Edn. 2nd.	n/a	n/a	S4
Actinomyces israelii	645	https://www.homd.org/taxa/tax_description?otid=645	PRODUCES	menaquinone	metabolite	n/a	n/a	n/a	n/a	experimental	Tetrahydrogenated menaquinones with ten isoprene units [MK-10(H4)] are the major respiratory quinones.	Fernandez1987 10.1111/j.1574-6968.1987.tb02191.x	n/a	n/a	
Actinomyces israelii	645	https://www.homd.org/taxa/tax_description?otid=645	PRODUCES	nitrite	metabolite	HMDB0002786	https://hmdb.ca/metabolites/HMDB0002786	n/a	n/a	experimental	Data retrived from review and not original research article. Usually.	Bergey's Manual of Systematic Bacteriology, Vol. 3-5, Edn. 2nd.	n/a	n/a	S4
Actinomyces israelii	645	https://www.homd.org/taxa/tax_description?otid=645	USES	adenine	metabolite	HMDB0000034	https://hmdb.ca/metabolites/HMDB0000034	n/a	n/a	experimental	Data retrived from review and not original research article.	Bergey's Manual of Systematic Bacteriology, Vol. 3-5, Edn. 2nd.	n/a	n/a	S4
Actinomyces israelii	645	https://www.homd.org/taxa/tax_description?otid=645	USES	nitrate	metabolite	HMDB0002878	https://hmdb.ca/metabolites/HMDB0002878	n/a	n/a	experimental	Data retrived from review and not original research article. Usually.	Bergey's Manual of Systematic Bacteriology, Vol. 3-5, Edn. 2nd.	n/a	n/a	S4
Actinomyces israelii	645	https://www.homd.org/taxa/tax_description?otid=645	USES	thymine	metabolite	HMDB0000262	https://hmdb.ca/metabolites/HMDB0000262	n/a	n/a	experimental	Data retrived from review and not original research article.	Bergey's Manual of Systematic Bacteriology, Vol. 3-5, Edn. 2nd.	n/a	n/a	S4
Actinomyces oris	893	https://www.homd.org/taxa/tax_description?otid=893	PRODUCES	fucosidase	enzyme	n/a	n/a	n/a	n/a	experimental	Data retrived from review and not original research article. Some.	Bergey's Manual of Systematic Bacteriology, Vol. 3-5, Edn. 2nd.	n/a	n/a	S4
Actinomyces oris	893	https://www.homd.org/taxa/tax_description?otid=893	PRODUCES	lactic acid	metabolite	HMDB0000190	https://hmdb.ca/metabolites/HMDB0000190	n/a	n/a	experimental	Data retrived from review and not original research article.	Bergey's Manual of Systematic Bacteriology, Vol. 3-5, Edn. 2nd.	n/a	n/a	S4
Actinomyces oris	893	https://www.homd.org/taxa/tax_description?otid=893	PRODUCES	menaquinone	metabolite	n/a	n/a	n/a	n/a	prediction	Prediction based on the characteristics of Actinomyces genus.Tetrahydrogenated menaquinones with ten isoprene units [MK-10(H4)] are the major respiratory quinones.	Fernandez1987 10.1111/j.1574-6968.1987.tb02191.x	n/a	n/a	
Actinomyces oris	893	https://www.homd.org/taxa/tax_description?otid=893	PRODUCES	nitrite	metabolite	HMDB0002786	https://hmdb.ca/metabolites/HMDB0002786	n/a	n/a	experimental	Data retrived from review and not original research article. Variable.	Bergey's Manual of Systematic Bacteriology, Vol. 3-5, Edn. 2nd.	n/a	n/a	S4
Actinomyces oris	893	https://www.homd.org/taxa/tax_description?otid=893	USES	nitrate	metabolite	HMDB0002878	https://hmdb.ca/metabolites/HMDB0002878	n/a	n/a	experimental	Data retrived from review and not original research article. Variable.	Bergey's Manual of Systematic Bacteriology, Vol. 3-5, Edn. 2nd.	n/a	n/a	S4
Actinomyces sp. HMT-169	169	https://www.homd.org/taxa/tax_description?otid=169	PRODUCES	fucosidase	enzyme	n/a	n/a	n/a	n/a	prediction	Prediction based on the characteristics of related A. naselundii. Data retrived from review and not original research article. Some.	Bergey's Manual of Systematic Bacteriology, Vol. 3-5, Edn. 2nd.	n/a	n/a	S4
Actinomyces sp. HMT-169	169	https://www.homd.org/taxa/tax_description?otid=169	PRODUCES	lactic acid	metabolite	HMDB0000190	https://hmdb.ca/metabolites/HMDB0000190	n/a	n/a	prediction	Prediction based on the characteristics of related A. naselundii. Data retrived from review and not original research article.	Bergey's Manual of Systematic Bacteriology, Vol. 3-5, Edn. 2nd.	n/a	n/a	S4
Actinomyces sp. HMT-169	169	https://www.homd.org/taxa/tax_description?otid=169	PRODUCES	menaquinone	metabolite	n/a	n/a	n/a	n/a	prediction	Prediction based on the characteristics of Actinomyces genus.Tetrahydrogenated menaquinones with ten isoprene units [MK-10(H4)] are the major respiratory quinones.	Fernandez1987 10.1111/j.1574-6968.1987.tb02191.x	n/a	n/a	
Actinomyces sp. HMT-169	169	https://www.homd.org/taxa/tax_description?otid=169	PRODUCES	nitrite	metabolite	HMDB0002786	https://hmdb.ca/metabolites/HMDB0002786	n/a	n/a	prediction	Prediction based on the characteristics of related A. naselundii. Data retrived from review and not original research article.	Bergey's Manual of Systematic Bacteriology, Vol. 3-5, Edn. 2nd.	n/a	n/a	S4
Actinomyces sp. HMT-169	169	https://www.homd.org/taxa/tax_description?otid=169	USES	nitrate	metabolite	HMDB0002878	https://hmdb.ca/metabolites/HMDB0002878	n/a	n/a	prediction	Prediction based on the characteristics of related A. naselundii. Data retrived from review and not original research article.	Bergey's Manual of Systematic Bacteriology, Vol. 3-5, Edn. 2nd.	n/a	n/a	S4
Aggregatibacter aphrophilus	545	https://www.homd.org/taxa/tax_description?otid=545	PRODUCES	(proto)porphyrin	metabolite	n/a	n/a	n/a	n/a	experimental		NørskovLauritsen2006 10.1099/ijs.0.64207-0	https://pubmed.ncbi.nlm.nih.gov/16957111/	n/a	7c
Aggregatibacter aphrophilus	545	https://www.homd.org/taxa/tax_description?otid=545	PRODUCES	alkaline phosphatase	enzyme	n/a	n/a	3.1.3.1	https://www.genome.jp/entry/3.1.3.1	experimental		NørskovLauritsen2006 10.1099/ijs.0.64207-0	https://pubmed.ncbi.nlm.nih.gov/16957111/	n/a	7c
Aggregatibacter aphrophilus	545	https://www.homd.org/taxa/tax_description?otid=545	PRODUCES	beta-galactosidase	enzyme	n/a	n/a	3.2.1.23	https://www.genome.jp/entry/3.2.1.23	experimental		NørskovLauritsen2006 10.1099/ijs.0.64207-0	https://pubmed.ncbi.nlm.nih.gov/16957111/	n/a	7c
Aggregatibacter aphrophilus	545	https://www.homd.org/taxa/tax_description?otid=545	PRODUCES	cell aggregates	biofilm component	n/a	n/a	n/a	n/a	experimental		NørskovLauritsen2006 10.1099/ijs.0.64207-0	https://pubmed.ncbi.nlm.nih.gov/16957111/	n/a	7c
Aggregatibacter aphrophilus	545	https://www.homd.org/taxa/tax_description?otid=545	PRODUCES	nitrate reductase	enzyme	n/a	n/a	n/a	n/a	experimental		NørskovLauritsen2006 10.1099/ijs.0.64207-0	https://pubmed.ncbi.nlm.nih.gov/16957111/	n/a	7c
Aggregatibacter aphrophilus	545	https://www.homd.org/taxa/tax_description?otid=545	PRODUCES	nitrite	metabolite	HMDB0002786	https://hmdb.ca/metabolites/HMDB0002786	n/a	n/a	experimental		NørskovLauritsen2006 10.1099/ijs.0.64207-0	https://pubmed.ncbi.nlm.nih.gov/16957111/	n/a	7c
Aggregatibacter aphrophilus	545	https://www.homd.org/taxa/tax_description?otid=545	USES	alpha-lactose	metabolite	HMDB0000186	https://hmdb.ca/metabolites/HMDB0000186	n/a	n/a	experimental	Fermentation.	NørskovLauritsen2006 10.1099/ijs.0.64207-0	https://pubmed.ncbi.nlm.nih.gov/16957111/	n/a	7c
Aggregatibacter aphrophilus	545	https://www.homd.org/taxa/tax_description?otid=545	USES	carbon dioxide	metabolite	HMDB0001967	https://hmdb.ca/metabolites/HMDB0001967	n/a	n/a	experimental		NørskovLauritsen2006 10.1099/ijs.0.64207-0	https://pubmed.ncbi.nlm.nih.gov/16957111/	n/a	7c
Aggregatibacter aphrophilus	545	https://www.homd.org/taxa/tax_description?otid=545	USES	D-fructose	metabolite	HMDB0000660	https://hmdb.ca/metabolites/HMDB0000660	n/a	n/a	experimental	Fermentation.	NørskovLauritsen2006 10.1099/ijs.0.64207-0	https://pubmed.ncbi.nlm.nih.gov/16957111/	n/a	7c
Aggregatibacter aphrophilus	545	https://www.homd.org/taxa/tax_description?otid=545	USES	D-galactose	metabolite	HMDB0000143	https://hmdb.ca/metabolites/HMDB0000143	n/a	n/a	experimental	Fermentation.	NørskovLauritsen2006 10.1099/ijs.0.64207-0	https://pubmed.ncbi.nlm.nih.gov/16957111/	n/a	7c
Aggregatibacter aphrophilus	545	https://www.homd.org/taxa/tax_description?otid=545	USES	D-glucose	metabolite	HMDB0000122	https://hmdb.ca/metabolites/HMDB0000122	n/a	n/a	experimental	Fermentation.	NørskovLauritsen2006 10.1099/ijs.0.64207-0	https://pubmed.ncbi.nlm.nih.gov/16957111/	n/a	7c
Aggregatibacter aphrophilus	545	https://www.homd.org/taxa/tax_description?otid=545	USES	D-maltose	metabolite	HMDB0000163	https://hmdb.ca/metabolites/HMDB0000163	n/a	n/a	experimental	Fermentation.	NørskovLauritsen2006 10.1099/ijs.0.64207-0	https://pubmed.ncbi.nlm.nih.gov/16957111/	n/a	7c
Aggregatibacter aphrophilus	545	https://www.homd.org/taxa/tax_description?otid=545	USES	D-mannose	metabolite	HMDB0000169	https://hmdb.ca/metabolites/HMDB0000169	n/a	n/a	experimental	Fermentation.	NørskovLauritsen2006 10.1099/ijs.0.64207-0	https://pubmed.ncbi.nlm.nih.gov/16957111/	n/a	7c
Aggregatibacter aphrophilus	545	https://www.homd.org/taxa/tax_description?otid=545	USES	hemin	metabolite	HMDB0000887	https://hmdb.ca/metabolites/HMDB0000887	n/a	n/a	experimental		NørskovLauritsen2006 10.1099/ijs.0.64207-0	https://pubmed.ncbi.nlm.nih.gov/16957111/	n/a	7c
Aggregatibacter aphrophilus	545	https://www.homd.org/taxa/tax_description?otid=545	USES	lactic acid	metabolite	HMDB0000190	https://hmdb.ca/metabolites/HMDB0000190	n/a	n/a	prediction	Prediction based on the characteristics of related A. actinomycetemcomitnas species.	Brown2009 10.1371/journal.pone.0007864	https://pubmed.ncbi.nlm.nih.gov/19924225/	n/a	7c
Aggregatibacter aphrophilus	545	https://www.homd.org/taxa/tax_description?otid=545	USES	maltotriose	metabolite	HMDB0001262	https://hmdb.ca/metabolites/HMDB0001262	n/a	n/a	experimental	Fermentation.	NørskovLauritsen2006 10.1099/ijs.0.64207-0	https://pubmed.ncbi.nlm.nih.gov/16957111/	n/a	7c
Aggregatibacter aphrophilus	545	https://www.homd.org/taxa/tax_description?otid=545	USES	nicotinamide adenine dinucleotide	metabolite	HMDB0000902	https://hmdb.ca/metabolites/HMDB0000902	n/a	n/a	experimental		NørskovLauritsen2006 10.1099/ijs.0.64207-0	https://pubmed.ncbi.nlm.nih.gov/16957111/	n/a	7c
Aggregatibacter aphrophilus	545	https://www.homd.org/taxa/tax_description?otid=545	USES	nitrate	metabolite	HMDB0002878	https://hmdb.ca/metabolites/HMDB0002878	n/a	n/a	experimental		NørskovLauritsen2006 10.1099/ijs.0.64207-0	https://pubmed.ncbi.nlm.nih.gov/16957111/	n/a	7c
Aggregatibacter aphrophilus	545	https://www.homd.org/taxa/tax_description?otid=545	USES	raffinose	metabolite	HMDB0003213	https://hmdb.ca/metabolites/HMDB0003213	n/a	n/a	experimental	Fermentation.	NørskovLauritsen2006 10.1099/ijs.0.64207-0	https://pubmed.ncbi.nlm.nih.gov/16957111/	n/a	7c
Aggregatibacter aphrophilus	545	https://www.homd.org/taxa/tax_description?otid=545	USES	sucrose	metabolite	HMDB0000258	https://hmdb.ca/metabolites/HMDB0000258	n/a	n/a	experimental	Fermentation.	NørskovLauritsen2006 10.1099/ijs.0.64207-0	https://pubmed.ncbi.nlm.nih.gov/16957111/	n/a	7c
Aggregatibacter aphrophilus	545	https://www.homd.org/taxa/tax_description?otid=545	USES	trehalose	metabolite	HMDB0000775	https://hmdb.ca/metabolites/HMDB0000775	n/a	n/a	experimental	Fermentation.	NørskovLauritsen2006 10.1099/ijs.0.64207-0	https://pubmed.ncbi.nlm.nih.gov/16957111/	n/a	7c
Alloscardovia omnicolens	198	https://www.homd.org/taxa/tax_description?otid=198	IS_INHIBITED_BY	oxygen	metabolite	HMDB0001377	https://hmdb.ca/metabolites/HMDB0001377	n/a	n/a	experimental	Data retrived from review and not original research article.	Bergey's Manual of Systematic Bacteriology, Vol. 3-5, Edn. 2nd.	n/a	n/a	S4
Alloscardovia omnicolens	198	https://www.homd.org/taxa/tax_description?otid=198	PRODUCES	lactic acid	metabolite	HMDB0000190	https://hmdb.ca/metabolites/HMDB0000190	n/a	n/a	prediction	Prediction based on the characteristics of Bifidobacteriaceae family. Data retrived from review and not original research article.	Bergey's Manual of Systematic Bacteriology, Vol. 3-5, Edn. 2nd.	n/a	n/a	S4
Arachnia propionica	739	https://www.homd.org/taxa/tax_description?otid=739	DEGRADES	gelatine	metabolite	n/a	n/a	n/a	n/a	experimental	Data retrived from review and not original research article.	Bergey's Manual of Systematic Bacteriology, Vol. 3-5, Edn. 2nd.	n/a	n/a	7c
Arachnia propionica	739	https://www.homd.org/taxa/tax_description?otid=739	IS_A_HOST_FOR	Saccharibacteria bacterium HMT-488	taxon	n/a	n/a	n/a	n/a	experimental		Bor2020 10.1177/0022034520905792; Murugkar2020 10.1080/20002297.2020.1814666	https://pubmed.ncbi.nlm.nih.gov/32075512/	https://pubmed.ncbi.nlm.nih.gov/33209205/	7c
Arachnia propionica	739	https://www.homd.org/taxa/tax_description?otid=739	IS_A_HOST_FOR	Saccharibacteria bacterium HMT-955	taxon	n/a	n/a	n/a	n/a	experimental		Bor2020 10.1177/0022034520905792; Murugkar2020 10.1080/20002297.2020.1814666	https://pubmed.ncbi.nlm.nih.gov/32075512/	https://pubmed.ncbi.nlm.nih.gov/33209205/	7c
Arachnia propionica	739	https://www.homd.org/taxa/tax_description?otid=739	PRODUCES	alpha-galactosidase	enzyme	n/a	n/a	3.2.1.22	https://www.genome.jp/entry/3.2.1.22	experimental	Data retrived from review and not original research article.	Bergey's Manual of Systematic Bacteriology, Vol. 3-5, Edn. 2nd.	n/a	n/a	7c
Arachnia propionica	739	https://www.homd.org/taxa/tax_description?otid=739	PRODUCES	alpha-glucosidase	enzyme	n/a	n/a	3.2.1.20	https://www.genome.jp/entry/3.2.1.20	experimental	Data retrived from review and not original research article.	Bergey's Manual of Systematic Bacteriology, Vol. 3-5, Edn. 2nd.	n/a	n/a	7c
Arachnia propionica	739	https://www.homd.org/taxa/tax_description?otid=739	PRODUCES	enzyme degrading geletine	enzyme	n/a	n/a	n/a	n/a	experimental	Data retrived from review and not original research article.	Bergey's Manual of Systematic Bacteriology, Vol. 3-5, Edn. 2nd.	n/a	n/a	7c
Arachnia propionica	739	https://www.homd.org/taxa/tax_description?otid=739	PRODUCES	esterase (C4)	enzyme	n/a	n/a	n/a	n/a	experimental	Data retrived from review and not original research article.	Bergey's Manual of Systematic Bacteriology, Vol. 3-5, Edn. 2nd.	n/a	n/a	7c
Arachnia propionica	739	https://www.homd.org/taxa/tax_description?otid=739	PRODUCES	esterase (C8)	enzyme	n/a	n/a	n/a	n/a	experimental	Data retrived from review and not original research article.	Bergey's Manual of Systematic Bacteriology, Vol. 3-5, Edn. 2nd.	n/a	n/a	7c
Arachnia propionica	739	https://www.homd.org/taxa/tax_description?otid=739	PRODUCES	hyaluronidase	enzyme	n/a	n/a	3.2.1.35	https://www.genome.jp/entry/3.2.1.35	experimental	Data retrived from review and not original research article.	Bergey's Manual of Systematic Bacteriology, Vol. 3-5, Edn. 2nd.	n/a	n/a	7c
Arachnia propionica	739	https://www.homd.org/taxa/tax_description?otid=739	PRODUCES	menaquinone	metabolite	n/a	n/a	n/a	n/a	experimental	Tetrahydrogenated menaquinones with nine isoprene units [MK-9(H4)] are the major respiratory quinones.	Fernandez1987 10.1111/j.1574-6968.1987.tb02191.x	n/a	n/a	7c
Arachnia propionica	739	https://www.homd.org/taxa/tax_description?otid=739	PRODUCES	N-acetyl-beta-glucosaminidase	enzyme	n/a	n/a	3.2.1.52	https://www.genome.jp/entry/3.2.1.52	experimental	Data retrived from review and not original research article.	Bergey's Manual of Systematic Bacteriology, Vol. 3-5, Edn. 2nd.	n/a	n/a	7c
Arachnia propionica	739	https://www.homd.org/taxa/tax_description?otid=739	PRODUCES	nitrate reductase	enzyme	n/a	n/a	n/a	n/a	experimental	Data retrived from review and not original research article.	Bergey's Manual of Systematic Bacteriology, Vol. 3-5, Edn. 2nd.	n/a	n/a	7c
Arachnia propionica	739	https://www.homd.org/taxa/tax_description?otid=739	PRODUCES	nitrite	metabolite	HMDB0002786	https://hmdb.ca/metabolites/HMDB0002786	n/a	n/a	experimental	Data retrived from review and not original research article.	Bergey's Manual of Systematic Bacteriology, Vol. 3-5, Edn. 2nd.	n/a	n/a	7c
Arachnia propionica	739	https://www.homd.org/taxa/tax_description?otid=739	PRODUCES	propionic acid	metabolite	HMDB0000237	https://hmdb.ca/metabolites/HMDB0000237	n/a	n/a	experimental	Data retrived from review and not original research article.	Bergey's Manual of Systematic Bacteriology, Vol. 3-5, Edn. 2nd.	n/a	n/a	7c
Arachnia propionica	739	https://www.homd.org/taxa/tax_description?otid=739	USES	alpha-lactose	metabolite	HMDB0000186	https://hmdb.ca/metabolites/HMDB0000186	n/a	n/a	experimental	Data retrived from review and not original research article.	Bergey's Manual of Systematic Bacteriology, Vol. 3-5, Edn. 2nd.	n/a	n/a	7c
Arachnia propionica	739	https://www.homd.org/taxa/tax_description?otid=739	USES	carbon dioxide	metabolite	HMDB0001967	https://hmdb.ca/metabolites/HMDB0001967	n/a	n/a	experimental	Data retrived from review and not original research article.	Bergey's Manual of Systematic Bacteriology, Vol. 3-5, Edn. 2nd.	n/a	n/a	7c
Arachnia propionica	739	https://www.homd.org/taxa/tax_description?otid=739	USES	D-fructose	metabolite	HMDB0000660	https://hmdb.ca/metabolites/HMDB0000660	n/a	n/a	experimental	Data retrived from review and not original research article.	Bergey's Manual of Systematic Bacteriology, Vol. 3-5, Edn. 2nd.	n/a	n/a	7c
Arachnia propionica	739	https://www.homd.org/taxa/tax_description?otid=739	USES	D-galactose	metabolite	HMDB0000143	https://hmdb.ca/metabolites/HMDB0000143	n/a	n/a	experimental	Data retrived from review and not original research article.	Bergey's Manual of Systematic Bacteriology, Vol. 3-5, Edn. 2nd.	n/a	n/a	7c
Arachnia propionica	739	https://www.homd.org/taxa/tax_description?otid=739	USES	D-glucose	metabolite	HMDB0000122	https://hmdb.ca/metabolites/HMDB0000122	n/a	n/a	experimental	Data retrived from review and not original research article.	Bergey's Manual of Systematic Bacteriology, Vol. 3-5, Edn. 2nd.	n/a	n/a	7c
Arachnia propionica	739	https://www.homd.org/taxa/tax_description?otid=739	USES	D-maltose	metabolite	HMDB0000163	https://hmdb.ca/metabolites/HMDB0000163	n/a	n/a	experimental	Data retrived from review and not original research article.	Bergey's Manual of Systematic Bacteriology, Vol. 3-5, Edn. 2nd.	n/a	n/a	7c
Arachnia propionica	739	https://www.homd.org/taxa/tax_description?otid=739	USES	D-mannose	metabolite	HMDB0000169	https://hmdb.ca/metabolites/HMDB0000169	n/a	n/a	experimental	Data retrived from review and not original research article.	Bergey's Manual of Systematic Bacteriology, Vol. 3-5, Edn. 2nd.	n/a	n/a	7c
Arachnia propionica	739	https://www.homd.org/taxa/tax_description?otid=739	USES	lactic acid	metabolite	HMDB0000190	https://hmdb.ca/metabolites/HMDB0000190	n/a	n/a	experimental	Data retrived from review and not original research article.	Bergey's Manual of Systematic Bacteriology, Vol. 3-5, Edn. 2nd.	n/a	n/a	7c
Arachnia propionica	739	https://www.homd.org/taxa/tax_description?otid=739	USES	nitrate	metabolite	HMDB0002878	https://hmdb.ca/metabolites/HMDB0002878	n/a	n/a	experimental	Data retrived from review and not original research article.	Bergey's Manual of Systematic Bacteriology, Vol. 3-5, Edn. 2nd.	n/a	n/a	7c
Arachnia propionica	739	https://www.homd.org/taxa/tax_description?otid=739	USES	raffinose	metabolite	HMDB0003213	https://hmdb.ca/metabolites/HMDB0003213	n/a	n/a	experimental	Data retrived from review and not original research article.	Bergey's Manual of Systematic Bacteriology, Vol. 3-5, Edn. 2nd.	n/a	n/a	7c
Arachnia propionica	739	https://www.homd.org/taxa/tax_description?otid=739	USES	salicin	metabolite	HMDB0003546	https://hmdb.ca/metabolites/HMDB0003546	n/a	n/a	experimental	Data retrived from review and not original research article.	Bergey's Manual of Systematic Bacteriology, Vol. 3-5, Edn. 2nd.	n/a	n/a	7c
Arachnia propionica	739	https://www.homd.org/taxa/tax_description?otid=739	USES	starch	metabolite	n/a	n/a	n/a	n/a	experimental	Data retrived from review and not original research article.	Bergey's Manual of Systematic Bacteriology, Vol. 3-5, Edn. 2nd.	n/a	n/a	7c
Arachnia propionica	739	https://www.homd.org/taxa/tax_description?otid=739	USES	sucrose	metabolite	HMDB0000258	https://hmdb.ca/metabolites/HMDB0000258	n/a	n/a	experimental	Data retrived from review and not original research article.	Bergey's Manual of Systematic Bacteriology, Vol. 3-5, Edn. 2nd.	n/a	n/a	7c
Arachnia propionica	739	https://www.homd.org/taxa/tax_description?otid=739	USES	trehalose	metabolite	HMDB0000775	https://hmdb.ca/metabolites/HMDB0000775	n/a	n/a	experimental	Data retrived from review and not original research article.	Bergey's Manual of Systematic Bacteriology, Vol. 3-5, Edn. 2nd.	n/a	n/a	7c
Bifidobacterium dentium	588	https://www.homd.org/taxa/tax_description?otid=588	IS_INHIBITED_BY	oxygen	metabolite	HMDB0001377	https://hmdb.ca/metabolites/HMDB0001377	n/a	n/a	experimental	Data retrived from review and not original research article.	Bergey's Manual of Systematic Bacteriology, Vol. 3-5, Edn. 2nd.	n/a	n/a	S4
Bifidobacterium dentium	588	https://www.homd.org/taxa/tax_description?otid=588	PRODUCES	amino acids	metabolite	n/a	n/a	n/a	n/a	experimental	Data retrived from review and not original research article.	Bergey's Manual of Systematic Bacteriology, Vol. 3-5, Edn. 2nd.	n/a	n/a	S4
Bifidobacterium dentium	588	https://www.homd.org/taxa/tax_description?otid=588	PRODUCES	lactic acid	metabolite	HMDB0000190	https://hmdb.ca/metabolites/HMDB0000190	n/a	n/a	experimental	Data retrived from review and not original research article.	Bergey's Manual of Systematic Bacteriology, Vol. 3-5, Edn. 2nd.	n/a	n/a	S4
Bifidobacterium dentium	588	https://www.homd.org/taxa/tax_description?otid=588	USES	menaquinone	metabolite	n/a	n/a	n/a	n/a	experimental	The growth of most Bifidobacterium isolates, except that of B. urinalis, was stimulated by vitamin K.	Hojo2007 10.1111/j.1365-2672.2007.03436.x	https://pubmed.ncbi.nlm.nih.gov/17953607/	n/a	S4
Bifidobacterium dentium	588	https://www.homd.org/taxa/tax_description?otid=588	USES	pantothenic acid	metabolite	HMDB0000210	https://hmdb.ca/metabolites/HMDB0000210	n/a	n/a	experimental	Data retrived from review and not original research article.	Bergey's Manual of Systematic Bacteriology, Vol. 3-5, Edn. 2nd.	n/a	n/a	S4
Bifidobacterium dentium	588	https://www.homd.org/taxa/tax_description?otid=588	USES	riboflavin	metabolite	HMDB0000244	https://hmdb.ca/metabolites/HMDB0000244	n/a	n/a	experimental	Data retrived from review and not original research article.	Bergey's Manual of Systematic Bacteriology, Vol. 3-5, Edn. 2nd.	n/a	n/a	S4
Bifidobacterium longum	862	https://www.homd.org/taxa/tax_description?otid=862	IS_INHIBITED_BY	oxygen	metabolite	HMDB0001377	https://hmdb.ca/metabolites/HMDB0001377	n/a	n/a	experimental	Data retrived from review and not original research article.	Bergey's Manual of Systematic Bacteriology, Vol. 3-5, Edn. 2nd.	n/a	n/a	S4
Bifidobacterium longum	862	https://www.homd.org/taxa/tax_description?otid=862	PRODUCES	amino acids	metabolite	n/a	n/a	n/a	n/a	experimental	Data retrived from review and not original research article.	Bergey's Manual of Systematic Bacteriology, Vol. 3-5, Edn. 2nd.	n/a	n/a	S4
Bifidobacterium longum	862	https://www.homd.org/taxa/tax_description?otid=862	PRODUCES	lactic acid	metabolite	HMDB0000190	https://hmdb.ca/metabolites/HMDB0000190	n/a	n/a	experimental	Data retrived from review and not original research article.	Bergey's Manual of Systematic Bacteriology, Vol. 3-5, Edn. 2nd.	n/a	n/a	S4
Bifidobacterium longum	862	https://www.homd.org/taxa/tax_description?otid=862	USES	menaquinone	metabolite	n/a	n/a	n/a	n/a	experimental	The growth of most Bifidobacterium isolates, except that of B. urinalis, was stimulated by vitamin K.	Hojo2007 10.1111/j.1365-2672.2007.03436.x	https://pubmed.ncbi.nlm.nih.gov/17953607/	n/a	S4
Capnocytophaga leadbetteri	329	https://www.homd.org/taxa/tax_description?otid=329	USES	carbon dioxide	metabolite	HMDB0001967	https://hmdb.ca/metabolites/HMDB0001967	n/a	n/a	prediction	Prediction based on the characteristics of Capnocytophaga genus. Data retrived from review and not original research article.	Bergey's Manual of Systematic Bacteriology, Vol. 3-5, Edn. 2nd.	n/a	n/a	S4
Capnocytophaga sputigena	775	https://www.homd.org/taxa/tax_description?otid=775	IS_INHIBITED_BY	oxygen	metabolite	HMDB0001377	https://hmdb.ca/metabolites/HMDB0001377	n/a	n/a	experimental	Data retrived from review and not original research article.	Bergey's Manual of Systematic Bacteriology, Vol. 3-5, Edn. 2nd.	n/a	n/a	S4
Capnocytophaga sputigena	775	https://www.homd.org/taxa/tax_description?otid=775	PRODUCES	nitrite	metabolite	HMDB0002786	https://hmdb.ca/metabolites/HMDB0002786	n/a	n/a	experimental	Data retrived from review and not original research article.	Bergey's Manual of Systematic Bacteriology, Vol. 3-5, Edn. 2nd.	n/a	n/a	S4
Capnocytophaga sputigena	775	https://www.homd.org/taxa/tax_description?otid=775	PRODUCES	peptides	metabolite	n/a	n/a	n/a	n/a	experimental	Data retrived from review and not original research article.	Bergey's Manual of Systematic Bacteriology, Vol. 3-5, Edn. 2nd.	n/a	n/a	S4
Capnocytophaga sputigena	775	https://www.homd.org/taxa/tax_description?otid=775	USES	carbon dioxide	metabolite	HMDB0001967	https://hmdb.ca/metabolites/HMDB0001967	n/a	n/a	experimental	Data retrived from review and not original research article.	Bergey's Manual of Systematic Bacteriology, Vol. 3-5, Edn. 2nd.	n/a	n/a	S4
Capnocytophaga sputigena	775	https://www.homd.org/taxa/tax_description?otid=775	USES	nitrate	metabolite	HMDB0002878	https://hmdb.ca/metabolites/HMDB0002878	n/a	n/a	experimental	Data retrived from review and not original research article.	Bergey's Manual of Systematic Bacteriology, Vol. 3-5, Edn. 2nd.	n/a	n/a	S4
Corynebacterium matruchotii	666	https://www.homd.org/taxa/tax_description?otid=666	DEGRADES	gelatine	metabolite	n/a	n/a	n/a	n/a	experimental	Data retrived from review and not original research article.	Bergey's Manual of Systematic Bacteriology, Vol. 3-5, Edn. 2nd.	n/a	n/a	7c
Corynebacterium matruchotii	666	https://www.homd.org/taxa/tax_description?otid=666	DEGRADES	hydrogen peroxide	metabolite	HMDB0003125	https://hmdb.ca/metabolites/HMDB0003125	n/a	n/a	experimental	C. matruchotii enhanced the fitness of streptococci dependent on its ability to detoxify streptococcal-produced hydrogen peroxide and its ability to oxidize lactate also produced by streptococci.	Almeida2023 10.1128/msystems.00115-23	https://pubmed.ncbi.nlm.nih.gov/37610230/	n/a	7c
Corynebacterium matruchotii	666	https://www.homd.org/taxa/tax_description?otid=666	PRODUCES	alkaline phosphatase	enzyme	n/a	n/a	3.1.3.1	https://www.genome.jp/entry/3.1.3.1	experimental	Data retrived from review and not original research article.	Bergey's Manual of Systematic Bacteriology, Vol. 3-5, Edn. 2nd.	n/a	n/a	7c
Corynebacterium matruchotii	666	https://www.homd.org/taxa/tax_description?otid=666	PRODUCES	biofilm scaffold	biofilm component	n/a	n/a	n/a	n/a	experimental	Data retrived from review and not original research article.	Bergey's Manual of Systematic Bacteriology, Vol. 3-5, Edn. 2nd.	n/a	n/a	7c
Corynebacterium matruchotii	666	https://www.homd.org/taxa/tax_description?otid=666	PRODUCES	catalase	enzyme	n/a	n/a	1.11.1.6	https://www.genome.jp/entry/1.11.1.6	experimental	Catalase was maximally expressed aerobically and not expressed anaerobically. C. matruchotii ΔkatA had to be generated entirely under anaerobic conditions and does not survive incubation in aerobic or microaerophilic conditions.	Almeida2023 10.1128/msystems.00115-23	https://pubmed.ncbi.nlm.nih.gov/37610230/	n/a	7c
Corynebacterium matruchotii	666	https://www.homd.org/taxa/tax_description?otid=666	PRODUCES	enzyme degrading geletine	enzyme	n/a	n/a	n/a	n/a	experimental	Data retrived from review and not original research article.	Bergey's Manual of Systematic Bacteriology, Vol. 3-5, Edn. 2nd.	n/a	n/a	7c
Corynebacterium matruchotii	666	https://www.homd.org/taxa/tax_description?otid=666	PRODUCES	menaquinone	metabolite	n/a	n/a	n/a	n/a	experimental	Data retrived from review and not original research article.	Bergey's Manual of Systematic Bacteriology, Vol. 3-5, Edn. 2nd.	n/a	n/a	7c
Corynebacterium matruchotii	666	https://www.homd.org/taxa/tax_description?otid=666	PRODUCES	nitrate reductase	enzyme	n/a	n/a	n/a	n/a	experimental	Data retrived from review and not original research article.	Bergey's Manual of Systematic Bacteriology, Vol. 3-5, Edn. 2nd.	n/a	n/a	7c
Corynebacterium matruchotii	666	https://www.homd.org/taxa/tax_description?otid=666	PRODUCES	nitrite	metabolite	HMDB0002786	https://hmdb.ca/metabolites/HMDB0002786	n/a	n/a	experimental	Data retrived from review and not original research article.	Bergey's Manual of Systematic Bacteriology, Vol. 3-5, Edn. 2nd.	n/a	n/a	7c
Corynebacterium matruchotii	666	https://www.homd.org/taxa/tax_description?otid=666	PRODUCES	propionic acid	metabolite	HMDB0000237	https://hmdb.ca/metabolites/HMDB0000237	n/a	n/a	experimental	Data retrived from review and not original research article.	Bergey's Manual of Systematic Bacteriology, Vol. 3-5, Edn. 2nd.	n/a	n/a	7c
Corynebacterium matruchotii	666	https://www.homd.org/taxa/tax_description?otid=666	USES	dextrin	metabolite	HMDB0006857	https://hmdb.ca/metabolites/HMDB0006857	n/a	n/a	experimental	Fermentation. Data retrived from review and not original research article.	Bergey's Manual of Systematic Bacteriology, Vol. 3-5, Edn. 2nd.	n/a	n/a	7c
Corynebacterium matruchotii	666	https://www.homd.org/taxa/tax_description?otid=666	USES	D-fructose	metabolite	HMDB0000660	https://hmdb.ca/metabolites/HMDB0000660	n/a	n/a	experimental	Fermentation. Data retrived from review and not original research article.	Bergey's Manual of Systematic Bacteriology, Vol. 3-5, Edn. 2nd.	n/a	n/a	7c
Corynebacterium matruchotii	666	https://www.homd.org/taxa/tax_description?otid=666	USES	D-glucose	metabolite	HMDB0000122	https://hmdb.ca/metabolites/HMDB0000122	n/a	n/a	experimental	Fermentation. Data retrived from review and not original research article.	Bergey's Manual of Systematic Bacteriology, Vol. 3-5, Edn. 2nd.	n/a	n/a	7c
Corynebacterium matruchotii	666	https://www.homd.org/taxa/tax_description?otid=666	USES	D-maltose	metabolite	HMDB0000163	https://hmdb.ca/metabolites/HMDB0000163	n/a	n/a	experimental	Fermentation. Data retrived from review and not original research article.	Bergey's Manual of Systematic Bacteriology, Vol. 3-5, Edn. 2nd.	n/a	n/a	7c
Corynebacterium matruchotii	666	https://www.homd.org/taxa/tax_description?otid=666	USES	D-mannose	metabolite	HMDB0000169	https://hmdb.ca/metabolites/HMDB0000169	n/a	n/a	experimental	Fermentation. Data retrived from review and not original research article.	Bergey's Manual of Systematic Bacteriology, Vol. 3-5, Edn. 2nd.	n/a	n/a	7c
Corynebacterium matruchotii	666	https://www.homd.org/taxa/tax_description?otid=666	USES	lactic acid	metabolite	HMDB0000190	https://hmdb.ca/metabolites/HMDB0000190	n/a	n/a	experimental	C. matruchotii enhanced the fitness of streptococci dependent on its ability to detoxify streptococcal-produced hydrogen peroxide and its ability to oxidize lactate also produced by streptococci.	Almeida2023 10.1128/msystems.00115-23	https://pubmed.ncbi.nlm.nih.gov/37610230/	n/a	7c
Corynebacterium matruchotii	666	https://www.homd.org/taxa/tax_description?otid=666	USES	nitrate	metabolite	HMDB0002878	https://hmdb.ca/metabolites/HMDB0002878	n/a	n/a	experimental	Data retrived from review and not original research article.	Bergey's Manual of Systematic Bacteriology, Vol. 3-5, Edn. 2nd.	n/a	n/a	7c
Corynebacterium matruchotii	666	https://www.homd.org/taxa/tax_description?otid=666	USES	raffinose	metabolite	HMDB0003213	https://hmdb.ca/metabolites/HMDB0003213	n/a	n/a	experimental	Fermentation. Data retrived from review and not original research article.	Bergey's Manual of Systematic Bacteriology, Vol. 3-5, Edn. 2nd.	n/a	n/a	7c
Corynebacterium matruchotii	666	https://www.homd.org/taxa/tax_description?otid=666	USES	salicin	metabolite	HMDB0003546	https://hmdb.ca/metabolites/HMDB0003546	n/a	n/a	experimental	Fermentation. Data retrived from review and not original research article.	Bergey's Manual of Systematic Bacteriology, Vol. 3-5, Edn. 2nd.	n/a	n/a	7c
Corynebacterium matruchotii	666	https://www.homd.org/taxa/tax_description?otid=666	USES	starch	metabolite	n/a	n/a	n/a	n/a	experimental	Fermentation. Data retrived from review and not original research article.	Bergey's Manual of Systematic Bacteriology, Vol. 3-5, Edn. 2nd.	n/a	n/a	7c
Corynebacterium matruchotii	666	https://www.homd.org/taxa/tax_description?otid=666	USES	sucrose	metabolite	HMDB0000258	https://hmdb.ca/metabolites/HMDB0000258	n/a	n/a	experimental	Fermentation. Data retrived from review and not original research article.	Bergey's Manual of Systematic Bacteriology, Vol. 3-5, Edn. 2nd.	n/a	n/a	7c
Eikenalla corodens	577	https://www.homd.org/taxa/tax_description?otid=577	PRODUCES	nitric oxide	metabolite	HMDB0003378	https://hmdb.ca/metabolites/HMDB0003378	n/a	n/a	experimental	Data retrived from review and not original research article.	Bergey's Manual of Systematic Bacteriology, Vol. 3-5, Edn. 2nd.	n/a	n/a	S4
Eikenalla corodens	577	https://www.homd.org/taxa/tax_description?otid=577	PRODUCES	nitrite	metabolite	HMDB0002786	https://hmdb.ca/metabolites/HMDB0002786	n/a	n/a	experimental	Data retrived from review and not original research article.	Bergey's Manual of Systematic Bacteriology, Vol. 3-5, Edn. 2nd.	n/a	n/a	S4
Eikenalla corodens	577	https://www.homd.org/taxa/tax_description?otid=577	PRODUCES	proline aminopeptidase	enzyme	n/a	n/a	n/a	n/a	experimental	Data retrived from review and not original research article.	Bergey's Manual of Systematic Bacteriology, Vol. 3-5, Edn. 2nd.	n/a	n/a	S4
Eikenalla corodens	577	https://www.homd.org/taxa/tax_description?otid=577	USES	carbon dioxide	metabolite	HMDB0001967	https://hmdb.ca/metabolites/HMDB0001967	n/a	n/a	experimental	Data retrived from review and not original research article.	Bergey's Manual of Systematic Bacteriology, Vol. 3-5, Edn. 2nd.	n/a	n/a	S4
Eikenalla corodens	577	https://www.homd.org/taxa/tax_description?otid=577	USES	hemin	metabolite	HMDB0000887	https://hmdb.ca/metabolites/HMDB0000887	n/a	n/a	experimental	Data retrived from review and not original research article.	Bergey's Manual of Systematic Bacteriology, Vol. 3-5, Edn. 2nd.	n/a	n/a	S4
Eikenalla corodens	577	https://www.homd.org/taxa/tax_description?otid=577	USES	nitrate	metabolite	HMDB0002878	https://hmdb.ca/metabolites/HMDB0002878	n/a	n/a	experimental	Data retrived from review and not original research article.	Bergey's Manual of Systematic Bacteriology, Vol. 3-5, Edn. 2nd.	n/a	n/a	S4
Eikenalla corodens	577	https://www.homd.org/taxa/tax_description?otid=577	USES	nitrite	metabolite	HMDB0002786	https://hmdb.ca/metabolites/HMDB0002786	n/a	n/a	experimental	Data retrived from review and not original research article.	Bergey's Manual of Systematic Bacteriology, Vol. 3-5, Edn. 2nd.	n/a	n/a	S4
Eikenalla corodens	577	https://www.homd.org/taxa/tax_description?otid=577	USES	oxygen	metabolite	HMDB0001377	https://hmdb.ca/metabolites/HMDB0001377	n/a	n/a	experimental	Data retrived from review and not original research article.	Bergey's Manual of Systematic Bacteriology, Vol. 3-5, Edn. 2nd.	n/a	n/a	S4
Fusobacterium sp. OTU_27	n/a	n/a	IS_INHIBITED_BY	oxygen	metabolite	HMDB0001377	https://hmdb.ca/metabolites/HMDB0001377	n/a	n/a	prediction	Prediction based on the characteristics of Fusobacterium genus. Data retrived from review and not original research article.	Bergey's Manual of Systematic Bacteriology, Vol. 3-5, Edn. 2nd.	n/a	n/a	S4
Fusobacterium sp. OTU_27	n/a	n/a	PRODUCES	butyric acid	metabolite	HMDB0000039	https://hmdb.ca/metabolites/HMDB0000039	n/a	n/a	prediction	Prediction based on the characteristics of Fusobacterium genus. Data retrived from review and not original research article.	Bergey's Manual of Systematic Bacteriology, Vol. 3-5, Edn. 2nd.	n/a	n/a	S4
Fusobacterium sp. OTU_27	n/a	n/a	PRODUCES	hydrogen sulfide	metabolite	HMDB0003276	https://hmdb.ca/metabolites/HMDB0003276	n/a	n/a	prediction	Prediction based on the characteristics of Fusobacterium genus. Data retrived from review and not original research article.	Bergey's Manual of Systematic Bacteriology, Vol. 3-5, Edn. 2nd.	n/a	n/a	S4
Fusobacterium sp. OTU_27	n/a	n/a	PRODUCES	indole	metabolite	HMDB0000738	https://hmdb.ca/metabolites/HMDB0000738	n/a	n/a	prediction	Prediction based on the characteristics of Fusobacterium genus. Data retrived from review and not original research article.	Bergey's Manual of Systematic Bacteriology, Vol. 3-5, Edn. 2nd.	n/a	n/a	S4
Fusobacterium sp. OTU_27	n/a	n/a	USES	L-cysteine	metabolite	HMDB0000574	https://hmdb.ca/metabolites/HMDB0000574	n/a	n/a	prediction	Prediction based on the characteristics of Fusobacterium genus. Data retrived from review and not original research article.	Bergey's Manual of Systematic Bacteriology, Vol. 3-5, Edn. 2nd.	n/a	n/a	S4

Fusobacterium sp. OTU_27	n/a	n/a	USES	L-lysine	metabolite	HMDB0000182	https://hmdb.ca/metabolites/HMDB0000182	n/a	n/a	prediction	Prediction based on the characteristics of Fusobacterium genus.	Sakanaka2022 10.1128/msystems.00170-22	https://pubmed.ncbi.nlm.nih.gov/35852319/	n/a	S4
Fusobacterium sp. OTU_27	n/a	n/a	USES	L-ornithine	metabolite	HMDB0000214	https://hmdb.ca/metabolites/HMDB0000214	n/a	n/a	prediction	Prediction based on the characteristics of Fusobacterium genus.	Sakanaka2022 10.1128/msystems.00170-22	https://pubmed.ncbi.nlm.nih.gov/35852319/	n/a	S4
Granulicatella adiacens	534	https://www.homd.org/taxa/tax_description?otid=534	PRODUCES	lactic acid	metabolite	HMDB0000190	https://hmdb.ca/metabolites/HMDB0000190	n/a	n/a	experimental	Data retrived from review and not original research article.	Bergey's Manual of Systematic Bacteriology, Vol. 3-5, Edn. 2nd.	n/a	n/a	S4
Granulicatella adiacens	534	https://www.homd.org/taxa/tax_description?otid=534	USES	L-cysteine	metabolite	HMDB0000574	https://hmdb.ca/metabolites/HMDB0000574	n/a	n/a	experimental	Data retrived from review and not original research article.	Bergey's Manual of Systematic Bacteriology, Vol. 3-5, Edn. 2nd.	n/a	n/a	S4
Granulicatella adiacens	534	https://www.homd.org/taxa/tax_description?otid=534	USES	pyridoxal	metabolite	HMDB0001545	https://hmdb.ca/metabolites/HMDB0001545	n/a	n/a	experimental	Data retrived from review and not original research article.	Bergey's Manual of Systematic Bacteriology, Vol. 3-5, Edn. 2nd.	n/a	n/a	S4
Haemophilus parainfluenzae	718	https://www.homd.org/taxa/tax_description?otid=718	PRODUCES	catalase	enzyme	n/a	n/a	1.11.1.6	https://www.genome.jp/entry/1.11.1.6	experimental	Data retrived from review and not original research article. Variable.	Bergey's Manual of Systematic Bacteriology, Vol. 3-5, Edn. 2nd.	n/a	n/a	S4
Haemophilus parainfluenzae	718	https://www.homd.org/taxa/tax_description?otid=718	PRODUCES	nitrite	metabolite	HMDB0002786	https://hmdb.ca/metabolites/HMDB0002786	n/a	n/a	experimental	Data retrived from review and not original research article.	Bergey's Manual of Systematic Bacteriology, Vol. 3-5, Edn. 2nd.	n/a	n/a	S4
Haemophilus parainfluenzae	718	https://www.homd.org/taxa/tax_description?otid=718	PRODUCES	urease	enzyme	n/a	n/a	3.5.1.5	https://www.genome.jp/entry/3.5.1.5	experimental	Data retrived from review and not original research article. Variable.	Bergey's Manual of Systematic Bacteriology, Vol. 3-5, Edn. 2nd.	n/a	n/a	S4
Haemophilus parainfluenzae	718	https://www.homd.org/taxa/tax_description?otid=718	USES	fumaric acid	metabolite	HMDB0000134	https://hmdb.ca/metabolites/HMDB0000134	n/a	n/a	experimental	Data retrived from review and not original research article.	Bergey's Manual of Systematic Bacteriology, Vol. 3-5, Edn. 2nd.	n/a	n/a	S4
Haemophilus parainfluenzae	718	https://www.homd.org/taxa/tax_description?otid=718	USES	nitrate	metabolite	HMDB0002878	https://hmdb.ca/metabolites/HMDB0002878	n/a	n/a	experimental	Data retrived from review and not original research article.	Bergey's Manual of Systematic Bacteriology, Vol. 3-5, Edn. 2nd.	n/a	n/a	S4
Haemophilus parainfluenzae	718	https://www.homd.org/taxa/tax_description?otid=718	USES	oxygen	metabolite	HMDB0001377	https://hmdb.ca/metabolites/HMDB0001377	n/a	n/a	experimental	Data retrived from review and not original research article.	Bergey's Manual of Systematic Bacteriology, Vol. 3-5, Edn. 2nd.	n/a	n/a	S4
Haemophilus parainfluenzae	718	https://www.homd.org/taxa/tax_description?otid=718	USES	V-factor	metabolite	n/a	n/a	n/a	n/a	experimental	Data retrived from review and not original research article.	Bergey's Manual of Systematic Bacteriology, Vol. 3-5, Edn. 2nd.	n/a	n/a	S4
Kingella oralis	706	https://www.homd.org/taxa/tax_description?otid=706	PRODUCES	hydrogen sulfide	metabolite	HMDB0003276	https://hmdb.ca/metabolites/HMDB0003276	n/a	n/a	experimental	Data retrived from review and not original research article.	Bergey's Manual of Systematic Bacteriology, Vol. 3-5, Edn. 2nd.	n/a	n/a	S4
Kingella oralis	706	https://www.homd.org/taxa/tax_description?otid=706	USES	carbon dioxide	metabolite	HMDB0001967	https://hmdb.ca/metabolites/HMDB0001967	n/a	n/a	prediction	Prediction based on the characteristics of Kingella genus. Data retrived from review and not original research article.	Bergey's Manual of Systematic Bacteriology, Vol. 3-5, Edn. 2nd.	n/a	n/a	S4
Kingella oralis	706	https://www.homd.org/taxa/tax_description?otid=706	USES	nitrite	metabolite	HMDB0002786	https://hmdb.ca/metabolites/HMDB0002786	n/a	n/a	experimental	Data retrived from review and not original research article.	Bergey's Manual of Systematic Bacteriology, Vol. 3-5, Edn. 2nd.	n/a	n/a	S4
Kingella oralis	706	https://www.homd.org/taxa/tax_description?otid=706	USES	oxygen	metabolite	HMDB0001377	https://hmdb.ca/metabolites/HMDB0001377	n/a	n/a	experimental	Data retrived from review and not original research article.	Bergey's Manual of Systematic Bacteriology, Vol. 3-5, Edn. 2nd.	n/a	n/a	S4
Lactobacillus sp. OTU_19	n/a	n/a	IS_INHIBITED_BY	oxygen	metabolite	HMDB0001377	https://hmdb.ca/metabolites/HMDB0001377	n/a	n/a	prediction	Prediction based on the characteristics of related L. paracasei. Data retrived from review and not original research article.	Bergey's Manual of Systematic Bacteriology, Vol. 3-5, Edn. 2nd.	n/a	n/a	S4
Lactobacillus sp. OTU_19	n/a	n/a	PRODUCES	lactic acid	metabolite	HMDB0000190	https://hmdb.ca/metabolites/HMDB0000190	n/a	n/a	prediction	Prediction based on the characteristics of related L. paracasei. Data retrived from review and not original research article.	Bergey's Manual of Systematic Bacteriology, Vol. 3-5, Edn. 2nd.	n/a	n/a	S4
Lactobacillus sp. OTU_19	n/a	n/a	USES	carbon dioxide	metabolite	HMDB0001967	https://hmdb.ca/metabolites/HMDB0001967	n/a	n/a	prediction	Prediction based on the characteristics of related L. paracasei. Data retrived from review and not original research article.	Bergey's Manual of Systematic Bacteriology, Vol. 3-5, Edn. 2nd.	n/a	n/a	S4
Lactobacillus sp. OTU_19	n/a	n/a	USES	oxygen	metabolite	HMDB0001377	https://hmdb.ca/metabolites/HMDB0001377	n/a	n/a	prediction	Prediction based on the characteristics of related L. paracasei. Data retrived from review and not original research article.	Bergey's Manual of Systematic Bacteriology, Vol. 3-5, Edn. 2nd.	n/a	n/a	S4
Lactobacillus sp. OTU_6	n/a	n/a	IS_INHIBITED_BY	oxygen	metabolite	HMDB0001377	https://hmdb.ca/metabolites/HMDB0001377	n/a	n/a	prediction	Prediction based on the characteristics of related L. pentosus. Data retrived from review and not original research article. Variable	Bergey's Manual of Systematic Bacteriology, Vol. 3-5, Edn. 2nd.	n/a	n/a	S4
Lactobacillus sp. OTU_6	n/a	n/a	PRODUCES	ammonia	metabolite	HMDB0000051	https://hmdb.ca/metabolites/HMDB0000051	n/a	n/a	prediction	Prediction based on the characteristics of related L. pentosus. Data retrived from review and not original research article.	Bergey's Manual of Systematic Bacteriology, Vol. 3-5, Edn. 2nd.	n/a	n/a	S4
Lactobacillus sp. OTU_6	n/a	n/a	PRODUCES	catechol	metabolite	n/a	n/a	n/a	n/a	prediction	Prediction based on the characteristics of related L. pentosus. Data retrived from review and not original research article.	Bergey's Manual of Systematic Bacteriology, Vol. 3-5, Edn. 2nd.	n/a	n/a	S4
Lactobacillus sp. OTU_6	n/a	n/a	PRODUCES	lactic acid	metabolite	HMDB0000190	https://hmdb.ca/metabolites/HMDB0000190	n/a	n/a	prediction	Prediction based on the characteristics of related L. pentosus. Data retrived from review and not original research article.	Bergey's Manual of Systematic Bacteriology, Vol. 3-5, Edn. 2nd.	n/a	n/a	S4
Lactobacillus sp. OTU_6	n/a	n/a	USES	carbon dioxide	metabolite	HMDB0001967	https://hmdb.ca/metabolites/HMDB0001967	n/a	n/a	prediction	Prediction based on the characteristics of related L. pentosus. Data retrived from review and not original research article.	Bergey's Manual of Systematic Bacteriology, Vol. 3-5, Edn. 2nd.	n/a	n/a	S4
Lactobacillus sp. OTU_6	n/a	n/a	USES	lactic acid	metabolite	HMDB0000190	https://hmdb.ca/metabolites/HMDB0000190	n/a	n/a	prediction	Prediction based on the characteristics of related L. pentosus. Data retrived from review and not original research article.	Bergey's Manual of Systematic Bacteriology, Vol. 3-5, Edn. 2nd.	n/a	n/a	S4
Lactobacillus sp. OTU_6	n/a	n/a	USES	nitrite	metabolite	HMDB0002786	https://hmdb.ca/metabolites/HMDB0002786	n/a	n/a	prediction	Prediction based on the characteristics of related L. pentosus. Data retrived from review and not original research article. Variable	Bergey's Manual of Systematic Bacteriology, Vol. 3-5, Edn. 2nd.	n/a	n/a	S4
Lactobacillus sp. OTU_6	n/a	n/a	USES	oxygen	metabolite	HMDB0001377	https://hmdb.ca/metabolites/HMDB0001377	n/a	n/a	prediction	Prediction based on the characteristics of related L. pentosus. Data retrived from review and not original research article. Variable	Bergey's Manual of Systematic Bacteriology, Vol. 3-5, Edn. 2nd.	n/a	n/a	S4
Lactobacillus sp. OTU_6	n/a	n/a	USES	shikimic acid	metabolite	HMDB0003070	https://hmdb.ca/metabolites/HMDB0003070	n/a	n/a	prediction	Prediction based on the characteristics of related L. pentosus. Data retrived from review and not original research article. Variable	Bergey's Manual of Systematic Bacteriology, Vol. 3-5, Edn. 2nd.	n/a	n/a	S4
Lancefieldella parvulae	723	https://www.homd.org/taxa/tax_description?otid=723	IS_INHIBITED_BY	oxygen	metabolite	HMDB0001377	https://hmdb.ca/metabolites/HMDB0001377	n/a	n/a	experimental	Data retrived from review and not original research article.	Bergey's Manual of Systematic Bacteriology, Vol. 3-5, Edn. 2nd.	n/a	n/a	S4
Lancefieldella parvulae	723	https://www.homd.org/taxa/tax_description?otid=723	PRODUCES	lactic acid	metabolite	HMDB0000190	https://hmdb.ca/metabolites/HMDB0000190	n/a	n/a	experimental	Data retrived from review and not original research article.	Bergey's Manual of Systematic Bacteriology, Vol. 3-5, Edn. 2nd.	n/a	n/a	S4
Lancefieldella rimae	750	https://www.homd.org/taxa/tax_description?otid=750	IS_INHIBITED_BY	oxygen	metabolite	HMDB0001377	https://hmdb.ca/metabolites/HMDB0001377	n/a	n/a	experimental	Data retrived from review and not original research article.	Bergey's Manual of Systematic Bacteriology, Vol. 3-5, Edn. 2nd.	n/a	n/a	S4
Lancefieldella rimae	750	https://www.homd.org/taxa/tax_description?otid=750	PRODUCES	lactic acid	metabolite	HMDB0000190	https://hmdb.ca/metabolites/HMDB0000190	n/a	n/a	experimental	Data retrived from review and not original research article.	Bergey's Manual of Systematic Bacteriology, Vol. 3-5, Edn. 2nd.	n/a	n/a	S4
Lautropia mirabilis	22	https://www.homd.org/taxa/tax_description?otid=022	DEGRADES	hydrogen peroxide	metabolite	HMDB0003125	https://hmdb.ca/metabolites/HMDB0003125	n/a	n/a	experimental		GernerSmidt1994 10.1099/13500872-140-7-1787	https://pubmed.ncbi.nlm.nih.gov/8075812/	n/a	7c
Lautropia mirabilis	22	https://www.homd.org/taxa/tax_description?otid=022	PRODUCES	acid	metabolite	n/a	n/a	n/a	n/a	experimental		GernerSmidt1994 10.1099/13500872-140-7-1787	https://pubmed.ncbi.nlm.nih.gov/8075812/	n/a	7c
Lautropia mirabilis	22	https://www.homd.org/taxa/tax_description?otid=022	PRODUCES	ammonia	metabolite	HMDB0000051	https://hmdb.ca/metabolites/HMDB0000051	n/a	n/a	experimental		GernerSmidt1994 10.1099/13500872-140-7-1787	https://pubmed.ncbi.nlm.nih.gov/8075812/	n/a	7c
Lautropia mirabilis	22	https://www.homd.org/taxa/tax_description?otid=022	PRODUCES	beta-xylosidase	enzyme	n/a	n/a	3.2.1.21	https://www.genome.jp/entry/3.2.1.21	experimental		GernerSmidt1994 10.1099/13500872-140-7-1787	https://pubmed.ncbi.nlm.nih.gov/8075812/	n/a	7c
Lautropia mirabilis	22	https://www.homd.org/taxa/tax_description?otid=022	PRODUCES	capsule	cell component	n/a	n/a	n/a	n/a	experimental		GernerSmidt1994 10.1099/13500872-140-7-1787	https://pubmed.ncbi.nlm.nih.gov/8075812/	n/a	7c
Lautropia mirabilis	22	https://www.homd.org/taxa/tax_description?otid=022	PRODUCES	catalase	enzyme	n/a	n/a	1.11.1.6	https://www.genome.jp/entry/1.11.1.6	experimental	Weakly catalase positive.	GernerSmidt1994 10.1099/13500872-140-7-1787	https://pubmed.ncbi.nlm.nih.gov/8075812/	n/a	7c
Lautropia mirabilis	22	https://www.homd.org/taxa/tax_description?otid=022	PRODUCES	cell aggregates	biofilm component	n/a	n/a	n/a	n/a	experimental	Often occurring in clusters of 10 to 100 cells.	GernerSmidt1994 10.1099/13500872-140-7-1787	https://pubmed.ncbi.nlm.nih.gov/8075812/	n/a	7c
Lautropia mirabilis	22	https://www.homd.org/taxa/tax_description?otid=022	PRODUCES	enzyme generating polysacharide from sucorse	enzyme	n/a	n/a	n/a	n/a	experimental		GernerSmidt1994 10.1099/13500872-140-7-1787	https://pubmed.ncbi.nlm.nih.gov/8075812/	n/a	7c
Lautropia mirabilis	22	https://www.homd.org/taxa/tax_description?otid=022	PRODUCES	flagella	cell component	n/a	n/a	n/a	n/a	experimental		GernerSmidt1994 10.1099/13500872-140-7-1787	https://pubmed.ncbi.nlm.nih.gov/8075812/	n/a	7c
Lautropia mirabilis	22	https://www.homd.org/taxa/tax_description?otid=022	PRODUCES	nitrate reductase	enzyme	n/a	n/a	n/a	n/a	experimental		GernerSmidt1994 10.1099/13500872-140-7-1787	https://pubmed.ncbi.nlm.nih.gov/8075812/	n/a	7c
Lautropia mirabilis	22	https://www.homd.org/taxa/tax_description?otid=022	PRODUCES	nitrite	metabolite	HMDB0002786	https://hmdb.ca/metabolites/HMDB0002786	n/a	n/a	experimental		GernerSmidt1994 10.1099/13500872-140-7-1787	https://pubmed.ncbi.nlm.nih.gov/8075812/	n/a	7c
Lautropia mirabilis	22	https://www.homd.org/taxa/tax_description?otid=022	PRODUCES	nitrite reductase	enzyme	n/a	n/a	n/a	n/a	experimental		GernerSmidt1994 10.1099/13500872-140-7-1787	https://pubmed.ncbi.nlm.nih.gov/8075812/	n/a	7c
Lautropia mirabilis	22	https://www.homd.org/taxa/tax_description?otid=022	PRODUCES	oxidase	enzyme	n/a	n/a	n/a	n/a	experimental		GernerSmidt1994 10.1099/13500872-140-7-1787	https://pubmed.ncbi.nlm.nih.gov/8075812/	n/a	7c
Lautropia mirabilis	22	https://www.homd.org/taxa/tax_description?otid=022	PRODUCES	polysacharide	biofilm component	n/a	n/a	n/a	n/a	experimental		GernerSmidt1994 10.1099/13500872-140-7-1787	https://pubmed.ncbi.nlm.nih.gov/8075812/	n/a	7c
Lautropia mirabilis	22	https://www.homd.org/taxa/tax_description?otid=022	PRODUCES	urease	enzyme	n/a	n/a	3.5.1.5	https://www.genome.jp/entry/3.5.1.5	experimental		GernerSmidt1994 10.1099/13500872-140-7-1787	https://pubmed.ncbi.nlm.nih.gov/8075812/	n/a	7c
Lautropia mirabilis	22	https://www.homd.org/taxa/tax_description?otid=022	USES	D-fructose	metabolite	HMDB0000660	https://hmdb.ca/metabolites/HMDB0000660	n/a	n/a	experimental	Fermentation.	GernerSmidt1994 10.1099/13500872-140-7-1787	https://pubmed.ncbi.nlm.nih.gov/8075812/	n/a	7c
Lautropia mirabilis	22	https://www.homd.org/taxa/tax_description?otid=022	USES	D-galactose	metabolite	HMDB0000143	https://hmdb.ca/metabolites/HMDB0000143	n/a	n/a	experimental	Fermentation.	GernerSmidt1994 10.1099/13500872-140-7-1787	https://pubmed.ncbi.nlm.nih.gov/8075812/	n/a	7c
Lautropia mirabilis	22	https://www.homd.org/taxa/tax_description?otid=022	USES	D-glucose	metabolite	HMDB0000122	https://hmdb.ca/metabolites/HMDB0000122	n/a	n/a	experimental	Fermentation.	GernerSmidt1994 10.1099/13500872-140-7-1787	https://pubmed.ncbi.nlm.nih.gov/8075812/	n/a	7c
Lautropia mirabilis	22	https://www.homd.org/taxa/tax_description?otid=022	USES	D-maltose	metabolite	HMDB0000163	https://hmdb.ca/metabolites/HMDB0000163	n/a	n/a	experimental	Fermentation.	GernerSmidt1994 10.1099/13500872-140-7-1787	https://pubmed.ncbi.nlm.nih.gov/8075812/	n/a	7c
Lautropia mirabilis	22	https://www.homd.org/taxa/tax_description?otid=022	USES	growth factors	metabolite	n/a	n/a	n/a	n/a	experimental	Generally regarded as a fastidious organisms which can be hard to culture in vitro.	GernerSmidt1994 10.1099/13500872-140-7-1787	https://pubmed.ncbi.nlm.nih.gov/8075812/	n/a	7c
Lautropia mirabilis	22	https://www.homd.org/taxa/tax_description?otid=022	USES	mannitol	metabolite	HMDB0000765	https://hmdb.ca/metabolites/HMDB0000765	n/a	n/a	experimental	Fermentation.	GernerSmidt1994 10.1099/13500872-140-7-1787	https://pubmed.ncbi.nlm.nih.gov/8075812/	n/a	7c
Lautropia mirabilis	22	https://www.homd.org/taxa/tax_description?otid=022	USES	nitrate	metabolite	HMDB0002878	https://hmdb.ca/metabolites/HMDB0002878	n/a	n/a	experimental		GernerSmidt1994 10.1099/13500872-140-7-1787	https://pubmed.ncbi.nlm.nih.gov/8075812/	n/a	7c
Lautropia mirabilis	22	https://www.homd.org/taxa/tax_description?otid=022	USES	nitrite	metabolite	HMDB0002786	https://hmdb.ca/metabolites/HMDB0002786	n/a	n/a	experimental		GernerSmidt1994 10.1099/13500872-140-7-1787	https://pubmed.ncbi.nlm.nih.gov/8075812/	n/a	7c
Lautropia mirabilis	22	https://www.homd.org/taxa/tax_description?otid=022	USES	oxygen	metabolite	HMDB0001377	https://hmdb.ca/metabolites/HMDB0001377	n/a	n/a	experimental	Grows best under aerobic conditions.	GernerSmidt1994 10.1099/13500872-140-7-1787	https://pubmed.ncbi.nlm.nih.gov/8075812/	n/a	7c
Lautropia mirabilis	22	https://www.homd.org/taxa/tax_description?otid=022	USES	sucrose	metabolite	HMDB0000258	https://hmdb.ca/metabolites/HMDB0000258	n/a	n/a	experimental		GernerSmidt1994 10.1099/13500872-140-7-1787	https://pubmed.ncbi.nlm.nih.gov/8075812/	n/a	7c
Lautropia mirabilis	22	https://www.homd.org/taxa/tax_description?otid=022	USES	urea	metabolite	HMDB0000294	https://hmdb.ca/metabolites/HMDB0000294	n/a	n/a	experimental		GernerSmidt1994 10.1099/13500872-140-7-1787	https://pubmed.ncbi.nlm.nih.gov/8075812/	n/a	7c
Limosilactobacillus fermentum	608	https://www.homd.org/taxa/tax_description?otid=608	IS_INHIBITED_BY	oxygen	metabolite	HMDB0001377	https://hmdb.ca/metabolites/HMDB0001377	n/a	n/a	experimental	Data retrived from review and not original research article.	Bergey's Manual of Systematic Bacteriology, Vol. 3-5, Edn. 2nd.	n/a	n/a	S4
Limosilactobacillus fermentum	608	https://www.homd.org/taxa/tax_description?otid=608	PRODUCES	lactic acid	metabolite	HMDB0000190	https://hmdb.ca/metabolites/HMDB0000190	n/a	n/a	experimental	Data retrived from review and not original research article.	Bergey's Manual of Systematic Bacteriology, Vol. 3-5, Edn. 2nd.	n/a	n/a	S4
Limosilactobacillus fermentum	608	https://www.homd.org/taxa/tax_description?otid=608	USES	carbon dioxide	metabolite	HMDB0001967	https://hmdb.ca/metabolites/HMDB0001967	n/a	n/a	experimental	Data retrived from review and not original research article.	Bergey's Manual of Systematic Bacteriology, Vol. 3-5, Edn. 2nd.	n/a	n/a	S4
Limosilactobacillus fermentum	608	https://www.homd.org/taxa/tax_description?otid=608	USES	L-arginine	metabolite	HMDB0000517	https://hmdb.ca/metabolites/HMDB0000517	n/a	n/a	experimental	Data retrived from review and not original research article.	Bergey's Manual of Systematic Bacteriology, Vol. 3-5, Edn. 2nd.	n/a	n/a	S4
Limosilactobacillus fermentum	608	https://www.homd.org/taxa/tax_description?otid=608	USES	oxygen	metabolite	HMDB0001377	https://hmdb.ca/metabolites/HMDB0001377	n/a	n/a	experimental	Data retrived from review and not original research article.	Bergey's Manual of Systematic Bacteriology, Vol. 3-5, Edn. 2nd.	n/a	n/a	S4
Limosilactobacillus fermentum	608	https://www.homd.org/taxa/tax_description?otid=608	USES	pantothenic acid	metabolite	HMDB0000210	https://hmdb.ca/metabolites/HMDB0000210	n/a	n/a	experimental	Data retrived from review and not original research article.	Bergey's Manual of Systematic Bacteriology, Vol. 3-5, Edn. 2nd.	n/a	n/a	S4
Limosilactobacillus fermentum	608	https://www.homd.org/taxa/tax_description?otid=608	USES	thiamine	metabolite	HMDB0000235	https://hmdb.ca/metabolites/HMDB0000235	n/a	n/a	experimental	Data retrived from review and not original research article.	Bergey's Manual of Systematic Bacteriology, Vol. 3-5, Edn. 2nd.	n/a	n/a	S4
Mogibacterium sp. OTU_21	n/a	n/a	IS_INHIBITED_BY	oxygen	metabolite	HMDB0001377	https://hmdb.ca/metabolites/HMDB0001377	n/a	n/a	prediction	Prediction based on the characteristics of Mogibacterium genus. Data retrived from review and not original research article.	Bergey's Manual of Systematic Bacteriology, Vol. 3-5, Edn. 2nd.	n/a	n/a	S4
Mogibacterium sp. OTU_21	n/a	n/a	PRODUCES	phenylacetic acid	metabolite	HMDB0000209	https://hmdb.ca/metabolites/HMDB0000209	n/a	n/a	prediction	Prediction based on the characteristics of Mogibacterium genus. Data retrived from review and not original research article.	Bergey's Manual of Systematic Bacteriology, Vol. 3-5, Edn. 2nd.	n/a	n/a	S4
Neisseria sicca	764	https://www.homd.org/taxa/tax_description?otid=764	PRODUCES	catalase	enzyme	n/a	n/a	1.11.1.6	https://www.genome.jp/entry/1.11.1.6	experimental	Data retrived from review and not original research article.	Bergey's Manual of Systematic Bacteriology, Vol. 3-5, Edn. 2nd.	n/a	n/a	S4
Neisseria sicca	764	https://www.homd.org/taxa/tax_description?otid=764	PRODUCES	nitric oxide	metabolite	HMDB0003378	https://hmdb.ca/metabolites/HMDB0003378	n/a	n/a	experimental	Data retrived from review and not original research article.	Bergey's Manual of Systematic Bacteriology, Vol. 3-5, Edn. 2nd.	n/a	n/a	S4
Neisseria sicca	764	https://www.homd.org/taxa/tax_description?otid=764	USES	nitrite	metabolite	HMDB0002786	https://hmdb.ca/metabolites/HMDB0002786	n/a	n/a	experimental	Data retrived from review and not original research article.	Bergey's Manual of Systematic Bacteriology, Vol. 3-5, Edn. 2nd.	n/a	n/a	S4
Neisseria sicca	764	https://www.homd.org/taxa/tax_description?otid=764	USES	oxygen	metabolite	HMDB0001377	https://hmdb.ca/metabolites/HMDB0001377	n/a	n/a	experimental	Data retrived from review and not original research article.	Bergey's Manual of Systematic Bacteriology, Vol. 3-5, Edn. 2nd.	n/a	n/a	S4
Neisseria sp. OTU_4	n/a	n/a	PRODUCES	catalase	enzyme	n/a	n/a	1.11.1.6	https://www.genome.jp/entry/1.11.1.6	prediction	Prediction based on the characteristics of related N. mucosa/sicca	Bergey's Manual of Systematic Bacteriology, Vol. 3-5, Edn. 2nd.	n/a	n/a	S4
Neisseria sp. OTU_4	n/a	n/a	PRODUCES	nitric oxide	metabolite	HMDB0003378	https://hmdb.ca/metabolites/HMDB0003378	n/a	n/a	prediction	Prediction based on the characteristics of related N. mucosa/sicca	Bergey's Manual of Systematic Bacteriology, Vol. 3-5, Edn. 2nd.	n/a	n/a	S4
Neisseria sp. OTU_4	n/a	n/a	PRODUCES	nitrite	metabolite	HMDB0002786	https://hmdb.ca/metabolites/HMDB0002786	n/a	n/a	prediction	Prediction based on the characteristics of related N. mucosa/sicca	Bergey's Manual of Systematic Bacteriology, Vol. 3-5, Edn. 2nd.	n/a	n/a	S4
Neisseria sp. OTU_4	n/a	n/a	USES	nitrate	metabolite	HMDB0002878	https://hmdb.ca/metabolites/HMDB0002878	n/a	n/a	prediction	Prediction based on the characteristics of related N. mucosa/sicca	Bergey's Manual of Systematic Bacteriology, Vol. 3-5, Edn. 2nd.	n/a	n/a	S4
Neisseria sp. OTU_4	n/a	n/a	USES	nitrite	metabolite	HMDB0002786	https://hmdb.ca/metabolites/HMDB0002786	n/a	n/a	prediction	Prediction based on the characteristics of related N. mucosa/sicca	Bergey's Manual of Systematic Bacteriology, Vol. 3-5, Edn. 2nd.	n/a	n/a	S4
Neisseria sp. OTU_4	n/a	n/a	USES	oxygen	metabolite	HMDB0001377	https://hmdb.ca/metabolites/HMDB0001377	n/a	n/a	prediction	Prediction based on the characteristics of related N. mucosa/sicca	Bergey's Manual of Systematic Bacteriology, Vol. 3-5, Edn. 2nd.	n/a	n/a	S4
Porphyromonas pasteri	279	https://www.homd.org/taxa/tax_description?otid=279	IS_INHIBITED_BY	hydrogen peroxide	metabolite	HMDB0003125	https://hmdb.ca/metabolites/HMDB0003125	n/a	n/a	experimental		Sakamoto2015 10.1099/ijs.0.000294	https://pubmed.ncbi.nlm.nih.gov/25933621/	n/a	7c
Porphyromonas pasteri	279	https://www.homd.org/taxa/tax_description?otid=279	IS_INHIBITED_BY	oxygen	metabolite	HMDB0001377	https://hmdb.ca/metabolites/HMDB0001377	n/a	n/a	prediction	Cells reported as obligately anaerobic. Aerobic respiration and microaerophilic growth potentially possible - prediction based on the characteristics of related P. gingivalis species.	Sakamoto2015 10.1099/ijs.0.000294	https://pubmed.ncbi.nlm.nih.gov/25933621/	n/a	7c
Porphyromonas pasteri	279	https://www.homd.org/taxa/tax_description?otid=279	PRODUCES	acid phosphatase	enzyme	n/a	n/a	3.1.3.2	https://www.genome.jp/entry/3.1.3.2	experimental	Positive reactions were obtained using API ZYM.	Sakamoto2015 10.1099/ijs.0.000294	https://pubmed.ncbi.nlm.nih.gov/25933621/	n/a	7c
Porphyromonas pasteri	279	https://www.homd.org/taxa/tax_description?otid=279	PRODUCES	alanine arylamidase	enzyme	n/a	n/a	3.4.11.14	https://www.genome.jp/entry/3.4.11.14	experimental	Positive reactions were obtained using Rapid ID 32A.	Sakamoto2015 10.1099/ijs.0.000294	https://pubmed.ncbi.nlm.nih.gov/25933621/	n/a	7c
Porphyromonas pasteri	279	https://www.homd.org/taxa/tax_description?otid=279	PRODUCES	alkaline phosphatase	enzyme	n/a	n/a	3.1.3.1	https://www.genome.jp/entry/3.1.3.1	experimental	Positive reactions were obtained using Rapid ID 32A and API ZYM.	Sakamoto2015 10.1099/ijs.0.000294	https://pubmed.ncbi.nlm.nih.gov/25933621/	n/a	7c
Porphyromonas pasteri	279	https://www.homd.org/taxa/tax_description?otid=279	PRODUCES	alpha-fucosidase	enzyme	n/a	n/a	3.2.1.51	https://www.genome.jp/entry/3.2.1.51	experimental	Positive reactions were obtained using Rapid ID 32A and API ZYM.	Sakamoto2015 10.1099/ijs.0.000294	https://pubmed.ncbi.nlm.nih.gov/25933621/	n/a	7c
Porphyromonas pasteri	279	https://www.homd.org/taxa/tax_description?otid=279	PRODUCES	alpha-glucosidase	enzyme	n/a	n/a	3.2.1.20	https://www.genome.jp/entry/3.2.1.20	experimental	Positive reactions were obtained using Rapid ID 32A and API ZYM (weak).	Sakamoto2015 10.1099/ijs.0.000294	https://pubmed.ncbi.nlm.nih.gov/25933621/	n/a	7c
Porphyromonas pasteri	279	https://www.homd.org/taxa/tax_description?otid=279	PRODUCES	beta-galactosidase	enzyme	n/a	n/a	3.2.1.23	https://www.genome.jp/entry/3.2.1.23	experimental	Positive reactions were obtained using Rapid ID 32A and API ZYM (weak).	Sakamoto2015 10.1099/ijs.0.000294	https://pubmed.ncbi.nlm.nih.gov/25933621/	n/a	7c
Porphyromonas pasteri	279	https://www.homd.org/taxa/tax_description?otid=279	PRODUCES	enzyme generating glutamate from ammonia	enzyme	n/a	n/a	n/a	n/a	experimental		OFlynn2015 10.1093/gbe/evv220	https://pubmed.ncbi.nlm.nih.gov/26568374/	n/a	7c
Porphyromonas pasteri	279	https://www.homd.org/taxa/tax_description?otid=279	PRODUCES	enzyme generating vitamin B12 from (proto)porphyrin	enzyme	n/a	n/a	n/a	n/a	experimental		OFlynn2015 10.1093/gbe/evv220	https://pubmed.ncbi.nlm.nih.gov/26568374/	n/a	7c
Porphyromonas pasteri	279	https://www.homd.org/taxa/tax_description?otid=279	PRODUCES	esterase	enzyme	n/a	n/a	n/a	n/a	experimental	Positive reactions were obtained using API ZYM. Weak.	Sakamoto2015 10.1099/ijs.0.000294	https://pubmed.ncbi.nlm.nih.gov/25933621/	n/a	7c
Porphyromonas pasteri	279	https://www.homd.org/taxa/tax_description?otid=279	PRODUCES	glutamyl glutamic acid arylamidase	enzyme	n/a	n/a	n/a	n/a	experimental	Positive reactions were obtained using Rapid ID 32A.	Sakamoto2015 10.1099/ijs.0.000294	https://pubmed.ncbi.nlm.nih.gov/25933621/	n/a	7c
Porphyromonas pasteri	279	https://www.homd.org/taxa/tax_description?otid=279	PRODUCES	leucine arylamidase	enzyme	n/a	n/a	n/a	n/a	experimental	Positive reactions were obtained using API ZYM. Weak.	Sakamoto2015 10.1099/ijs.0.000294	https://pubmed.ncbi.nlm.nih.gov/25933621/	n/a	7c
Porphyromonas pasteri	279	https://www.homd.org/taxa/tax_description?otid=279	PRODUCES	leucyl glycine arylamidase	enzyme	n/a	n/a	n/a	n/a	experimental	Positive reactions were obtained using Rapid ID 32A.	Sakamoto2015 10.1099/ijs.0.000294	https://pubmed.ncbi.nlm.nih.gov/25933621/	n/a	7c
Porphyromonas pasteri	279	https://www.homd.org/taxa/tax_description?otid=279	PRODUCES	N-acetyl-beta-glucosaminidase	enzyme	n/a	n/a	3.2.1.52	https://www.genome.jp/entry/3.2.1.52	experimental	Positive reactions were obtained using Rapid ID 32A and API ZYM.	Sakamoto2015 10.1099/ijs.0.000294	https://pubmed.ncbi.nlm.nih.gov/25933621/	n/a	7c
Porphyromonas pasteri	279	https://www.homd.org/taxa/tax_description?otid=279	PRODUCES	naphthol-AS-BI-phosphohydrolase	enzyme	n/a	n/a	n/a	n/a	experimental	Positive reactions were obtained using API ZYM.	Sakamoto2015 10.1099/ijs.0.000294	https://pubmed.ncbi.nlm.nih.gov/25933621/	n/a	7c
Porphyromonas pasteri	279	https://www.homd.org/taxa/tax_description?otid=279	PRODUCES	propionic acid	metabolite	HMDB0000237	https://hmdb.ca/metabolites/HMDB0000237	n/a	n/a	experimental		Sakamoto2015 10.1099/ijs.0.000294	https://pubmed.ncbi.nlm.nih.gov/25933621/	n/a	7c
Porphyromonas pasteri	279	https://www.homd.org/taxa/tax_description?otid=279	USES	(proto)porphyrin	metabolite	n/a	n/a	n/a	n/a	experimental		Sakamoto2015 10.1099/ijs.0.000294	https://pubmed.ncbi.nlm.nih.gov/25933621/	n/a	7c
Porphyromonas pasteri	279	https://www.homd.org/taxa/tax_description?otid=279	USES	alpha-lactose	metabolite	HMDB0000186	https://hmdb.ca/metabolites/HMDB0000186	n/a	n/a	experimental		Sakamoto2015 10.1099/ijs.0.000294	https://pubmed.ncbi.nlm.nih.gov/25933621/	n/a	7c
Porphyromonas pasteri	279	https://www.homd.org/taxa/tax_description?otid=279	USES	D-glucose	metabolite	HMDB0000122	https://hmdb.ca/metabolites/HMDB0000122	n/a	n/a	experimental		Sakamoto2015 10.1099/ijs.0.000294	https://pubmed.ncbi.nlm.nih.gov/25933621/	n/a	7c
Porphyromonas pasteri	279	https://www.homd.org/taxa/tax_description?otid=279	USES	DHNA	metabolite	HMDB0244212	https://hmdb.ca/metabolites/HMDB0244212	n/a	n/a	experimental		Sakamoto2015 10.1099/ijs.0.000294	https://pubmed.ncbi.nlm.nih.gov/25933621/	n/a	7c
Porphyromonas pasteri	279	https://www.homd.org/taxa/tax_description?otid=279	USES	D-maltose	metabolite	HMDB0000163	https://hmdb.ca/metabolites/HMDB0000163	n/a	n/a	experimental		Sakamoto2015 10.1099/ijs.0.000294	https://pubmed.ncbi.nlm.nih.gov/25933621/	n/a	7c
Porphyromonas pasteri	279	https://www.homd.org/taxa/tax_description?otid=279	USES	D-mannose	metabolite	HMDB0000169	https://hmdb.ca/metabolites/HMDB0000169	n/a	n/a	experimental		Sakamoto2015 10.1099/ijs.0.000294	https://pubmed.ncbi.nlm.nih.gov/25933621/	n/a	7c
Porphyromonas pasteri	279	https://www.homd.org/taxa/tax_description?otid=279	USES	growth factors	metabolite	n/a	n/a	n/a	n/a	experimental	Generally regarded as a fastidious organisms which can be hard to culture in vitro.	Sakamoto2015 10.1099/ijs.0.000294	https://pubmed.ncbi.nlm.nih.gov/25933621/	n/a	7c
Porphyromonas pasteri	279	https://www.homd.org/taxa/tax_description?otid=279	USES	hemin	metabolite	HMDB0000887	https://hmdb.ca/metabolites/HMDB0000887	n/a	n/a	experimental	Added hemin, blood, or hemoglobin fulfilled the heme requirement for growth. The heme requirement results from an incomplete heme biosynthetic pathway in P. pasteri.	Murugkar2022 10.1080/20002297.2022.2143651	https://pubmed.ncbi.nlm.nih.gov/36452178/	n/a	7c
Porphyromonas pasteri	279	https://www.homd.org/taxa/tax_description?otid=279	USES	iron	metabolite	n/a	n/a	n/a	n/a	experimental		Sakamoto2015 10.1099/ijs.0.000294	https://pubmed.ncbi.nlm.nih.gov/25933621/	n/a	7c
Porphyromonas pasteri	279	https://www.homd.org/taxa/tax_description?otid=279	USES	lactic acid	metabolite	HMDB0000190	https://hmdb.ca/metabolites/HMDB0000190	n/a	n/a	prediction	Prediction based on the characteristics of related P. gingivalis species.	Lewis2009 10.1099/mic.0.027953-0	https://pubmed.ncbi.nlm.nih.gov/19684063/	n/a	7c
Porphyromonas pasteri	279	https://www.homd.org/taxa/tax_description?otid=279	USES	menaquinone	metabolite	n/a	n/a	n/a	n/a	experimental	Analysis of the E. coli deletion mutant library and growth studies identified 1,2-dihydroxy-2-naphthoic acid (DHNA) and menaquinone-4 (MK4) as the growth factors.	Murugkar2022 10.1080/20002297.2022.2143651	https://pubmed.ncbi.nlm.nih.gov/36452178/	n/a	7c
Porphyromonas pasteri	279	https://www.homd.org/taxa/tax_description?otid=279	USES	sucrose	metabolite	HMDB0000258	https://hmdb.ca/metabolites/HMDB0000258	n/a	n/a	experimental	Fermentation.	Sakamoto2015 10.1099/ijs.0.000294	https://pubmed.ncbi.nlm.nih.gov/25933621/	n/a	7c
Prevotella histicola	298	https://www.homd.org/taxa/tax_description?otid=298	IS_INHIBITED_BY	oxygen	metabolite	HMDB0001377	https://hmdb.ca/metabolites/HMDB0001377	n/a	n/a	prediction	Prediction based on the characteristics of Prevotella genus. Data retrived from review and not original research article.	Bergey's Manual of Systematic Bacteriology, Vol. 3-5, Edn. 2nd.	n/a	n/a	S4
Prevotella histicola	298	https://www.homd.org/taxa/tax_description?otid=298	USES	hemin	metabolite	HMDB0000887	https://hmdb.ca/metabolites/HMDB0000887	n/a	n/a	prediction	Prediction based on the characteristics of Prevotella genus. Data retrived from review and not original research article.	Bergey's Manual of Systematic Bacteriology, Vol. 3-5, Edn. 2nd.	n/a	n/a	S4
Prevotella nanceiensis	299	https://www.homd.org/taxa/tax_description?otid=299	IS_INHIBITED_BY	oxygen	metabolite	HMDB0001377	https://hmdb.ca/metabolites/HMDB0001377	n/a	n/a	prediction	Prediction based on the characteristics of Prevotella genus. Data retrived from review and not original research article.	Bergey's Manual of Systematic Bacteriology, Vol. 3-5, Edn. 2nd.	n/a	n/a	S4
Prevotella nanceiensis	299	https://www.homd.org/taxa/tax_description?otid=299	PRODUCES	lactic acid	metabolite	HMDB0000190	https://hmdb.ca/metabolites/HMDB0000190	n/a	n/a	experimental	Data retrived from review and not original research article.	Bergey's Manual of Systematic Bacteriology, Vol. 3-5, Edn. 2nd.	n/a	n/a	S4
Prevotella nanceiensis	299	https://www.homd.org/taxa/tax_description?otid=299	USES	hemin	metabolite	HMDB0000887	https://hmdb.ca/metabolites/HMDB0000887	n/a	n/a	prediction	Prediction based on the characteristics of Prevotella genus. Data retrived from review and not original research article.	Bergey's Manual of Systematic Bacteriology, Vol. 3-5, Edn. 2nd.	n/a	n/a	S4
Prevotella salivae	307	https://www.homd.org/taxa/tax_description?otid=307	IS_INHIBITED_BY	oxygen	metabolite	HMDB0001377	https://hmdb.ca/metabolites/HMDB0001377	n/a	n/a	experimental	Data retrived from review and not original research article.	Bergey's Manual of Systematic Bacteriology, Vol. 3-5, Edn. 2nd.	n/a	n/a	S4
Prevotella salivae	307	https://www.homd.org/taxa/tax_description?otid=307	USES	hemin	metabolite	HMDB0000887	https://hmdb.ca/metabolites/HMDB0000887	n/a	n/a	prediction	Prediction based on the characteristics of Prevotella genus. Data retrived from review and not original research article.	Bergey's Manual of Systematic Bacteriology, Vol. 3-5, Edn. 2nd.	n/a	n/a	S4
Rothia dentocariosa	587	https://www.homd.org/taxa/tax_description?otid=587	IS_INHIBITED_BY	hydrogen peroxide	metabolite	HMDB0003125	https://hmdb.ca/metabolites/HMDB0003125	n/a	n/a	experimental	Data retrived from review and not original research article.	Bergey's Manual of Systematic Bacteriology, Vol. 3-5, Edn. 2nd.	n/a	n/a	S4
Rothia dentocariosa	587	https://www.homd.org/taxa/tax_description?otid=587	PRODUCES	catalase	enzyme	n/a	n/a	1.11.1.6	https://www.genome.jp/entry/1.11.1.6	experimental	Data retrived from review and not original research article. Variable.	Bergey's Manual of Systematic Bacteriology, Vol. 3-5, Edn. 2nd.	n/a	n/a	S4
Rothia dentocariosa	587	https://www.homd.org/taxa/tax_description?otid=587	PRODUCES	lactic acid	metabolite	HMDB0000190	https://hmdb.ca/metabolites/HMDB0000190	n/a	n/a	experimental	Data retrived from review and not original research article.	Bergey's Manual of Systematic Bacteriology, Vol. 3-5, Edn. 2nd.	n/a	n/a	S4
Rothia dentocariosa	587	https://www.homd.org/taxa/tax_description?otid=587	PRODUCES	menaquinone	metabolite	n/a	n/a	n/a	n/a	experimental	Data retrived from review and not original research article.	Bergey's Manual of Systematic Bacteriology, Vol. 3-5, Edn. 2nd.	n/a	n/a	S4
Rothia dentocariosa	587	https://www.homd.org/taxa/tax_description?otid=587	PRODUCES	nitrite	metabolite	HMDB0002786	https://hmdb.ca/metabolites/HMDB0002786	n/a	n/a	experimental	Data retrived from review and not original research article.	Bergey's Manual of Systematic Bacteriology, Vol. 3-5, Edn. 2nd.	n/a	n/a	S4
Rothia dentocariosa	587	https://www.homd.org/taxa/tax_description?otid=587	USES	nitrate	metabolite	HMDB0002878	https://hmdb.ca/metabolites/HMDB0002878	n/a	n/a	experimental	Data retrived from review and not original research article.	Bergey's Manual of Systematic Bacteriology, Vol. 3-5, Edn. 2nd.	n/a	n/a	S4
Rothia dentocariosa	587	https://www.homd.org/taxa/tax_description?otid=587	USES	oxygen	metabolite	HMDB0001377	https://hmdb.ca/metabolites/HMDB0001377	n/a	n/a	experimental	Data retrived from review and not original research article.	Bergey's Manual of Systematic Bacteriology, Vol. 3-5, Edn. 2nd.	n/a	n/a	S4
Saccharibacteria (TM7) [G-1] sp. HMT-347	347	https://www.homd.org/taxa/tax_description?otid=347	DEPENDS_ON	Actinobacteria	taxon	n/a	n/a	n/a	n/a	prediction	Prediction based on the characteristics of Saccharibacteria (TM7) phylum. Data retrived from review and not original research article.	Bor2019 10.1177/0022034519831671	https://pubmed.ncbi.nlm.nih.gov/30894042/	n/a	S4
Schaalia odontolytica	701	https://www.homd.org/taxa/tax_description?otid=701	PRODUCES	DNAse	enzyme	n/a	n/a	n/a	n/a	experimental	Data retrived from review and not original research article. Some.	Bergey's Manual of Systematic Bacteriology, Vol. 3-5, Edn. 2nd.	n/a	n/a	S4
Schaalia odontolytica	701	https://www.homd.org/taxa/tax_description?otid=701	PRODUCES	fucosidase	enzyme	n/a	n/a	n/a	n/a	experimental	Data retrived from review and not original research article. Variable.	Bergey's Manual of Systematic Bacteriology, Vol. 3-5, Edn. 2nd.	n/a	n/a	S4
Schaalia odontolytica	701	https://www.homd.org/taxa/tax_description?otid=701	PRODUCES	lactic acid	metabolite	HMDB0000190	https://hmdb.ca/metabolites/HMDB0000190	n/a	n/a	experimental	Data retrived from review and not original research article.	Bergey's Manual of Systematic Bacteriology, Vol. 3-5, Edn. 2nd.	n/a	n/a	S4
Schaalia odontolytica	701	https://www.homd.org/taxa/tax_description?otid=701	PRODUCES	menaquinone	metabolite	n/a	n/a	n/a	n/a	experimental	Tetrahydrogenated menaquinones with ten isoprene units [MK-10(H4)] are the major respiratory quinones.	Fernandez1987 10.1111/j.1574-6968.1987.tb02191.x	n/a	n/a	
Schaalia odontolytica	701	https://www.homd.org/taxa/tax_description?otid=701	PRODUCES	nitrite	metabolite	HMDB0002786	https://hmdb.ca/metabolites/HMDB0002786	n/a	n/a	experimental	Data retrived from review and not original research article.	Bergey's Manual of Systematic Bacteriology, Vol. 3-5, Edn. 2nd.	n/a	n/a	S4
Schaalia odontolytica	701	https://www.homd.org/taxa/tax_description?otid=701	USES	L-arginine	metabolite	HMDB0000517	https://hmdb.ca/metabolites/HMDB0000517	n/a	n/a	experimental	Data retrived from review and not original research article.	Bergey's Manual of Systematic Bacteriology, Vol. 3-5, Edn. 2nd.	n/a	n/a	S4
Schaalia odontolytica	701	https://www.homd.org/taxa/tax_description?otid=701	USES	L-lysine	metabolite	HMDB0000182	https://hmdb.ca/metabolites/HMDB0000182	n/a	n/a	experimental	Data retrived from review and not original research article.	Bergey's Manual of Systematic Bacteriology, Vol. 3-5, Edn. 2nd.	n/a	n/a	S4
Schaalia odontolytica	701	https://www.homd.org/taxa/tax_description?otid=701	USES	nitrate	metabolite	HMDB0002878	https://hmdb.ca/metabolites/HMDB0002878	n/a	n/a	experimental	Data retrived from review and not original research article.	Bergey's Manual of Systematic Bacteriology, Vol. 3-5, Edn. 2nd.	n/a	n/a	S4
Schaalia sp. OTU_7	n/a	n/a	PRODUCES	DNAse	enzyme	n/a	n/a	n/a	n/a	prediction	Prediction based on the characteristics of related S. odontolytica. Data retrived from review and not original research article. Some.	Bergey's Manual of Systematic Bacteriology, Vol. 3-5, Edn. 2nd.	n/a	n/a	S4

Schaalia sp. OTU_7	n/a	n/a	PRODUCES	fucosidase	enzyme	n/a	n/a	n/a	n/a	prediction	Prediction based on the characteristics of related S. odontolytica. Data retrived from review and not original research article. Variable	Bergey's Manual of Systematic Bacteriology, Vol. 3-5, Edn. 2nd.	n/a	n/a	S4
Schaalia sp. OTU_7	n/a	n/a	PRODUCES	lactic acid	metabolite	HMDB0000190	https://hmdb.ca/metabolites/HMDB0000190	n/a	n/a	prediction	Prediction based on the characteristics of related S. odontolytica. Data retrived from review and not original research article.	Bergey's Manual of Systematic Bacteriology, Vol. 3-5, Edn. 2nd.	n/a	n/a	S4
Schaalia sp. OTU_7	n/a	n/a	PRODUCES	menaquinone	metabolite	n/a	n/a	n/a	n/a	prediction	Prediction based on the characteristics of Schaalia genus.Tetrahydrogenated menaquinones with ten isoprene units [MK-10(H4)] are the major respiratory quinones.	Fernandez1987 10.1111/j.1574-6968.1987.tb02191.x	n/a	n/a	
Schaalia sp. OTU_7	n/a	n/a	PRODUCES	nitrite	metabolite	HMDB0002786	https://hmdb.ca/metabolites/HMDB0002786	n/a	n/a	prediction	Prediction based on the characteristics of related S. odontolytica. Data retrived from review and not original research article.	Bergey's Manual of Systematic Bacteriology, Vol. 3-5, Edn. 2nd.	n/a	n/a	S4
Schaalia sp. OTU_7	n/a	n/a	USES	L-arginine	metabolite	HMDB0000517	https://hmdb.ca/metabolites/HMDB0000517	n/a	n/a	prediction	Prediction based on the characteristics of related S. odontolytica. Data retrived from review and not original research article.	Bergey's Manual of Systematic Bacteriology, Vol. 3-5, Edn. 2nd.	n/a	n/a	S4
Schaalia sp. OTU_7	n/a	n/a	USES	L-lysine	metabolite	HMDB0000182	https://hmdb.ca/metabolites/HMDB0000182	n/a	n/a	prediction	Prediction based on the characteristics of related S. odontolytica. Data retrived from review and not original research article.	Bergey's Manual of Systematic Bacteriology, Vol. 3-5, Edn. 2nd.	n/a	n/a	S4
Schaalia sp. OTU_7	n/a	n/a	USES	nitrate	metabolite	HMDB0002878	https://hmdb.ca/metabolites/HMDB0002878	n/a	n/a	prediction	Prediction based on the characteristics of related S. odontolytica. Data retrived from review and not original research article.	Bergey's Manual of Systematic Bacteriology, Vol. 3-5, Edn. 2nd.	n/a	n/a	S4
Schaalia sp._HMT_172	172	https://www.homd.org/taxa/tax_description?otid=172	PRODUCES	DNAse	enzyme	n/a	n/a	n/a	n/a	experimental	Data retrived from review and not original research article. Some.	Bergey's Manual of Systematic Bacteriology, Vol. 3-5, Edn. 2nd.	n/a	n/a	S4
Schaalia sp._HMT_172	172	https://www.homd.org/taxa/tax_description?otid=172	PRODUCES	fucosidase	enzyme	n/a	n/a	n/a	n/a	experimental	Prediction based on the characteristics of related S. odontolytica. Data retrived from review and not original research article. Variable.	Bergey's Manual of Systematic Bacteriology, Vol. 3-5, Edn. 2nd.	n/a	n/a	S4
Schaalia sp._HMT_172	172	https://www.homd.org/taxa/tax_description?otid=172	PRODUCES	lactic acid	metabolite	HMDB0000190	https://hmdb.ca/metabolites/HMDB0000190	n/a	n/a	experimental	Prediction based on the characteristics of related S. odontolytica. Data retrived from review and not original research article.	Bergey's Manual of Systematic Bacteriology, Vol. 3-5, Edn. 2nd.	n/a	n/a	S4
Schaalia sp._HMT_172	172	https://www.homd.org/taxa/tax_description?otid=172	PRODUCES	menaquinone	metabolite	n/a	n/a	n/a	n/a	prediction	Prediction based on the characteristics of Schaalia genus.Tetrahydrogenated menaquinones with ten isoprene units [MK-10(H4)] are the major respiratory quinones.	Fernandez1987 10.1111/j.1574-6968.1987.tb02191.x	n/a	n/a	
Schaalia sp._HMT_172	172	https://www.homd.org/taxa/tax_description?otid=172	PRODUCES	nitrite	metabolite	HMDB0002786	https://hmdb.ca/metabolites/HMDB0002786	n/a	n/a	experimental	Prediction based on the characteristics of related S. odontolytica. Data retrived from review and not original research article.	Bergey's Manual of Systematic Bacteriology, Vol. 3-5, Edn. 2nd.	n/a	n/a	S4
Schaalia sp._HMT_172	172	https://www.homd.org/taxa/tax_description?otid=172	USES	L-arginine	metabolite	HMDB0000517	https://hmdb.ca/metabolites/HMDB0000517	n/a	n/a	experimental	Prediction based on the characteristics of related S. odontolytica. Data retrived from review and not original research article.	Bergey's Manual of Systematic Bacteriology, Vol. 3-5, Edn. 2nd.	n/a	n/a	S4
Schaalia sp._HMT_172	172	https://www.homd.org/taxa/tax_description?otid=172	USES	L-lysine	metabolite	HMDB0000182	https://hmdb.ca/metabolites/HMDB0000182	n/a	n/a	experimental	Prediction based on the characteristics of related S. odontolytica. Data retrived from review and not original research article.	Bergey's Manual of Systematic Bacteriology, Vol. 3-5, Edn. 2nd.	n/a	n/a	S4
Schaalia sp._HMT_172	172	https://www.homd.org/taxa/tax_description?otid=172	USES	nitrate	metabolite	HMDB0002878	https://hmdb.ca/metabolites/HMDB0002878	n/a	n/a	experimental	Prediction based on the characteristics of related S. odontolytica. Data retrived from review and not original research article.	Bergey's Manual of Systematic Bacteriology, Vol. 3-5, Edn. 2nd.	n/a	n/a	S4
Selenomonas sp. OTU_20	n/a	n/a	IS_INHIBITED_BY	oxygen	metabolite	HMDB0001377	https://hmdb.ca/metabolites/HMDB0001377	n/a	n/a	prediction	Prediction based on the characteristics of Selenomonas genus. Data retrived from review and not original research article.	Bergey's Manual of Systematic Bacteriology, Vol. 3-5, Edn. 2nd.	n/a	n/a	S4
Streptocccus sp. HMT-64	64	https://www.homd.org/taxa/tax_description?otid=064	PRODUCES	hydrogen peroxide	metabolite	HMDB0003125	https://hmdb.ca/metabolites/HMDB0003125	n/a	n/a	prediction	Prediction based on the characteristics of related S. mitis/oralis. Data retrived from review and not original research article.	Bergey's Manual of Systematic Bacteriology, Vol. 3-5, Edn. 2nd.	n/a	n/a	S4
Streptocccus sp. HMT-64	64	https://www.homd.org/taxa/tax_description?otid=064	PRODUCES	lactic acid	metabolite	HMDB0000190	https://hmdb.ca/metabolites/HMDB0000190	n/a	n/a	experimental	Prediction based on the characteristics of related S. mitis/oralis. Data retrived from review and not original research article.	Bergey's Manual of Systematic Bacteriology, Vol. 3-5, Edn. 2nd.	n/a	n/a	S4
Streptocccus sp. HMT-64	64	https://www.homd.org/taxa/tax_description?otid=064	PRODUCES	peptides	metabolite	n/a	n/a	n/a	n/a	prediction	Prediction based on the characteristics of related S. mitis/oralis. Data retrived from review and not original research article.	Bergey's Manual of Systematic Bacteriology, Vol. 3-5, Edn. 2nd.	n/a	n/a	S4
Streptococcus gordonii	622	https://www.homd.org/taxa/tax_description?otid=622	PRODUCES	challisin	enzyme	n/a	n/a	3.4.21.96	https://www.genome.jp/entry/3.4.21.96	experimental	Data retrived from review and not original research article.	Bergey's Manual of Systematic Bacteriology, Vol. 3-5, Edn. 2nd.	n/a	n/a	S4
Streptococcus gordonii	622	https://www.homd.org/taxa/tax_description?otid=622	PRODUCES	DNAse	enzyme	n/a	n/a	n/a	n/a	experimental	Data retrived from review and not original research article.	Bergey's Manual of Systematic Bacteriology, Vol. 3-5, Edn. 2nd.	n/a	n/a	S4
Streptococcus gordonii	622	https://www.homd.org/taxa/tax_description?otid=622	PRODUCES	fucosidase	enzyme	n/a	n/a	n/a	n/a	experimental	Data retrived from review and not original research article.	Bergey's Manual of Systematic Bacteriology, Vol. 3-5, Edn. 2nd.	n/a	n/a	S4
Streptococcus gordonii	622	https://www.homd.org/taxa/tax_description?otid=622	PRODUCES	hydrogen peroxide	metabolite	HMDB0003125	https://hmdb.ca/metabolites/HMDB0003125	n/a	n/a	experimental	Data retrived from review and not original research article.	Bergey's Manual of Systematic Bacteriology, Vol. 3-5, Edn. 2nd.	n/a	n/a	S4
Streptococcus gordonii	622	https://www.homd.org/taxa/tax_description?otid=622	PRODUCES	lactic acid	metabolite	HMDB0000190	https://hmdb.ca/metabolites/HMDB0000190	n/a	n/a	experimental	Data retrived from review and not original research article.	Bergey's Manual of Systematic Bacteriology, Vol. 3-5, Edn. 2nd.	n/a	n/a	S4
Streptococcus gordonii	622	https://www.homd.org/taxa/tax_description?otid=622	PRODUCES	L-ornithine	metabolite	HMDB0000214	https://hmdb.ca/metabolites/HMDB0000214	n/a	n/a	experimental		Sakanaka2022 10.1128/msystems.00170-22	https://pubmed.ncbi.nlm.nih.gov/35852319/	n/a	S4
Streptococcus gordonii	622	https://www.homd.org/taxa/tax_description?otid=622	USES	L-arginine	metabolite	HMDB0000517	https://hmdb.ca/metabolites/HMDB0000517	n/a	n/a	experimental	Data retrived from review and not original research article.	Bergey's Manual of Systematic Bacteriology, Vol. 3-5, Edn. 2nd.	n/a	n/a	S4
Streptococcus mitis/oralis group	n/a	n/a	PRODUCES	hydrogen peroxide	metabolite	HMDB0003125	https://hmdb.ca/metabolites/HMDB0003125	n/a	n/a	experimental	Data retrived from review and not original research article.	Bergey's Manual of Systematic Bacteriology, Vol. 3-5, Edn. 2nd.	n/a	n/a	S4
Streptococcus mitis/oralis group	n/a	n/a	PRODUCES	IgA1 protease	enzyme	n/a	n/a	3.4.21.-	n/a	experimental	Data retrived from review and not original research article.	Bergey's Manual of Systematic Bacteriology, Vol. 3-5, Edn. 2nd.	n/a	n/a	S4
Streptococcus mitis/oralis group	n/a	n/a	PRODUCES	lactic acid	metabolite	HMDB0000190	https://hmdb.ca/metabolites/HMDB0000190	n/a	n/a	experimental	Data retrived from review and not original research article.	Bergey's Manual of Systematic Bacteriology, Vol. 3-5, Edn. 2nd.	n/a	n/a	S4
Streptococcus mitis/oralis group	n/a	n/a	PRODUCES	peptides	metabolite	n/a	n/a	n/a	n/a	experimental	Data retrived from review and not original research article.	Bergey's Manual of Systematic Bacteriology, Vol. 3-5, Edn. 2nd.	n/a	n/a	S4
Streptococcus mitis/oralis group	n/a	n/a	PRODUCES	sialidase	enzyme	n/a	n/a	3.2.1.18	https://www.genome.jp/entry/3.2.1.18	experimental	Data retrived from review and not original research article.	Bergey's Manual of Systematic Bacteriology, Vol. 3-5, Edn. 2nd.	n/a	n/a	S4
Streptococcus mitis/oralis group	n/a	n/a	USES	nitrite	metabolite	HMDB0002786	https://hmdb.ca/metabolites/HMDB0002786	n/a	n/a	experimental	Data retrived from review and not original research article.	Bergey's Manual of Systematic Bacteriology, Vol. 3-5, Edn. 2nd.	n/a	n/a	S4
Streptococcus salivarius	755	https://www.homd.org/taxa/tax_description?otid=755	HYDROLYSES	urea	metabolite	HMDB0000294	https://hmdb.ca/metabolites/HMDB0000294	n/a	n/a	experimental	Data retrived from review and not original research article. Some.	Bergey's Manual of Systematic Bacteriology, Vol. 3-5, Edn. 2nd.	n/a	n/a	S4
Streptococcus salivarius	755	https://www.homd.org/taxa/tax_description?otid=755	PRODUCES	lactic acid	metabolite	HMDB0000190	https://hmdb.ca/metabolites/HMDB0000190	n/a	n/a	experimental	Data retrived from review and not original research article.	Bergey's Manual of Systematic Bacteriology, Vol. 3-5, Edn. 2nd.	n/a	n/a	S4
Streptococcus salivarius	755	https://www.homd.org/taxa/tax_description?otid=755	USES	nitrite	metabolite	HMDB0002786	https://hmdb.ca/metabolites/HMDB0002786	n/a	n/a	experimental	Data retrived from review and not original research article.	Bergey's Manual of Systematic Bacteriology, Vol. 3-5, Edn. 2nd.	n/a	n/a	S4
Streptococcus sanguinis	758	https://www.homd.org/taxa/tax_description?otid=758	PRODUCES	hydrogen peroxide	metabolite	HMDB0003125	https://hmdb.ca/metabolites/HMDB0003125	n/a	n/a	experimental	Data retrived from review and not original research article.	Bergey's Manual of Systematic Bacteriology, Vol. 3-5, Edn. 2nd.	n/a	n/a	S4
Streptococcus sanguinis	758	https://www.homd.org/taxa/tax_description?otid=758	PRODUCES	IgA1 protease	enzyme	n/a	n/a	3.4.21.-	n/a	experimental	Data retrived from review and not original research article.	Bergey's Manual of Systematic Bacteriology, Vol. 3-5, Edn. 2nd.	n/a	n/a	S4
Streptococcus sanguinis	758	https://www.homd.org/taxa/tax_description?otid=758	PRODUCES	lactic acid	metabolite	HMDB0000190	https://hmdb.ca/metabolites/HMDB0000190	n/a	n/a	experimental	Data retrived from review and not original research article.	Bergey's Manual of Systematic Bacteriology, Vol. 3-5, Edn. 2nd.	n/a	n/a	S4
Streptococcus sanguinis	758	https://www.homd.org/taxa/tax_description?otid=758	PRODUCES	L-ornithine	metabolite	HMDB0000214	https://hmdb.ca/metabolites/HMDB0000214	n/a	n/a	experimental	Data retrived from review and not original research article.	Bergey's Manual of Systematic Bacteriology, Vol. 3-5, Edn. 2nd.	n/a	n/a	S4
Streptococcus sanguinis	758	https://www.homd.org/taxa/tax_description?otid=758	PRODUCES	peptides	metabolite	n/a	n/a	n/a	n/a	experimental	Data retrived from review and not original research article.	Bergey's Manual of Systematic Bacteriology, Vol. 3-5, Edn. 2nd.	n/a	n/a	S4
Streptococcus sanguinis	758	https://www.homd.org/taxa/tax_description?otid=758	USES	L-arginine	metabolite	HMDB0000517	https://hmdb.ca/metabolites/HMDB0000517	n/a	n/a	experimental	Data retrived from review and not original research article.	Bergey's Manual of Systematic Bacteriology, Vol. 3-5, Edn. 2nd.	n/a	n/a	S4
Streptococcus sp. OTU_10	n/a	n/a	HYDROLYSES	urea	metabolite	HMDB0000294	https://hmdb.ca/metabolites/HMDB0000294	n/a	n/a	prediction	Prediction based on the characteristics of related S. salivarius. Data retrived from review and not original research article. Some.	Bergey's Manual of Systematic Bacteriology, Vol. 3-5, Edn. 2nd.	n/a	n/a	S4
Streptococcus sp. OTU_10	n/a	n/a	PRODUCES	lactic acid	metabolite	HMDB0000190	https://hmdb.ca/metabolites/HMDB0000190	n/a	n/a	prediction	Prediction based on the characteristics of related S. salivarius. Data retrived from review and not original research article.	Bergey's Manual of Systematic Bacteriology, Vol. 3-5, Edn. 2nd.	n/a	n/a	S4
Streptococcus sp. OTU_10	n/a	n/a	USES	nitrite	metabolite	HMDB0002786	https://hmdb.ca/metabolites/HMDB0002786	n/a	n/a	prediction	Prediction based on the characteristics of related S. salivarius. Data retrived from review and not original research article.	Bergey's Manual of Systematic Bacteriology, Vol. 3-5, Edn. 2nd.	n/a	n/a	S4
Streptococcus sp. OTU_13	n/a	n/a	IS_INHIBITED_BY	oxygen	metabolite	HMDB0001377	https://hmdb.ca/metabolites/HMDB0001377	n/a	n/a	prediction	Prediction based on the characteristics of related S. anginosus. Data retrived from review and not original research article. Some.	Bergey's Manual of Systematic Bacteriology, Vol. 3-5, Edn. 2nd.	n/a	n/a	S4
Streptococcus sp. OTU_13	n/a	n/a	PRODUCES	L-ornithine	metabolite	HMDB0000214	https://hmdb.ca/metabolites/HMDB0000214	n/a	n/a	prediction	Prediction based on the characteristics of related S. anginosus. Data retrived from review and not original research article.	Bergey's Manual of Systematic Bacteriology, Vol. 3-5, Edn. 2nd.	n/a	n/a	S4
Streptococcus sp. OTU_13	n/a	n/a	USES	carbon dioxide	metabolite	HMDB0001967	https://hmdb.ca/metabolites/HMDB0001967	n/a	n/a	prediction	Prediction based on the characteristics of related S. anginosus. Data retrived from review and not original research article.	Bergey's Manual of Systematic Bacteriology, Vol. 3-5, Edn. 2nd.	n/a	n/a	S4
Streptococcus sp. OTU_13	n/a	n/a	USES	L-arginine	metabolite	HMDB0000517	https://hmdb.ca/metabolites/HMDB0000517	n/a	n/a	prediction	Prediction based on the characteristics of related S. anginosus. Data retrived from review and not original research article.	Bergey's Manual of Systematic Bacteriology, Vol. 3-5, Edn. 2nd.	n/a	n/a	S4
Streptococcus sp. OTU_144	n/a	n/a	PRODUCES	hydrogen peroxide	metabolite	HMDB0003125	https://hmdb.ca/metabolites/HMDB0003125	n/a	n/a	prediction	Prediction based on the characteristics of related S. mitis/oralis. Data retrived from review and not original research article.	Bergey's Manual of Systematic Bacteriology, Vol. 3-5, Edn. 2nd.	n/a	n/a	S4
Streptococcus sp. OTU_144	n/a	n/a	PRODUCES	IgA1 protease	enzyme	n/a	n/a	3.4.21.-	n/a	prediction	Prediction based on the characteristics of related S. mitis/oralis. Data retrived from review and not original research article.	Bergey's Manual of Systematic Bacteriology, Vol. 3-5, Edn. 2nd.	n/a	n/a	S4
Streptococcus sp. OTU_144	n/a	n/a	PRODUCES	lactic acid	metabolite	HMDB0000190	https://hmdb.ca/metabolites/HMDB0000190	n/a	n/a	prediction	Prediction based on the characteristics of related S. mitis/oralis. Data retrived from review and not original research article.	Bergey's Manual of Systematic Bacteriology, Vol. 3-5, Edn. 2nd.	n/a	n/a	S4
Streptococcus sp. OTU_144	n/a	n/a	PRODUCES	peptides	metabolite	n/a	n/a	n/a	n/a	prediction	Prediction based on the characteristics of related S. mitis/oralis. Data retrived from review and not original research article.	Bergey's Manual of Systematic Bacteriology, Vol. 3-5, Edn. 2nd.	n/a	n/a	S4
Streptococcus sp. OTU_144	n/a	n/a	PRODUCES	sialidase	enzyme	n/a	n/a	3.2.1.18	https://www.genome.jp/entry/3.2.1.18	prediction	Prediction based on the characteristics of related S. mitis/oralis. Data retrived from review and not original research article.	Bergey's Manual of Systematic Bacteriology, Vol. 3-5, Edn. 2nd.	n/a	n/a	S4
Streptococcus sp. OTU_144	n/a	n/a	USES	nitrite	metabolite	HMDB0002786	https://hmdb.ca/metabolites/HMDB0002786	n/a	n/a	prediction	Prediction based on the characteristics of related S. mitis/oralis. Data retrived from review and not original research article.	Bergey's Manual of Systematic Bacteriology, Vol. 3-5, Edn. 2nd.	n/a	n/a	S4
Streptococcus sp. OTU_17	n/a	n/a	PRODUCES	lactic acid	metabolite	HMDB0000190	https://hmdb.ca/metabolites/HMDB0000190	n/a	n/a	prediction	Prediction based on the characteristics of related S. australis. Data retrived from review and not original research article.	Bergey's Manual of Systematic Bacteriology, Vol. 3-5, Edn. 2nd.	n/a	n/a	S4
Streptococcus sp. OTU_17	n/a	n/a	PRODUCES	L-ornithine	metabolite	HMDB0000214	https://hmdb.ca/metabolites/HMDB0000214	n/a	n/a	prediction	Prediction based on the characteristics of related S. australis. Data retrived from review and not original research article.	Bergey's Manual of Systematic Bacteriology, Vol. 3-5, Edn. 2nd.	n/a	n/a	S4
Streptococcus sp. OTU_17	n/a	n/a	USES	L-arginine	metabolite	HMDB0000517	https://hmdb.ca/metabolites/HMDB0000517	n/a	n/a	prediction	Prediction based on the characteristics of related S. australis. Data retrived from review and not original research article.	Bergey's Manual of Systematic Bacteriology, Vol. 3-5, Edn. 2nd.	n/a	n/a	S4
Streptococcus sp. OTU_2	n/a	n/a	PRODUCES	hydrogen peroxide	metabolite	HMDB0003125	https://hmdb.ca/metabolites/HMDB0003125	n/a	n/a	prediction	Prediction based on the characteristics of related S. sanguinis. Data retrived from review and not original research article.	Bergey's Manual of Systematic Bacteriology, Vol. 3-5, Edn. 2nd.	n/a	n/a	S4
Streptococcus sp. OTU_2	n/a	n/a	PRODUCES	IgA1 protease	enzyme	n/a	n/a	3.4.21.-	n/a	prediction	Prediction based on the characteristics of related S. sanguinis. Data retrived from review and not original research article.	Bergey's Manual of Systematic Bacteriology, Vol. 3-5, Edn. 2nd.	n/a	n/a	S4
Streptococcus sp. OTU_2	n/a	n/a	PRODUCES	lactic acid	metabolite	HMDB0000190	https://hmdb.ca/metabolites/HMDB0000190	n/a	n/a	prediction	Prediction based on the characteristics of related S. sanguinis. Data retrived from review and not original research article.	Bergey's Manual of Systematic Bacteriology, Vol. 3-5, Edn. 2nd.	n/a	n/a	S4
Streptococcus sp. OTU_2	n/a	n/a	PRODUCES	L-ornithine	metabolite	HMDB0000214	https://hmdb.ca/metabolites/HMDB0000214	n/a	n/a	prediction	Prediction based on the characteristics of related S. sanguinis. Data retrived from review and not original research article.	Bergey's Manual of Systematic Bacteriology, Vol. 3-5, Edn. 2nd.	n/a	n/a	S4
Streptococcus sp. OTU_2	n/a	n/a	PRODUCES	peptides	metabolite	n/a	n/a	n/a	n/a	prediction	Prediction based on the characteristics of related S. sanguinis. Data retrived from review and not original research article.	Bergey's Manual of Systematic Bacteriology, Vol. 3-5, Edn. 2nd.	n/a	n/a	S4
Streptococcus sp. OTU_2	n/a	n/a	USES	L-arginine	metabolite	HMDB0000517	https://hmdb.ca/metabolites/HMDB0000517	n/a	n/a	prediction	Prediction based on the characteristics of related S. sanguinis. Data retrived from review and not original research article.	Bergey's Manual of Systematic Bacteriology, Vol. 3-5, Edn. 2nd.	n/a	n/a	S4
Streptococcus sp. OTU_9	n/a	n/a	PRODUCES	challisin	enzyme	n/a	n/a	n/a	n/a	prediction	Prediction based on the characteristics of related S. gordonii. Data retrived from review and not original research article.	Bergey's Manual of Systematic Bacteriology, Vol. 3-5, Edn. 2nd.	n/a	n/a	S4
Streptococcus sp. OTU_9	n/a	n/a	PRODUCES	DNAse	enzyme	n/a	n/a	n/a	n/a	prediction	Prediction based on the characteristics of related S. gordonii. Data retrived from review and not original research article.	Bergey's Manual of Systematic Bacteriology, Vol. 3-5, Edn. 2nd.	n/a	n/a	S4
Streptococcus sp. OTU_9	n/a	n/a	PRODUCES	fucosidase	enzyme	n/a	n/a	n/a	n/a	prediction	Prediction based on the characteristics of related S. gordonii. Data retrived from review and not original research article.	Bergey's Manual of Systematic Bacteriology, Vol. 3-5, Edn. 2nd.	n/a	n/a	S4
Streptococcus sp. OTU_9	n/a	n/a	PRODUCES	hydrogen peroxide	metabolite	HMDB0003125	https://hmdb.ca/metabolites/HMDB0003125	n/a	n/a	prediction	Prediction based on the characteristics of related S. gordonii. Data retrived from review and not original research article.	Bergey's Manual of Systematic Bacteriology, Vol. 3-5, Edn. 2nd.	n/a	n/a	S4
Streptococcus sp. OTU_9	n/a	n/a	PRODUCES	lactic acid	metabolite	HMDB0000190	https://hmdb.ca/metabolites/HMDB0000190	n/a	n/a	prediction	Prediction based on the characteristics of related S. gordonii. Data retrived from review and not original research article.	Bergey's Manual of Systematic Bacteriology, Vol. 3-5, Edn. 2nd.	n/a	n/a	S4
Streptococcus sp. OTU_9	n/a	n/a	PRODUCES	L-ornithine	metabolite	HMDB0000214	https://hmdb.ca/metabolites/HMDB0000214	n/a	n/a	prediction	Prediction based on the characteristics of related S. gordonii.	Sakanaka2022 10.1128/msystems.00170-22	https://pubmed.ncbi.nlm.nih.gov/35852319/	n/a	S4
Streptococcus sp. OTU_9	n/a	n/a	USES	L-arginine	metabolite	HMDB0000517	https://hmdb.ca/metabolites/HMDB0000517	n/a	n/a	prediction	Prediction based on the characteristics of related S. gordonii. Data retrived from review and not original research article.	Bergey's Manual of Systematic Bacteriology, Vol. 3-5, Edn. 2nd.	n/a	n/a	S4
Veillonella parvula	161	https://www.homd.org/taxa/tax_description?otid=161	IS_INHIBITED_BY	oxygen	metabolite	HMDB0001377	https://hmdb.ca/metabolites/HMDB0001377	n/a	n/a	experimental	Data retrived from review and not original research article.	Bergey's Manual of Systematic Bacteriology, Vol. 3-5, Edn. 2nd.	n/a	n/a	S4
Veillonella parvula	161	https://www.homd.org/taxa/tax_description?otid=161	PRODUCES	carbon dioxide	metabolite	HMDB0001967	https://hmdb.ca/metabolites/HMDB0001967	n/a	n/a	experimental	Data retrived from review and not original research article.	Bergey's Manual of Systematic Bacteriology, Vol. 3-5, Edn. 2nd.	n/a	n/a	S4
Veillonella parvula	161	https://www.homd.org/taxa/tax_description?otid=161	PRODUCES	catalase	enzyme	n/a	n/a	1.11.1.6	https://www.genome.jp/entry/1.11.1.6	experimental	Data retrived from review and not original research article. Some.	Bergey's Manual of Systematic Bacteriology, Vol. 3-5, Edn. 2nd.	n/a	n/a	S4
Veillonella parvula	161	https://www.homd.org/taxa/tax_description?otid=161	PRODUCES	hydrogen	metabolite	HMDB0001362	https://hmdb.ca/metabolites/HMDB0001362	n/a	n/a	experimental	Data retrived from review and not original research article.	Bergey's Manual of Systematic Bacteriology, Vol. 3-5, Edn. 2nd.	n/a	n/a	S4
Veillonella parvula	161	https://www.homd.org/taxa/tax_description?otid=161	PRODUCES	hydrogen sulfide	metabolite	HMDB0003276	https://hmdb.ca/metabolites/HMDB0003276	n/a	n/a	experimental	Data retrived from review and not original research article.	Bergey's Manual of Systematic Bacteriology, Vol. 3-5, Edn. 2nd.	n/a	n/a	S4
Veillonella parvula	161	https://www.homd.org/taxa/tax_description?otid=161	PRODUCES	menaquinone	metabolite	n/a	n/a	n/a	n/a	experimental	Menaquinones with seven isoprene units [MK-7)] are the major respiratory quinones.	Fernandez1987 10.1111/j.1574-6968.1987.tb02191.x	n/a	n/a	
Veillonella parvula	161	https://www.homd.org/taxa/tax_description?otid=161	PRODUCES	nitrite	metabolite	HMDB0002786	https://hmdb.ca/metabolites/HMDB0002786	n/a	n/a	experimental	Data retrived from review and not original research article.	Bergey's Manual of Systematic Bacteriology, Vol. 3-5, Edn. 2nd.	n/a	n/a	S4
Veillonella parvula	161	https://www.homd.org/taxa/tax_description?otid=161	RELEASES	hemin	metabolite	HMDB0000887	https://hmdb.ca/metabolites/HMDB0000887	n/a	n/a	experimental	Data retrived from review and not original research article.	Bergey's Manual of Systematic Bacteriology, Vol. 3-5, Edn. 2nd.	n/a	n/a	S4
Veillonella parvula	161	https://www.homd.org/taxa/tax_description?otid=161	RELEASES	L-lysine	metabolite	HMDB0000182	https://hmdb.ca/metabolites/HMDB0000182	n/a	n/a	experimental		Sakanaka2022 10.1128/msystems.00170-22	https://pubmed.ncbi.nlm.nih.gov/35852319/	n/a	S4
Veillonella parvula	161	https://www.homd.org/taxa/tax_description?otid=161	USES	lactic acid	metabolite	HMDB0000190	https://hmdb.ca/metabolites/HMDB0000190	n/a	n/a	experimental	Data retrived from review and not original research article.	Bergey's Manual of Systematic Bacteriology, Vol. 3-5, Edn. 2nd.	n/a	n/a	S4
Veillonella parvula	161	https://www.homd.org/taxa/tax_description?otid=161	USES	L-cysteine	metabolite	HMDB0000574	https://hmdb.ca/metabolites/HMDB0000574	n/a	n/a	experimental	Data retrived from review and not original research article.	Bergey's Manual of Systematic Bacteriology, Vol. 3-5, Edn. 2nd.	n/a	n/a	S4
Veillonella parvula	161	https://www.homd.org/taxa/tax_description?otid=161	USES	nitrate	metabolite	HMDB0002878	https://hmdb.ca/metabolites/HMDB0002878	n/a	n/a	experimental	Data retrived from review and not original research article.	Bergey's Manual of Systematic Bacteriology, Vol. 3-5, Edn. 2nd.	n/a	n/a	S4
Veillonella sp. OTU_16	n/a	n/a	IS_INHIBITED_BY	oxygen	metabolite	HMDB0001377	https://hmdb.ca/metabolites/HMDB0001377	n/a	n/a	prediction	Prediction based on the characteristics of Veillonella genus. Data retrived from review and not original research article.	Bergey's Manual of Systematic Bacteriology, Vol. 3-5, Edn. 2nd.	n/a	n/a	S4
Veillonella sp. OTU_16	n/a	n/a	PRODUCES	carbon dioxide	metabolite	HMDB0001967	https://hmdb.ca/metabolites/HMDB0001967	n/a	n/a	prediction	Prediction based on the characteristics of Veillonella genus. Data retrived from review and not original research article.	Bergey's Manual of Systematic Bacteriology, Vol. 3-5, Edn. 2nd.	n/a	n/a	S4
Veillonella sp. OTU_16	n/a	n/a	PRODUCES	catalase	enzyme	n/a	n/a	1.11.1.6	https://www.genome.jp/entry/1.11.1.6	prediction	Prediction based on the characteristics of Veillonella genus. Some. Data retrived from review and not original research article.	Bergey's Manual of Systematic Bacteriology, Vol. 3-5, Edn. 2nd.	n/a	n/a	S4
Veillonella sp. OTU_16	n/a	n/a	PRODUCES	hydrogen	metabolite	HMDB0001362	https://hmdb.ca/metabolites/HMDB0001362	n/a	n/a	prediction	Prediction based on the characteristics of Veillonella genus. Data retrived from review and not original research article.	Bergey's Manual of Systematic Bacteriology, Vol. 3-5, Edn. 2nd.	n/a	n/a	S4
Veillonella sp. OTU_16	n/a	n/a	PRODUCES	hydrogen sulfide	metabolite	HMDB0003276	https://hmdb.ca/metabolites/HMDB0003276	n/a	n/a	prediction	Prediction based on the characteristics of Veillonella genus. Data retrived from review and not original research article.	Bergey's Manual of Systematic Bacteriology, Vol. 3-5, Edn. 2nd.	n/a	n/a	S4
Veillonella sp. OTU_16	n/a	n/a	PRODUCES	menaquinone	metabolite	n/a	n/a	n/a	n/a	prediction	Prediction based on the characteristics of Veillonella genus. Menaquinones with seven isoprene units [MK-7)] are the major respiratory quinones.	Fernandez1987 10.1111/j.1574-6968.1987.tb02191.x	n/a	n/a	
Veillonella sp. OTU_16	n/a	n/a	PRODUCES	nitrite	metabolite	HMDB0002786	https://hmdb.ca/metabolites/HMDB0002786	n/a	n/a	prediction	Prediction based on the characteristics of Veillonella genus. Data retrived from review and not original research article.	Bergey's Manual of Systematic Bacteriology, Vol. 3-5, Edn. 2nd.	n/a	n/a	S4
Veillonella sp. OTU_16	n/a	n/a	RELEASES	hemin	metabolite	HMDB0000887	https://hmdb.ca/metabolites/HMDB0000887	n/a	n/a	prediction	Prediction based on the characteristics of Veillonella genus. Data retrived from review and not original research article.	Bergey's Manual of Systematic Bacteriology, Vol. 3-5, Edn. 2nd.	n/a	n/a	S4
Veillonella sp. OTU_16	n/a	n/a	RELEASES	L-lysine	metabolite	HMDB0000182	https://hmdb.ca/metabolites/HMDB0000182	n/a	n/a	prediction	Prediction based on the characteristics of Veillonella genus.	Sakanaka2022 10.1128/msystems.00170-22	https://pubmed.ncbi.nlm.nih.gov/35852319/	n/a	S4
Veillonella sp. OTU_16	n/a	n/a	USES	lactic acid	metabolite	HMDB0000190	https://hmdb.ca/metabolites/HMDB0000190	n/a	n/a	prediction	Prediction based on the characteristics of Veillonella genus. Data retrived from review and not original research article.	Bergey's Manual of Systematic Bacteriology, Vol. 3-5, Edn. 2nd.	n/a	n/a	S4
Veillonella sp. OTU_16	n/a	n/a	USES	L-cysteine	metabolite	HMDB0000574	https://hmdb.ca/metabolites/HMDB0000574	n/a	n/a	prediction	Prediction based on the characteristics of Veillonella genus. Data retrived from review and not original research article.	Bergey's Manual of Systematic Bacteriology, Vol. 3-5, Edn. 2nd.	n/a	n/a	S4
Veillonella sp. OTU_16	n/a	n/a	USES	nitrate	metabolite	HMDB0002878	https://hmdb.ca/metabolites/HMDB0002878	n/a	n/a	prediction	Prediction based on the characteristics of Veillonella genus. Data retrived from review and not original research article.	Bergey's Manual of Systematic Bacteriology, Vol. 3-5, Edn. 2nd.	n/a	n/a	S4
